# Supplementary material for: Anti-Food Allergic Compounds from Penicillium griseofulvum MCCC 3A00225, a Deep-Sea-Derived Fungus
Source: Mar Drugs. 2021 Apr 16;19(4):224. doi: 10.3390/md19040224 (PMC8073018; doi:10.3390/md19040224)
Supplement: Supplementary file 1 [file marinedrugs-19-00224-s001.zip › marinedrugs-1177355-supplementary.pdf]

marine drugs MDPI

Supplementary Material

Anti-Food Allergic Compounds from *Penicillium griseofulvum* MCCC 3A00225, a Deep-Sea-Derived Fungus

Cui-Ping Xing [1], Dan Chen [2], Chun-Lan Xie [1], Qingmei Liu [3], Tian-Hua Zhong [1], Zongze Shao [1], Guangming Liu [3], Lian-Zhong Luo [2,*] and Xian-Wen Yang [1,*]

[1] Key Laboratory of Marine Biogenetic Resources, Third Institute of Oceanography, Ministry of Natural Resources,184 Daxue Road, Xiamen 361005, China; xingcuiping123@126.com (C.-P. X.); xiechunlanxx@163.com (C.-L.X.); zhongtianhua@tio.org.cn (T.-H. Z.); shaozongze@tio.org.cn (Z. S.)

[2] Fujian Universities and colleges engineering research center of marine biopharmaceutical resources, Xiamen Medical College, 1999 Guankouzhong Road, Xiamen 361023, China; cd@xmmc.edu.cn (D.C.)

[3] College of Food and Biological Engineering, Jimei University, 43 Yindou Road, Xiamen 361021, Fujian, P. R. China; 435782572@qq.com (Q.L.); gmliu@jmu.edu.cn (G.L.)

Content

Figure S1. ^{1}H NMR spectrum of **1** in CD_3OD.
Figure S2. ^{13}C NMR spectrum of **1** in CD_3OD.
Figure S3. HSQC NMR spectrum of **1** in CD_3OD.
Figure S4. COSY NMR spectrum of **1** in CD_3OD.
Figure S5. HMBC NMR spectrum of **1** in CD_3OD.
Figure S6. NOESY NMR spectrum of **1** in CD_3OD.
Figure S7. ^{1}H NMR spectrum of **2** in CD_3OD.
Figure S8. ^{13}C NMR spectrum of **2** in CD_3OD.
Figure S9. HSQC NMR spectrum of **2** in CD_3OD.
Figure S10. COSY NMR spectrum of **2** in CD_3OD.
Figure S11. HMBC NMR spectrum of **2** in CD_3OD.
Figure S12. NOESY NMR spectrum of **2** in CD_3OD.
Figure S13. ^{1}H NMR spectrum of **3** in CD_3OD.
Figure S14. ^{13}C NMR spectrum of **3** in CD_3OD.
Figure S15. HSQC NMR spectrum of **3** in CD_3OD.
Figure S16. COSY NMR spectrum of **3** in CD_3OD.
Figure S17. HMBC NMR spectrum of **3** in CD_3OD.
Figure S18. NOESY NMR spectrum of **3** in CD_3OD.
Figure S19. ^{1}H NMR spectrum of **4** in CD_3OD.
Figure S20. ^{13}C NMR spectrum of **4** in CD_3OD.
Figure S21. HSQC NMR spectrum of **4** in CD_3OD.
Figure S22. COSY NMR spectrum of **4** in CD_3OD.

Figure S23. HMBC NMR spectrum of **4** in CD_3OD.
Figure S24. NOESY NMR spectrum of **4** in CD_3OD.
Figure S25. ^1H NMR spectrum of **5** in CD_3OD.
Figure S26. ^{13}C NMR spectrum of **5** in CD_3OD.
Figure S27. HSQC NMR spectrum of **5** in CD_3OD.
Figure S28. COSY NMR spectrum of **5** in CD_3OD.
Figure S29. HMBC NMR spectrum of **5** in CD_3OD.
Figure S30. NOESY NMR spectrum of **5** in CD_3OD.
Figure S31. ^1H NMR spectrum of **6** in CD_3OD.
Figure S32. ^{13}C NMR spectrum of **6** in CD_3OD.
Figure S33. HSQC NMR spectrum of **6** in CD_3OD.
Figure S34. COSY NMR spectrum of **6** in CD_3OD.
Figure S35. HMBC NMR spectrum of **6** in CD_3OD.
Figure S36. NOESY NMR spectrum of **6** in CD_3OD.
Figure S37. ^1H NMR spectrum of **7** in DMSO-d_6.
Figure S38. ^{13}C NMR spectrum of **7** in DMSO-d_6.
Figure S39. HSQC NMR spectrum of **7** in DMSO-d_6.
Figure S40. COSY NMR spectrum of **7** in DMSO-d_6.
Figure S41. HMBC NMR spectrum of **7** in DMSO-d_6.
Figure S42. NOESY NMR spectrum of **7** in DMSO-d_6.
Figure S43. ^1H NMR spectrum of **8** in CD_3OD.
Figure S44. ^{13}C NMR spectrum of **8** in CD_3OD.
Figure S45. HSQC NMR spectrum of **8** in CD_3OD.
Figure S46. COSY NMR spectrum of **8** in CD_3OD.
Figure S47. HMBC NMR spectrum of **8** in CD_3OD.
Figure S48. NOESY NMR spectrum of **8** in CD_3OD.
Figure S49. ^1H NMR spectrum of **9** in CD_3OD.
Figure S50. ^{13}C NMR spectrum of **9** in CD_3OD.
Figure S51. HSQC NMR spectrum of **9** in CD_3OD.
Figure S52. COSY NMR spectrum of **9** in CD_3OD.
Figure S53. HMBC NMR spectrum of **9** in CD_3OD.
Figure S54. NOESY NMR spectrum of **9** in CD_3OD.
Figure S55. ^1H NMR spectrum of **10** in CD_3OD.
Figure S56. ^{13}C NMR spectrum of **10** in CD_3OD.
Figure S57. HSQC NMR spectrum of **10** in CD_3OD.
Figure S58. COSY NMR spectrum of **10** in CD_3OD.
Figure S50. HMBC NMR spectrum of **10** in CD_3OD.
Figure S60. NOESY NMR spectrum of **10** in CD_3OD.

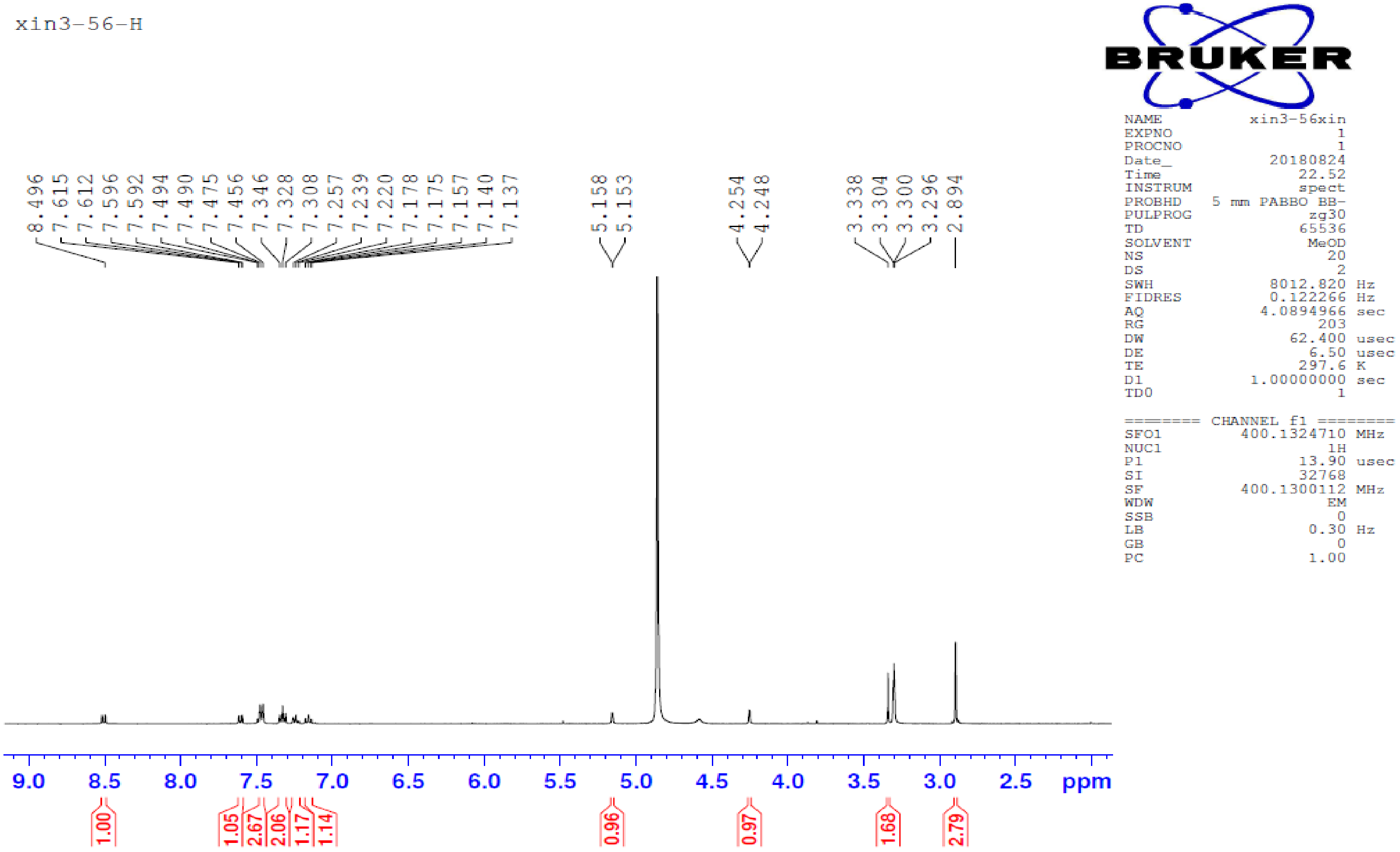

Figure S1. ^{1}H NMR spectrum of **1** in CD_3OD.

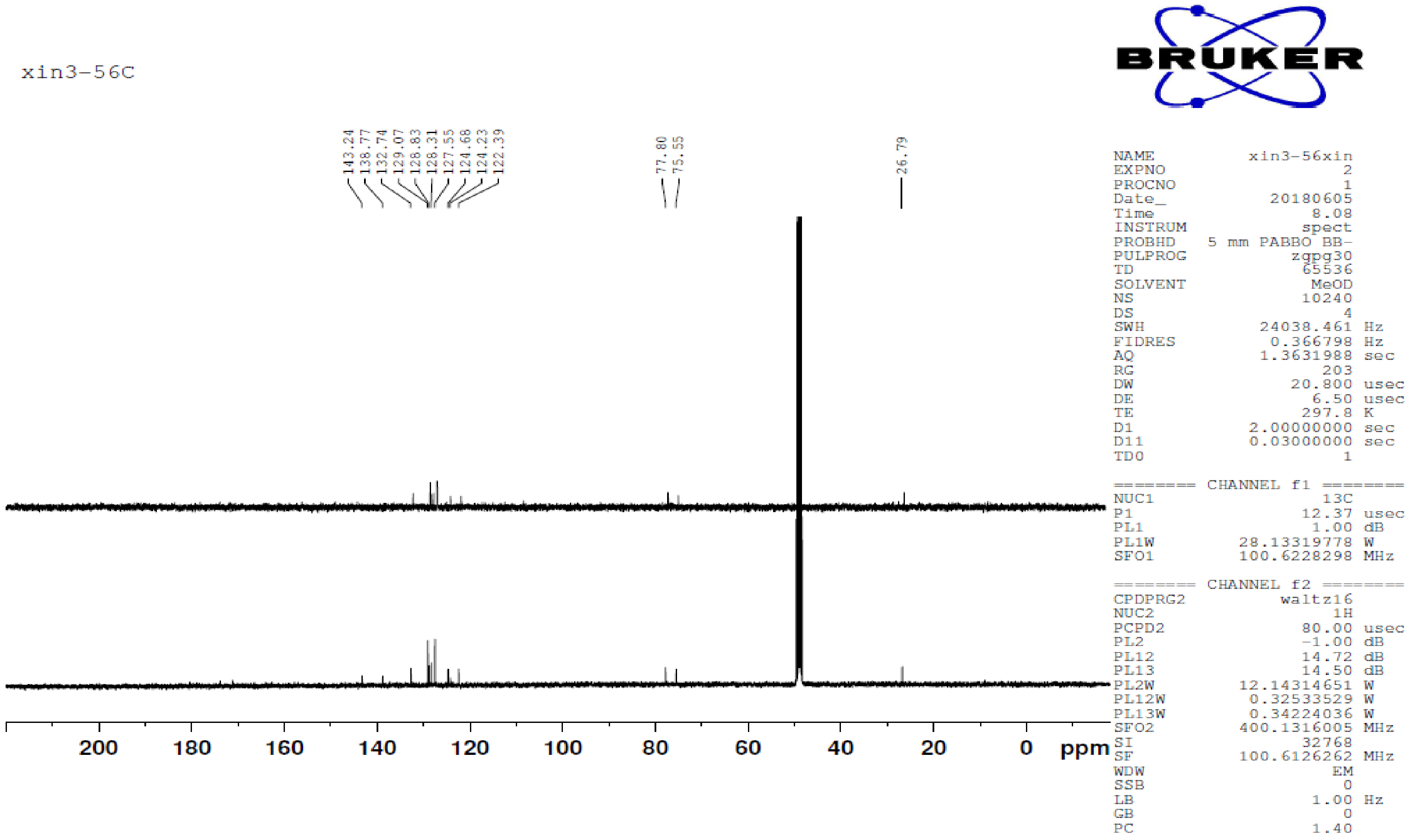

Figure S2. ^{13}C NMR spectrum of **1** in CD$_3$OD.（两个 170 多的位置未标出）

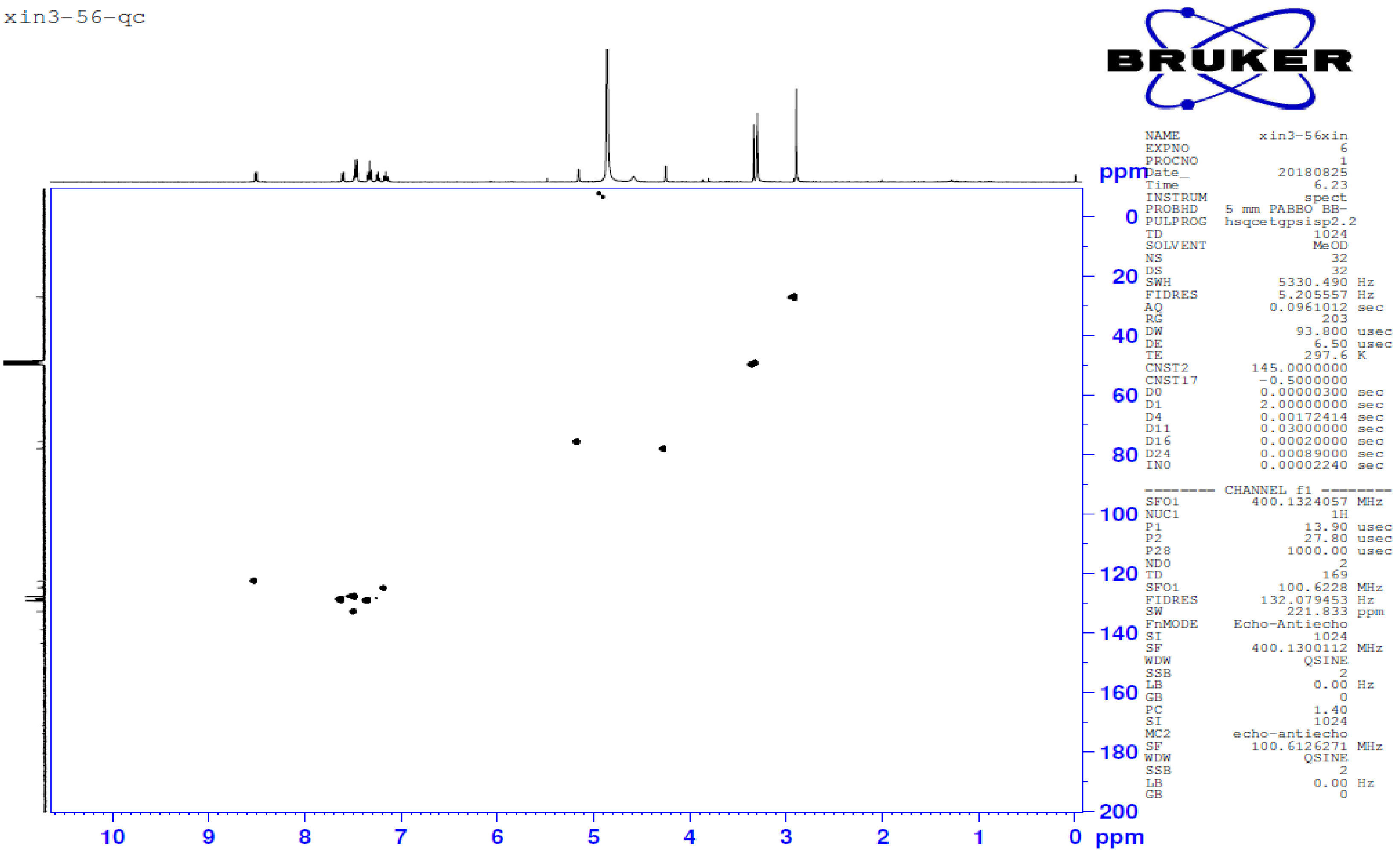

Figure S3. HSQC NMR spectrum of **1** in CD_3OD.

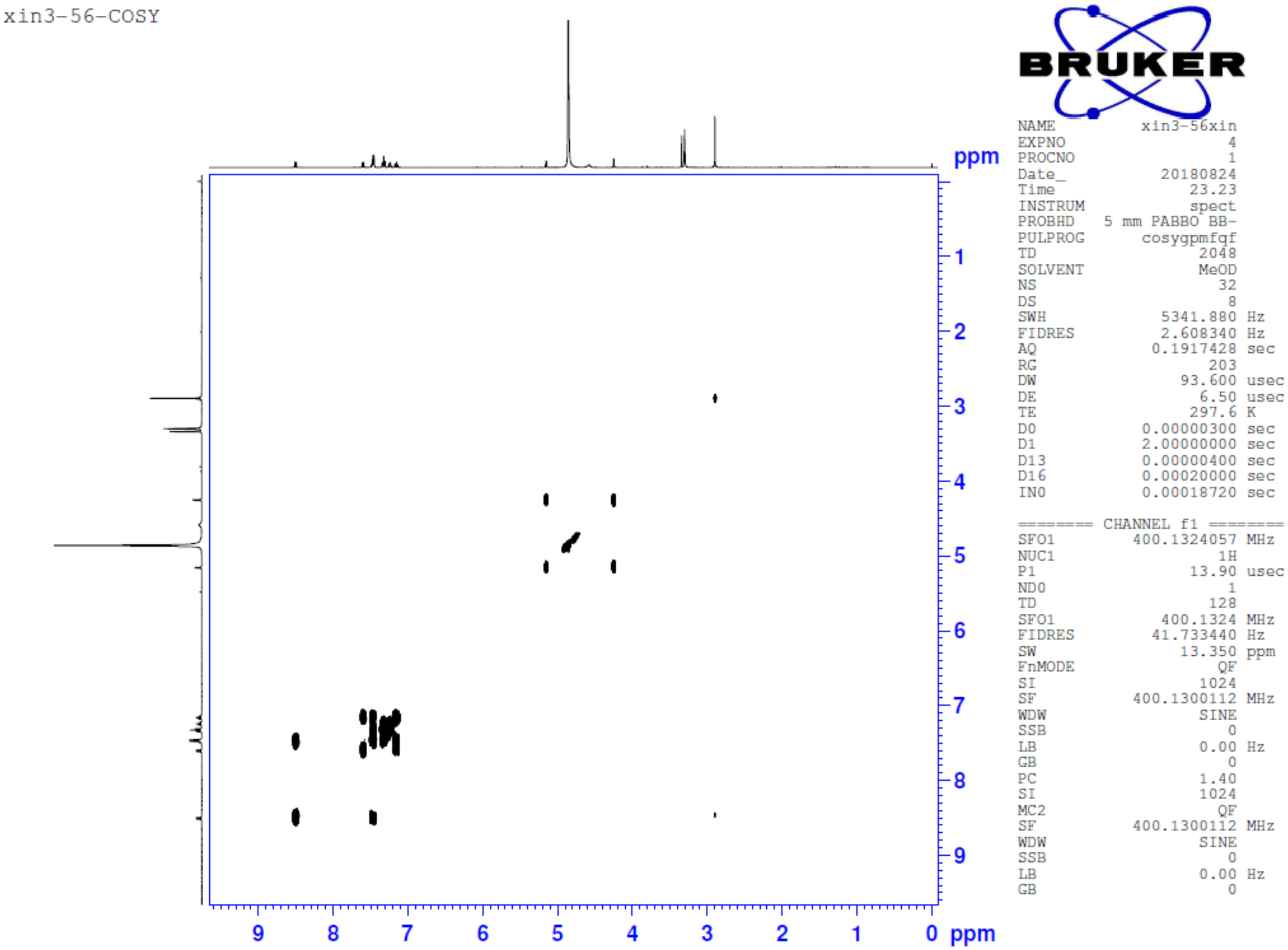

Figure S4. COSY NMR spectrum of **1** in CD_3OD.

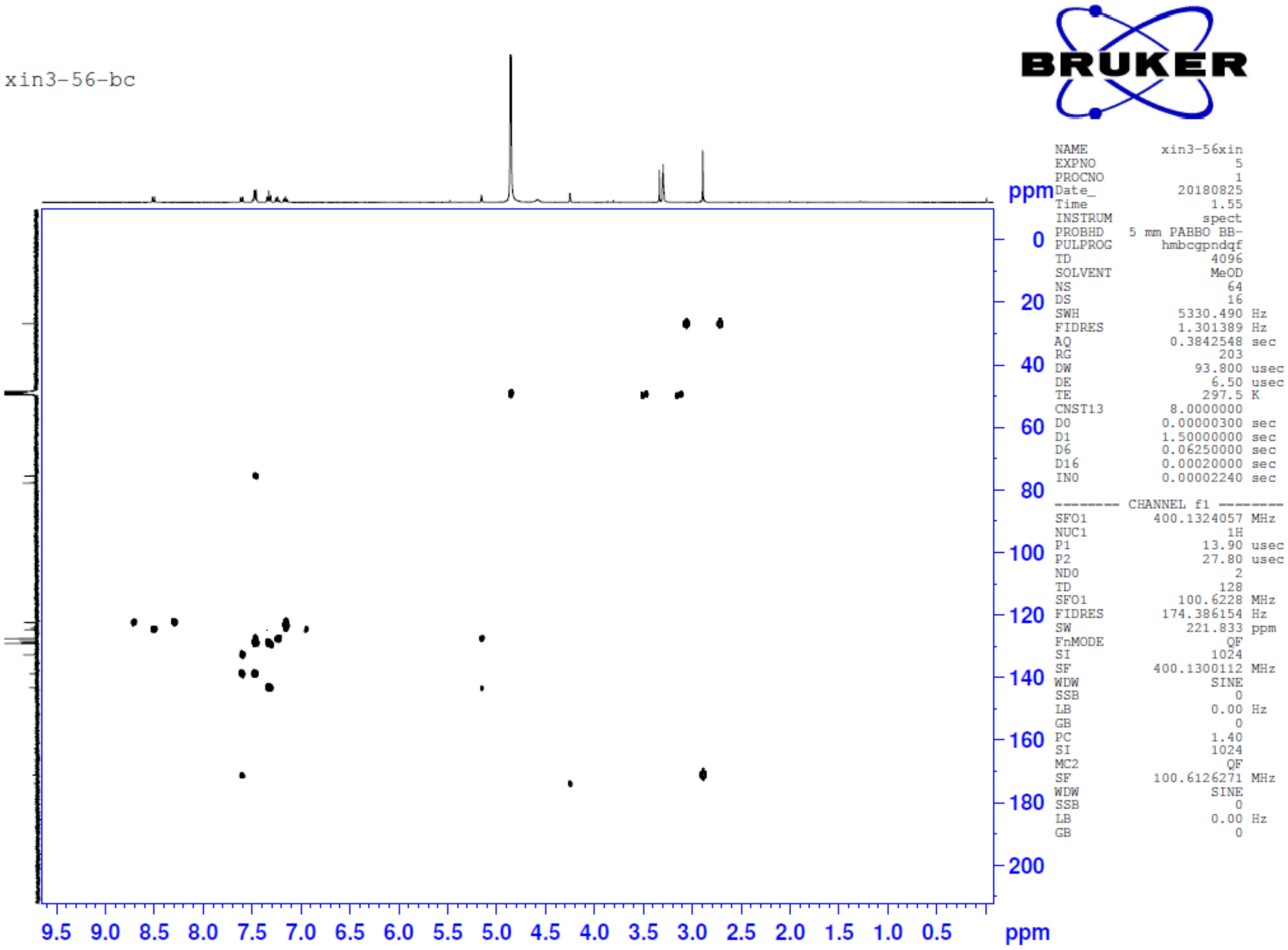

Figure S5. HMBC NMR spectrum of **1** in CD_3OD.

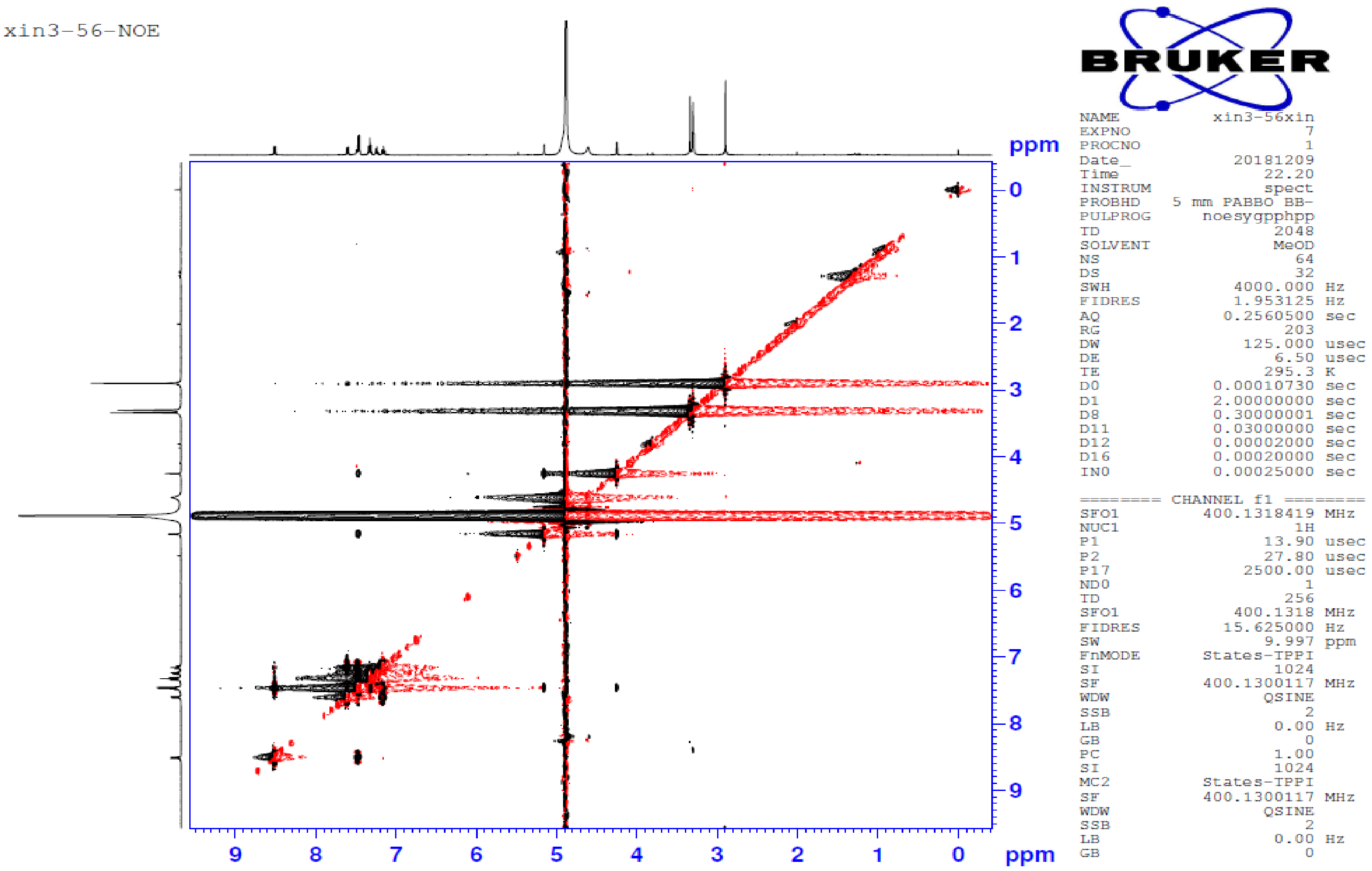

Figure S6. NOESY NMR spectrum of **1** in CD_3OD.

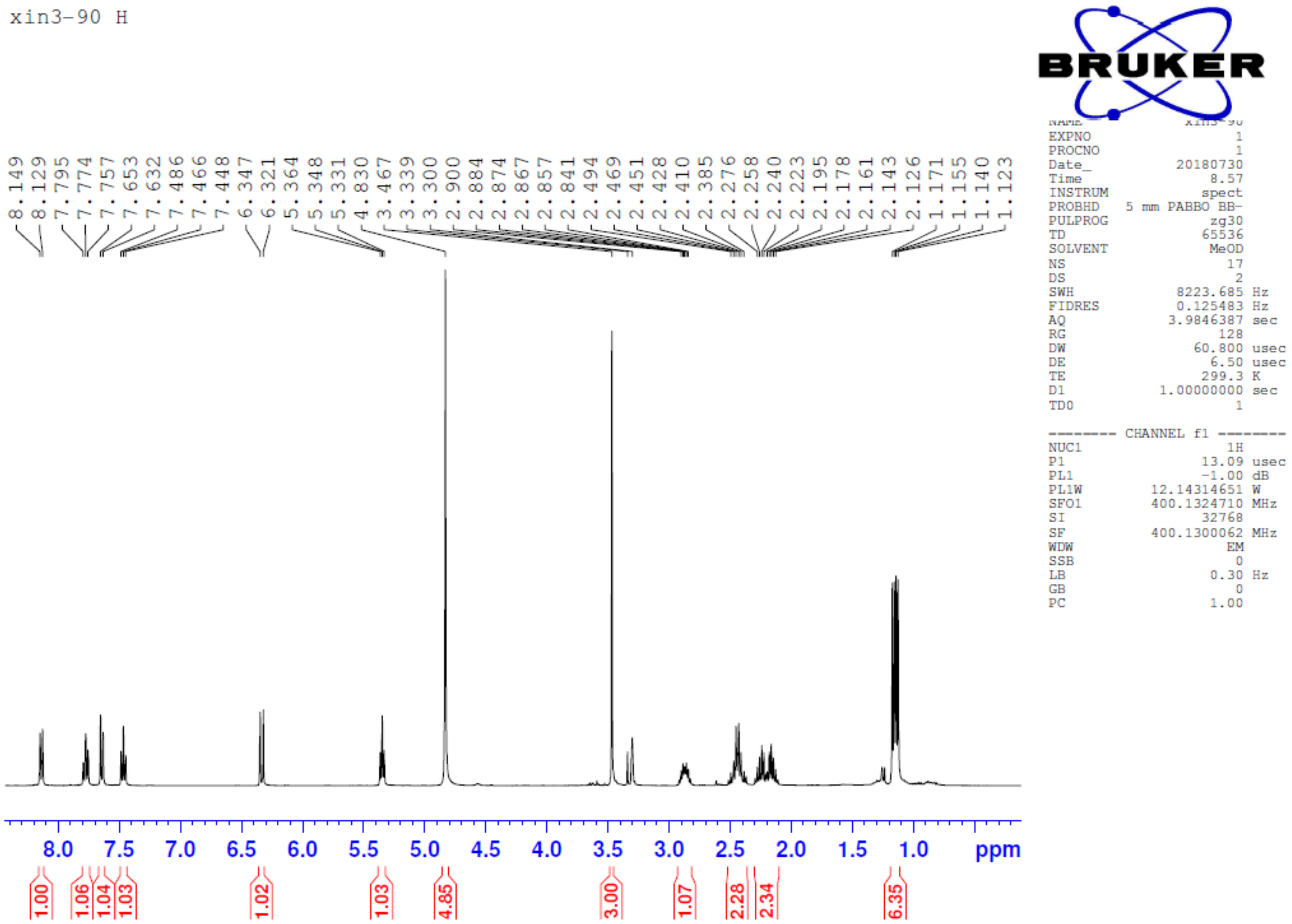

Figure S7. ^{1}H NMR spectrum of **2** in CD_3OD.

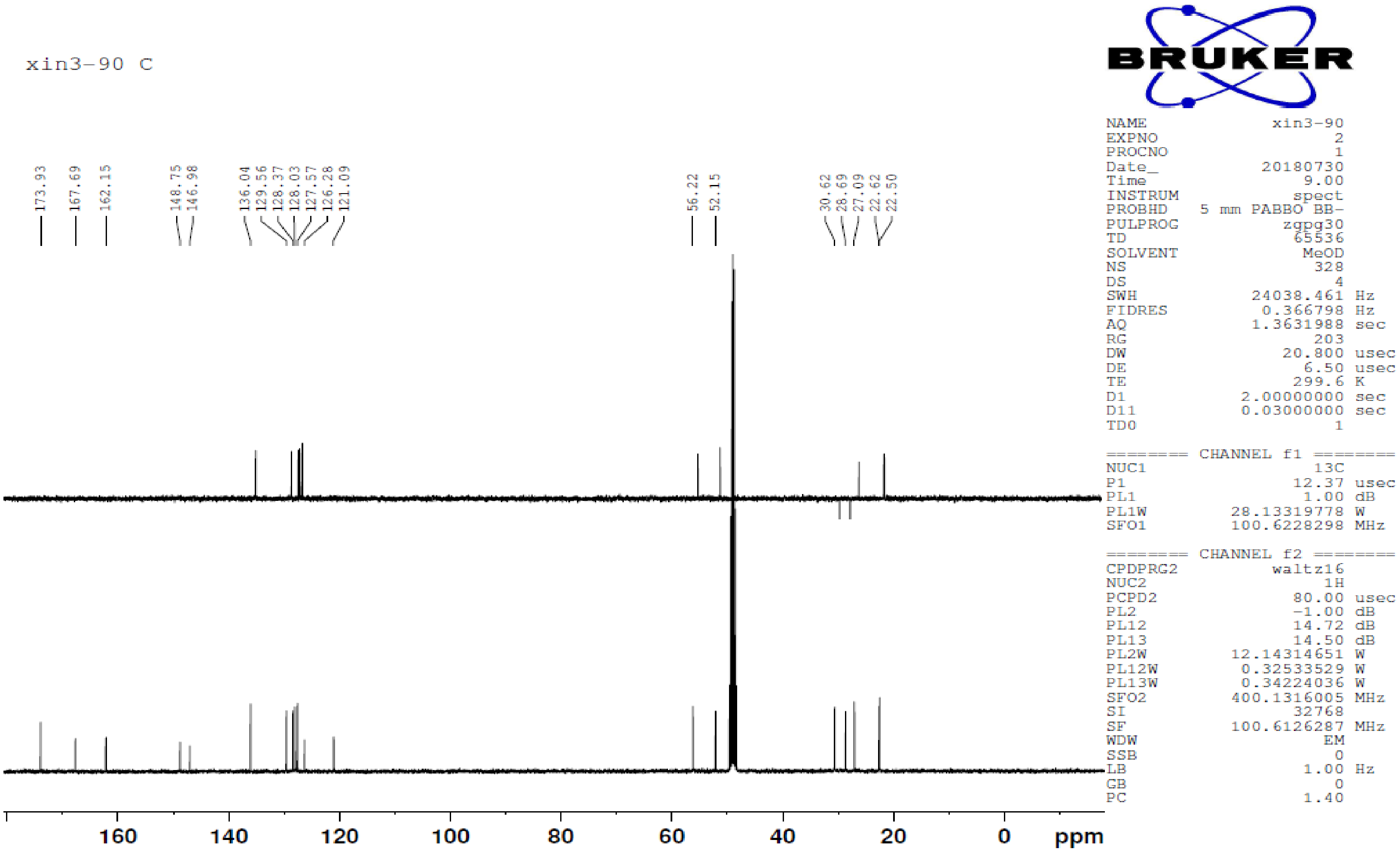

Figure S8. ^{13}C NMR spectrum of **2** in CD_3OD.

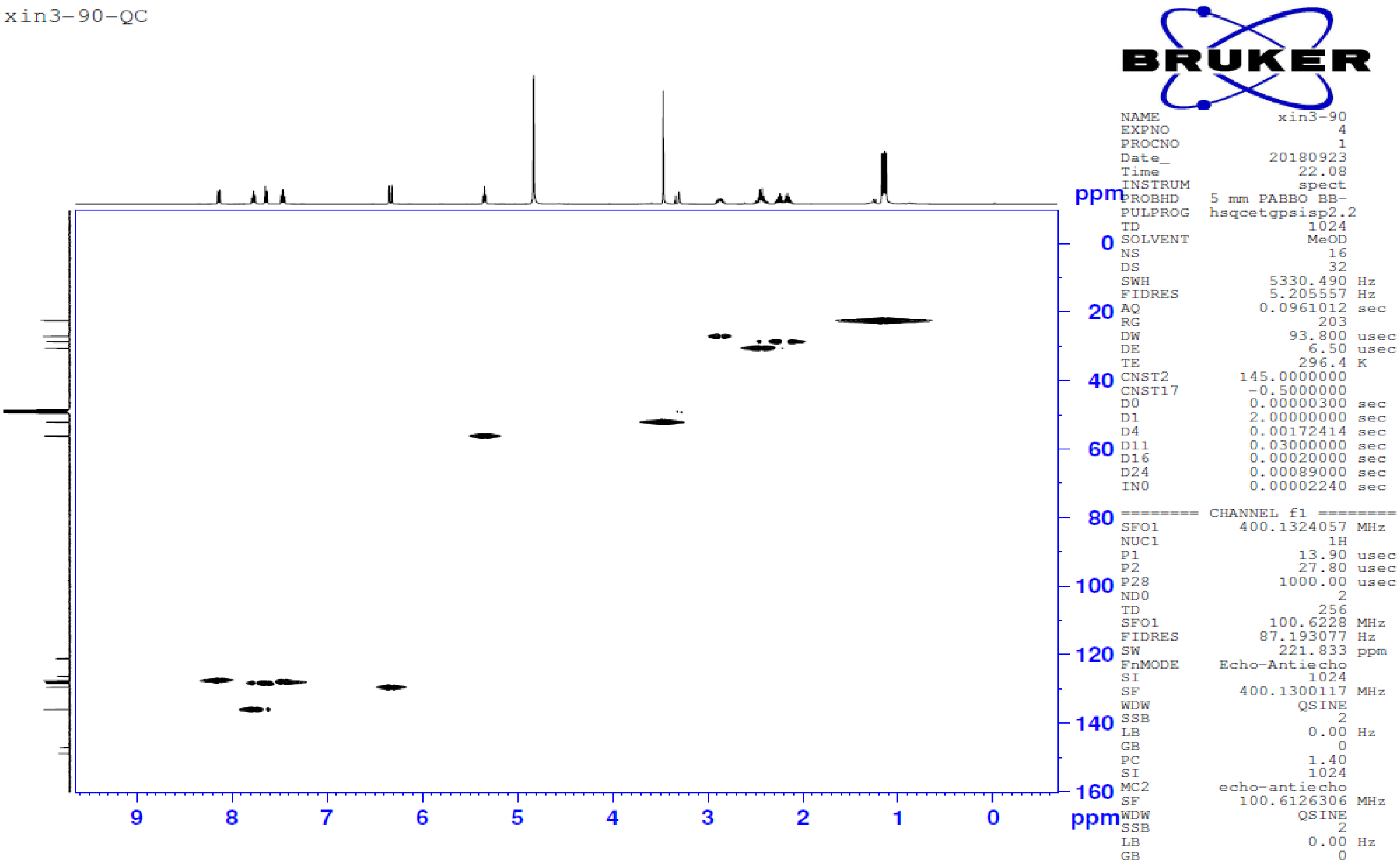

Figure S9. HSQC NMR spectrum of **2** in CD_3OD.

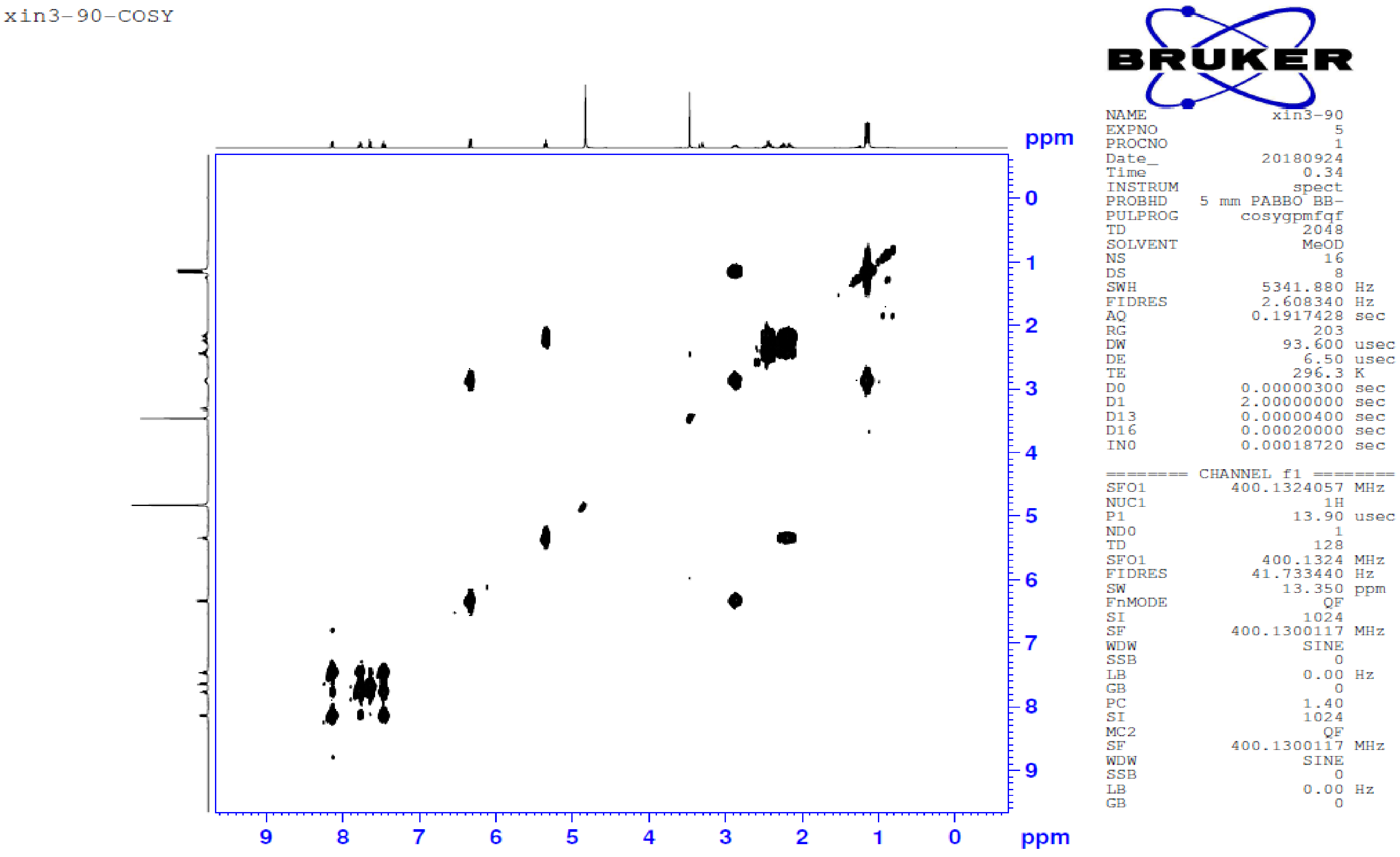

Figure S10. COSY NMR spectrum of **2** in CD_3OD.

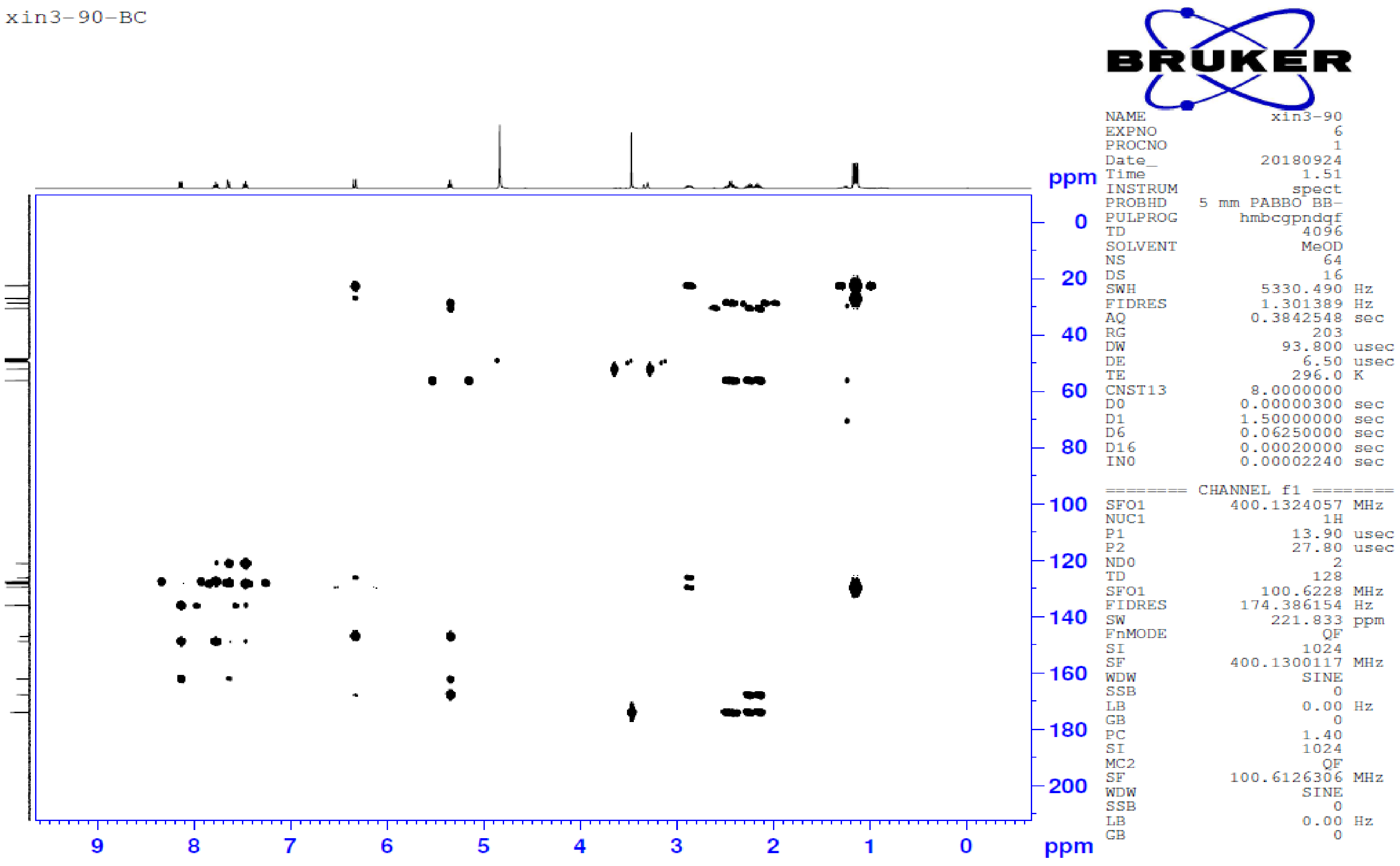

Figure S11. HMBC NMR spectrum of **2** in CD_3OD.

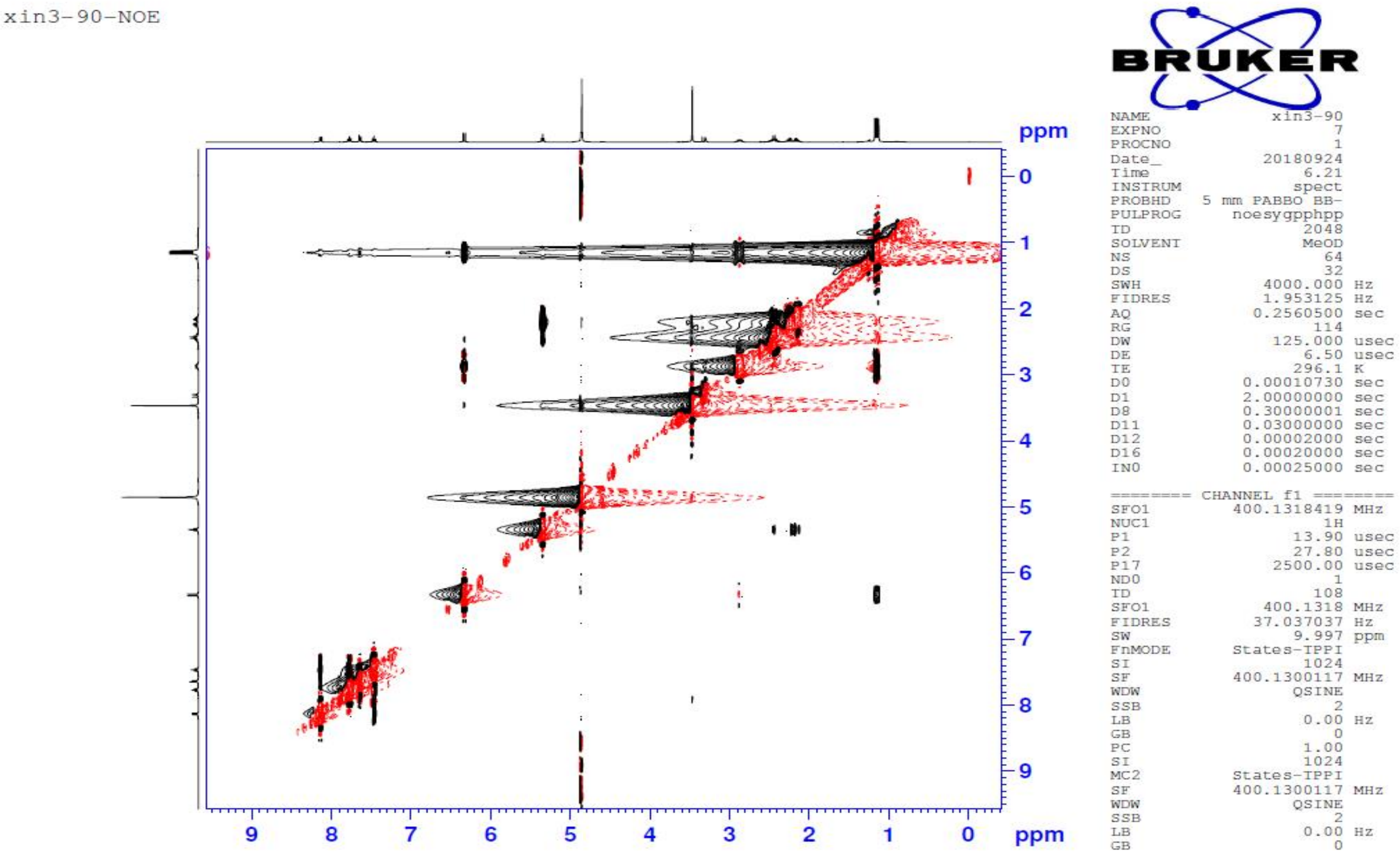

Figure S12. NOESY NMR spectrum of **2** in CD_3OD.

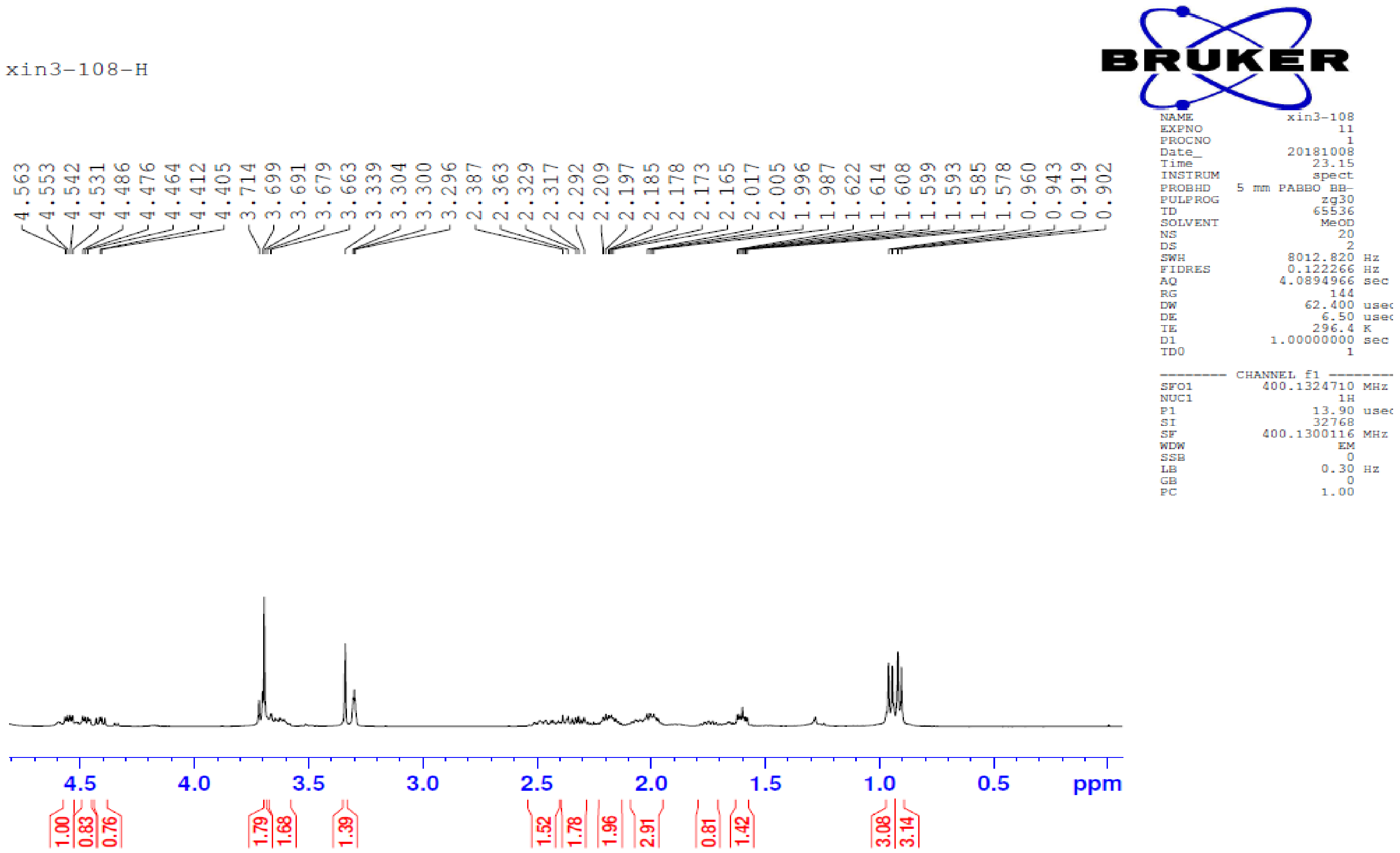

Figure S13. ^{1}H NMR spectrum of **3** in CD_3OD.

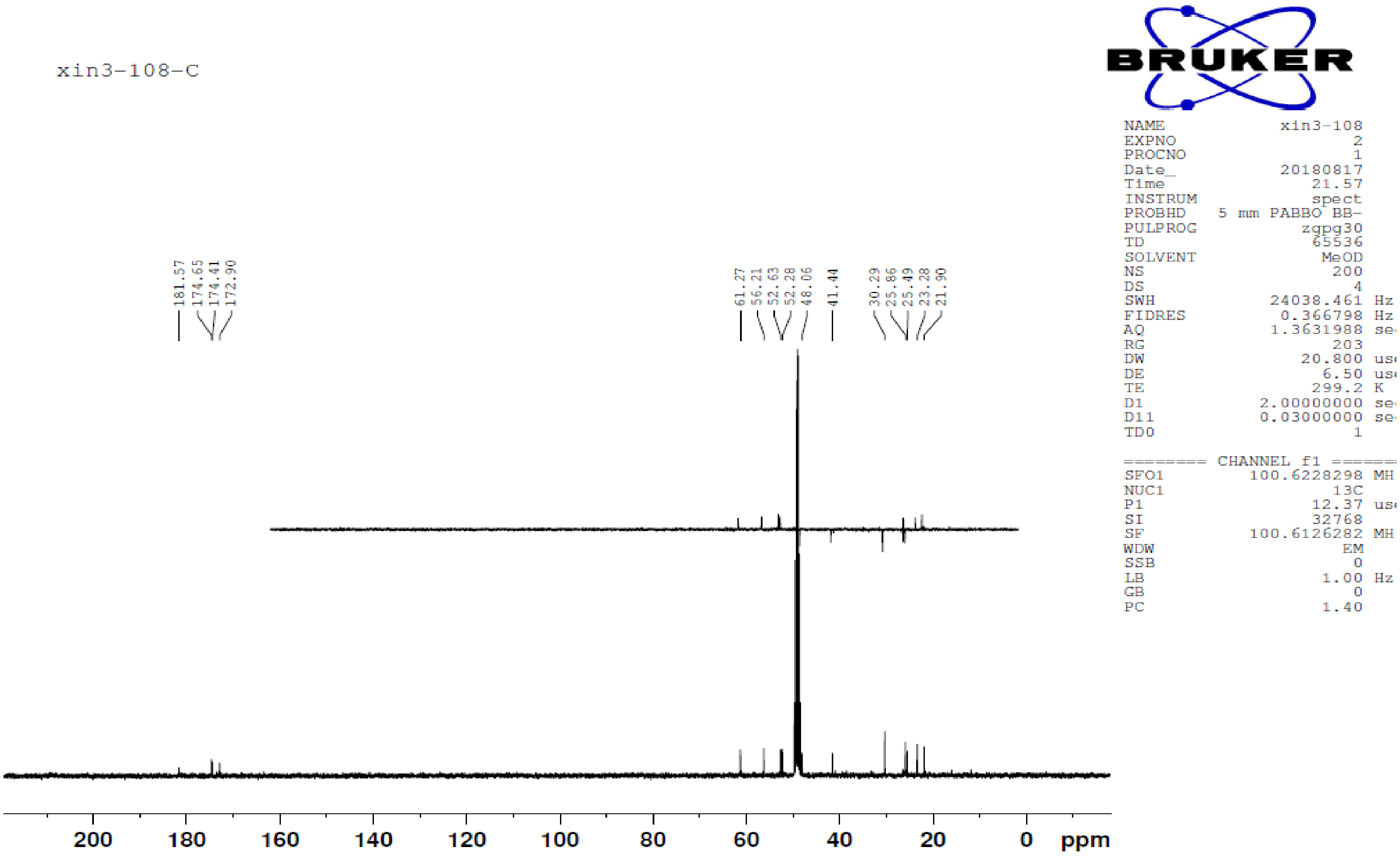

Figure S14. ^{13}C NMR spectrum of **3** in CD_3OD.

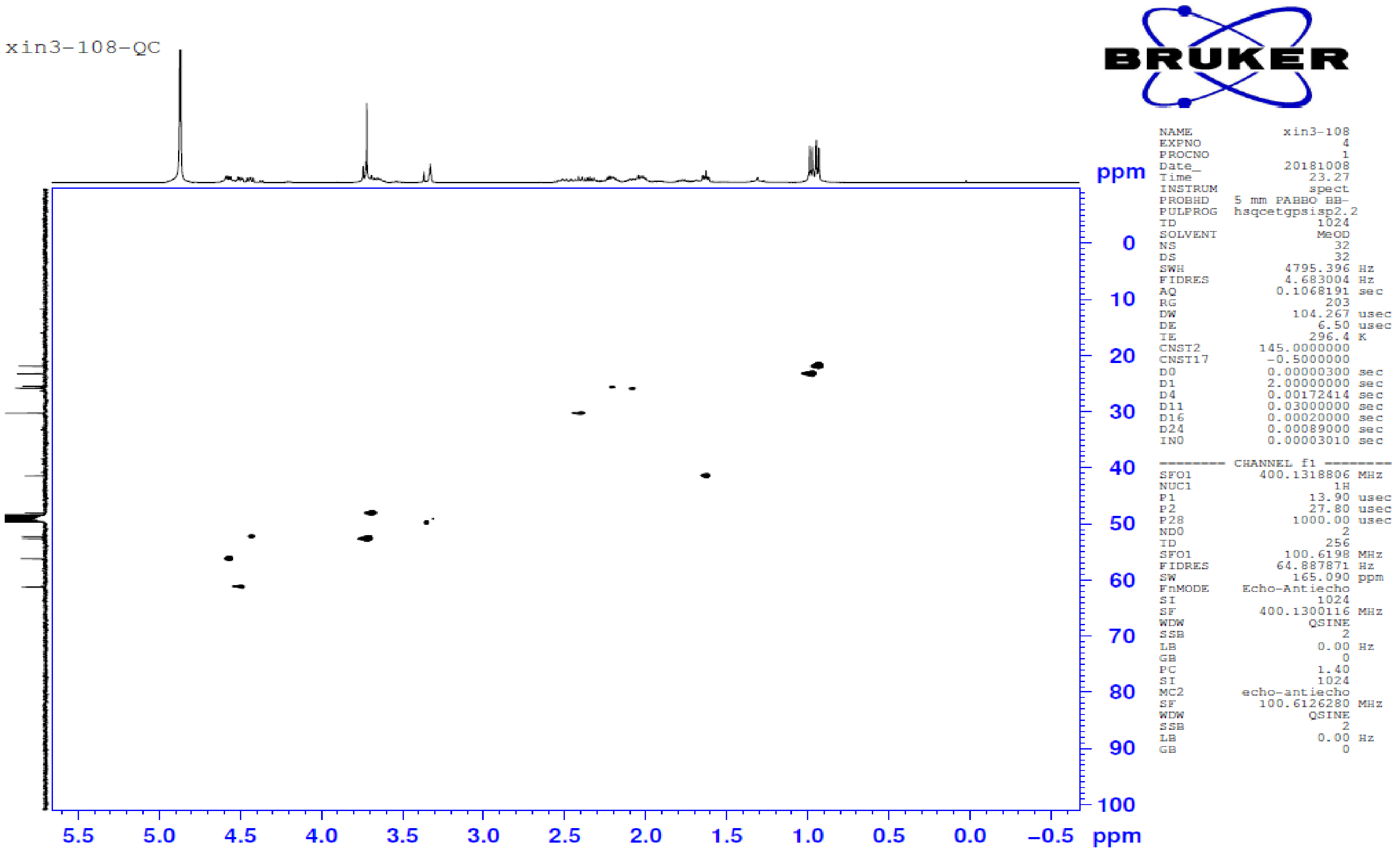

Figure S15. HSQC NMR spectrum of **3** in CD_3OD.

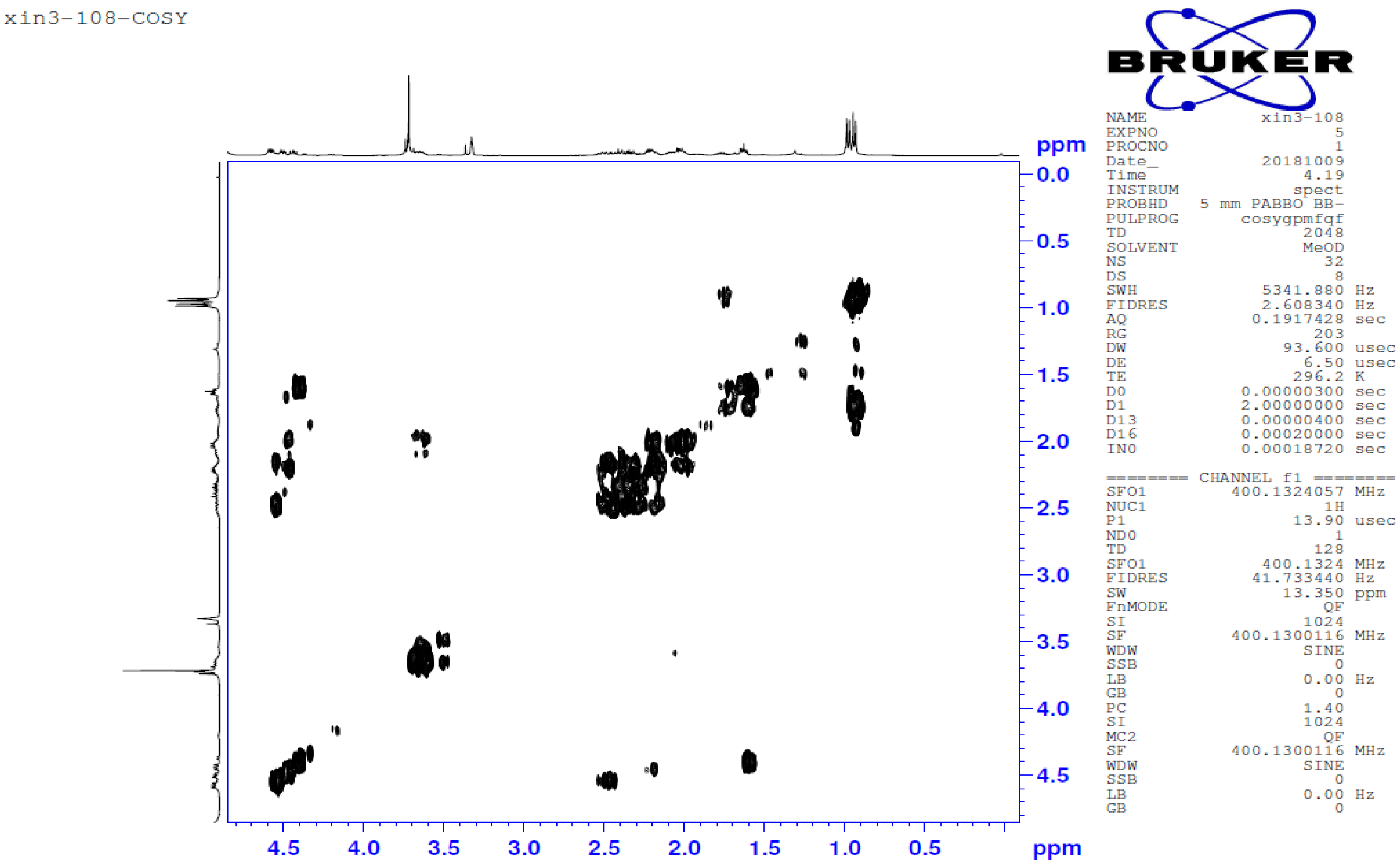

Figure S16. COSY NMR spectrum of **3** in CD_3OD.

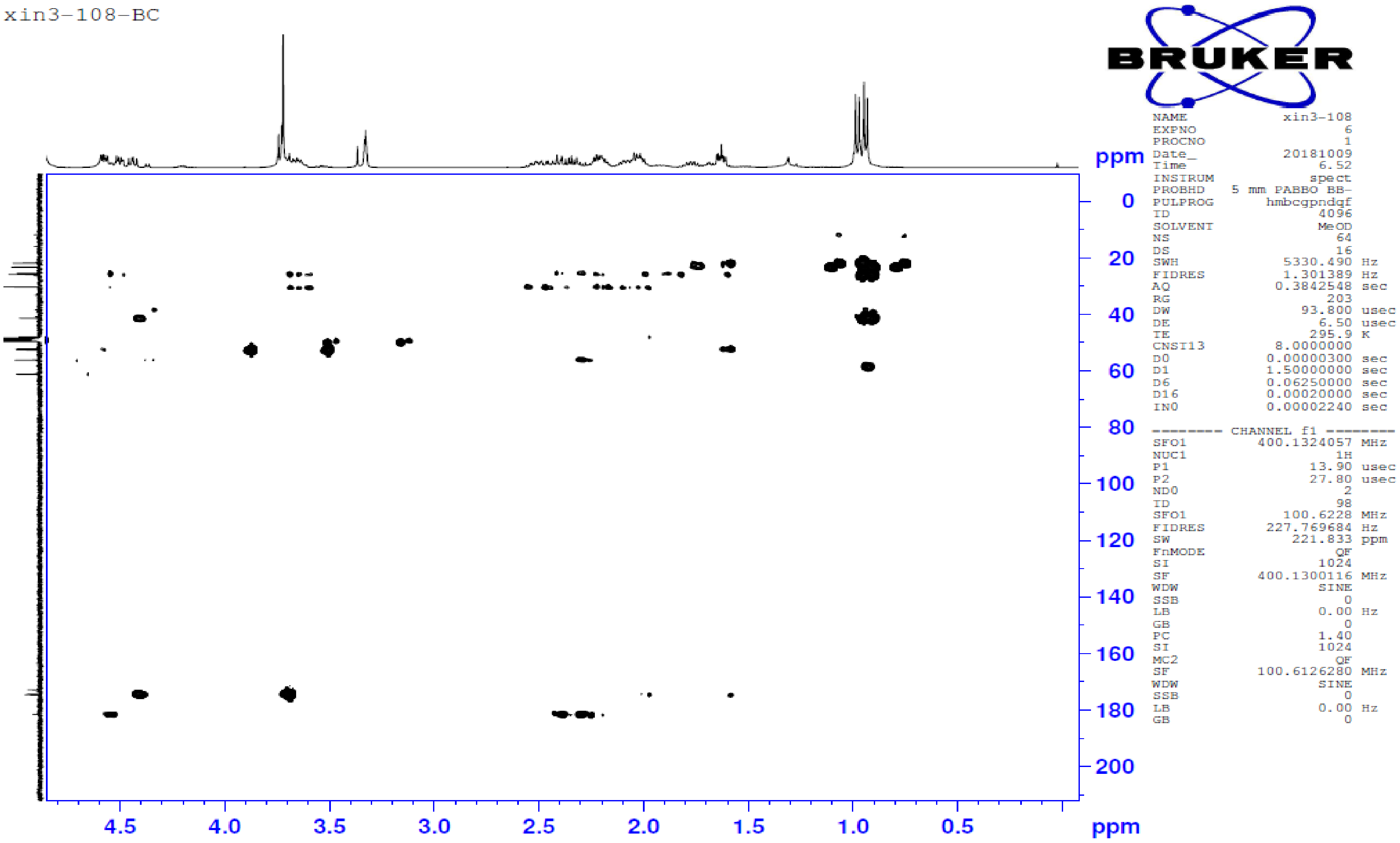

Figure S17. HMBC NMR spectrum of **3** in CD_3OD.

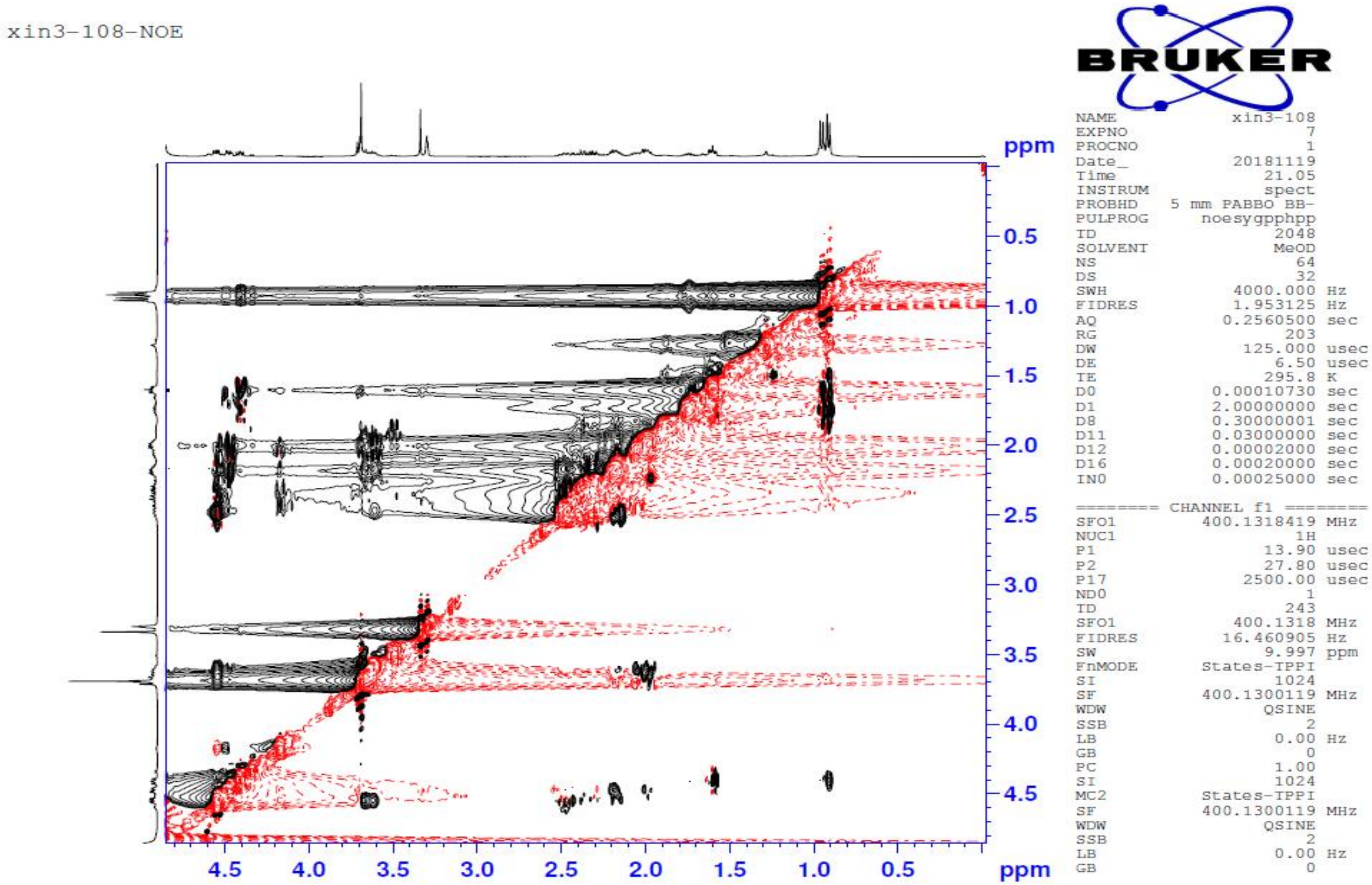

Figure S18. NOESY NMR spectrum of **3** in CD_3OD.

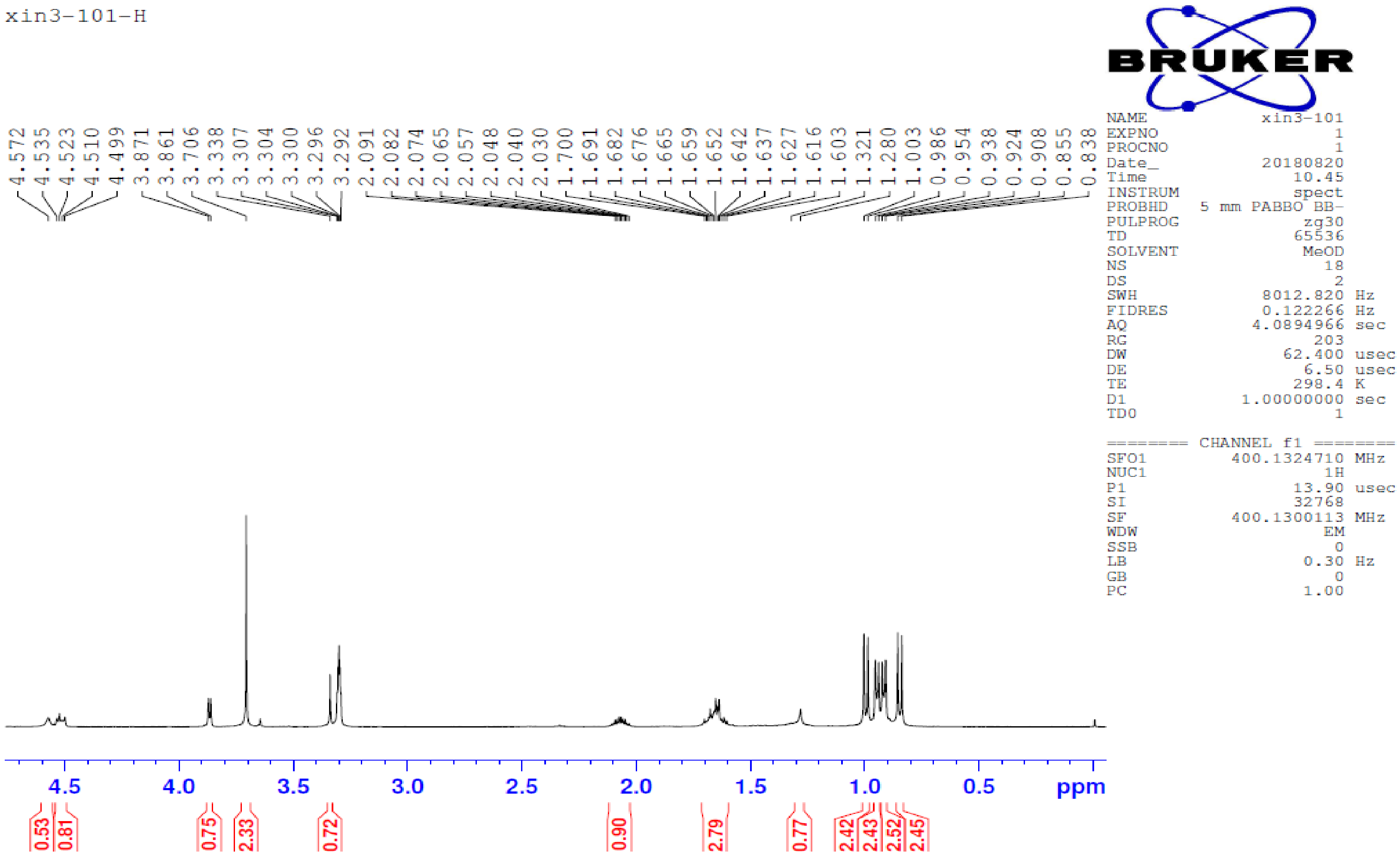

Figure S19. ^{1}H NMR spectrum of **4** in CD_3OD.

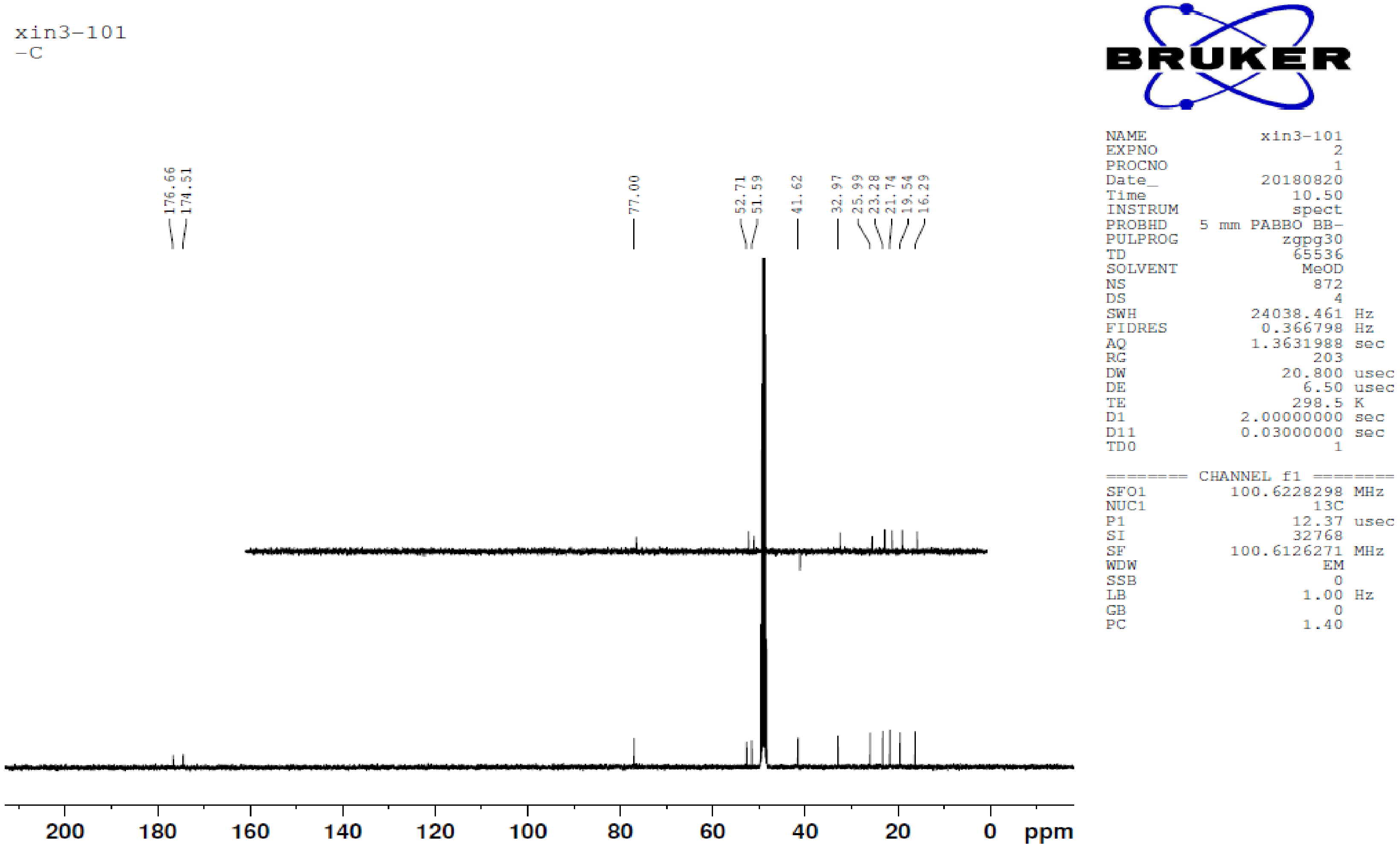

Figure S20. ^{13}C NMR spectrum of **4** in CD_3OD.

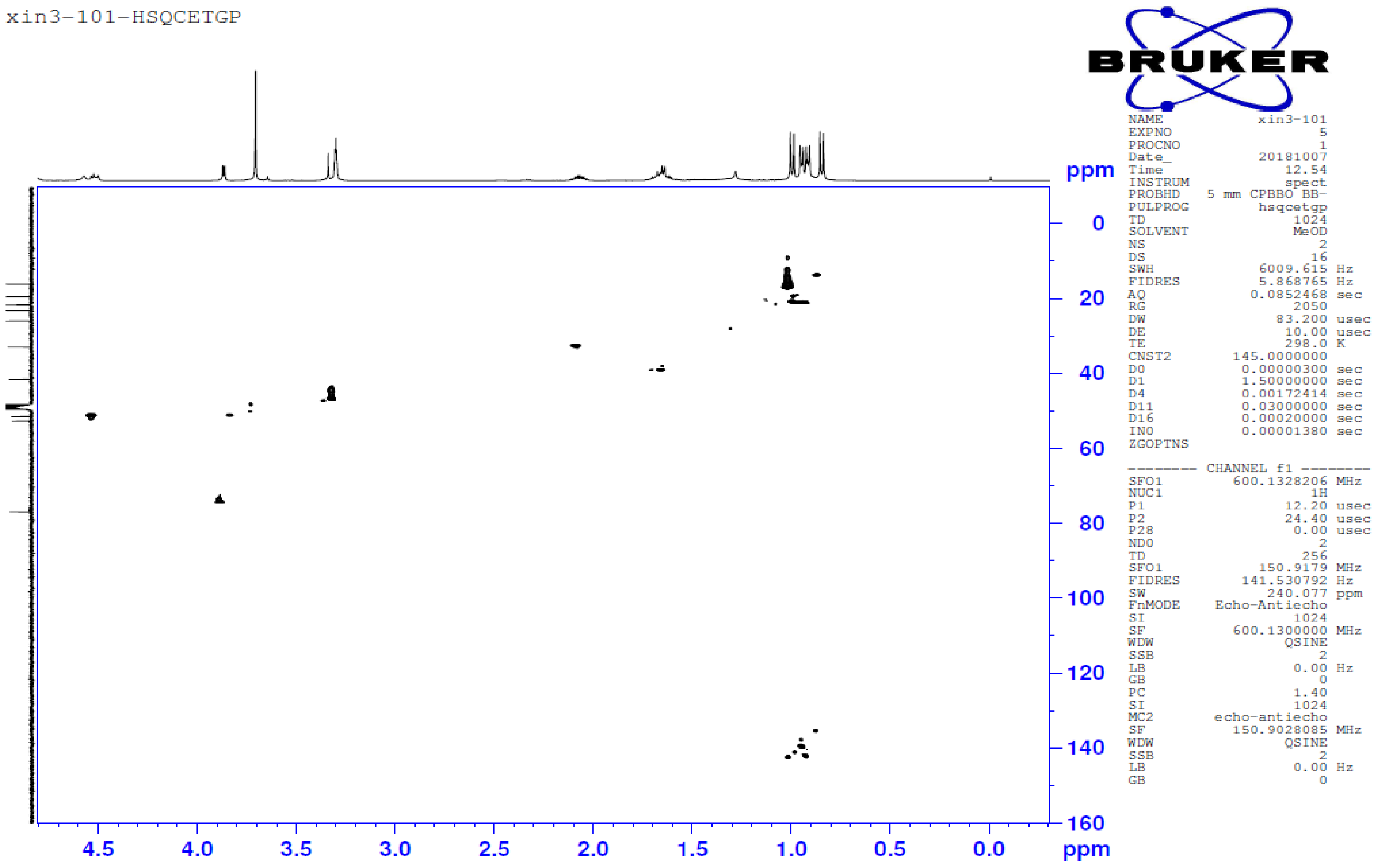

Figure S21. HSQC NMR spectrum of **4** in CD_3OD.

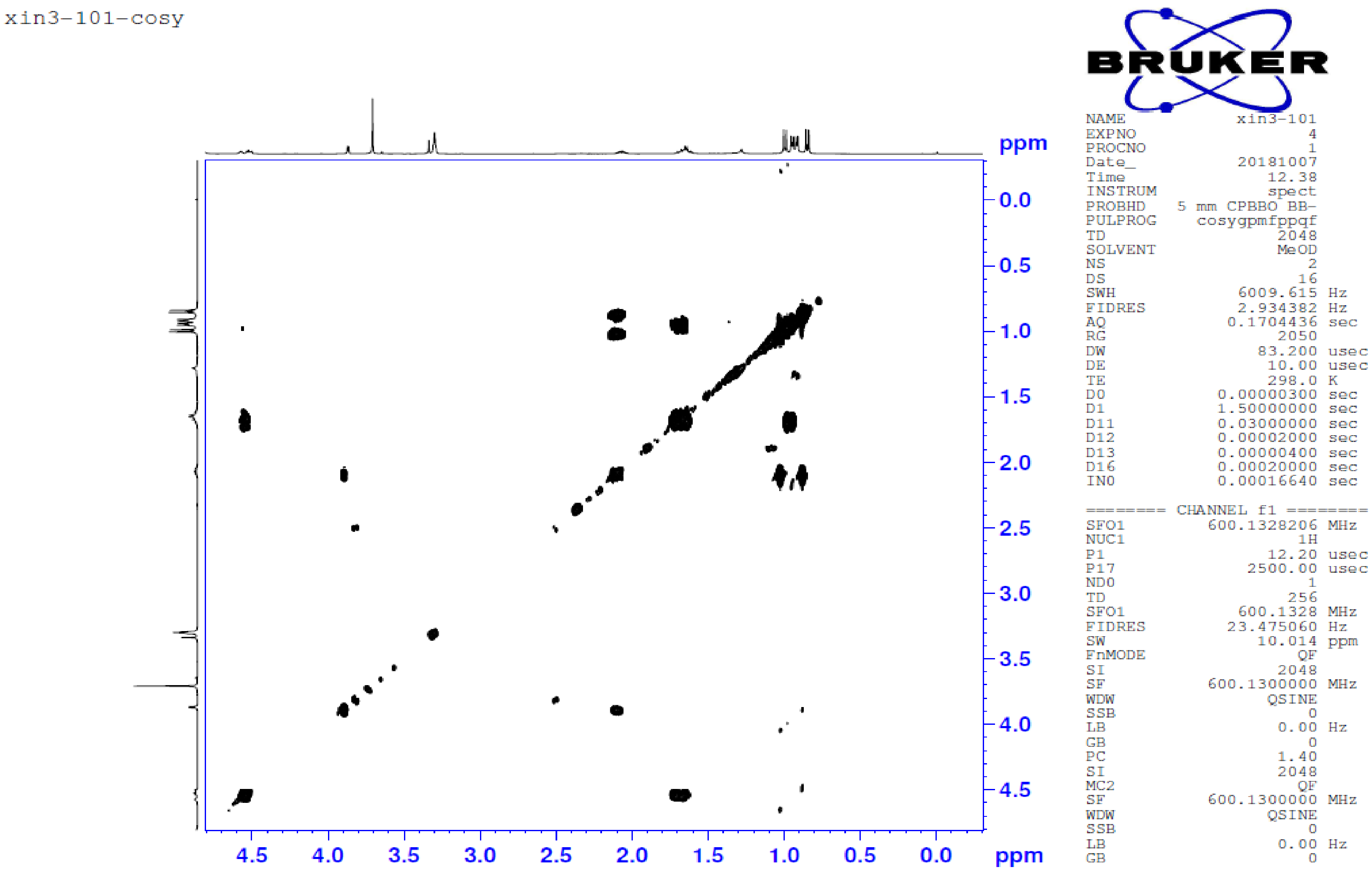

Figure S22. COSY NMR spectrum of **4** in CD_3OD.

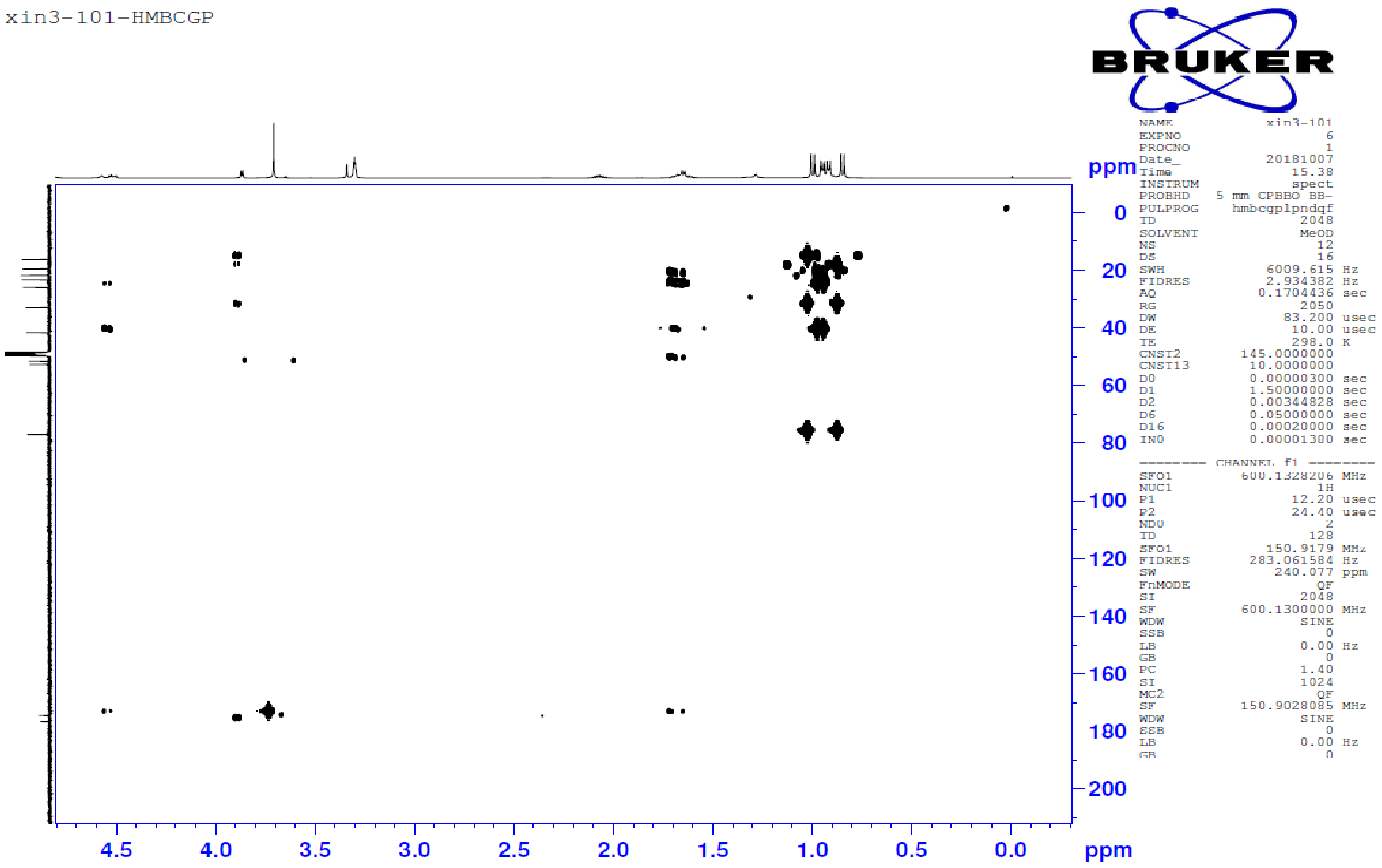

Figure S23. HMBC NMR spectrum of **4** in CD_3OD.

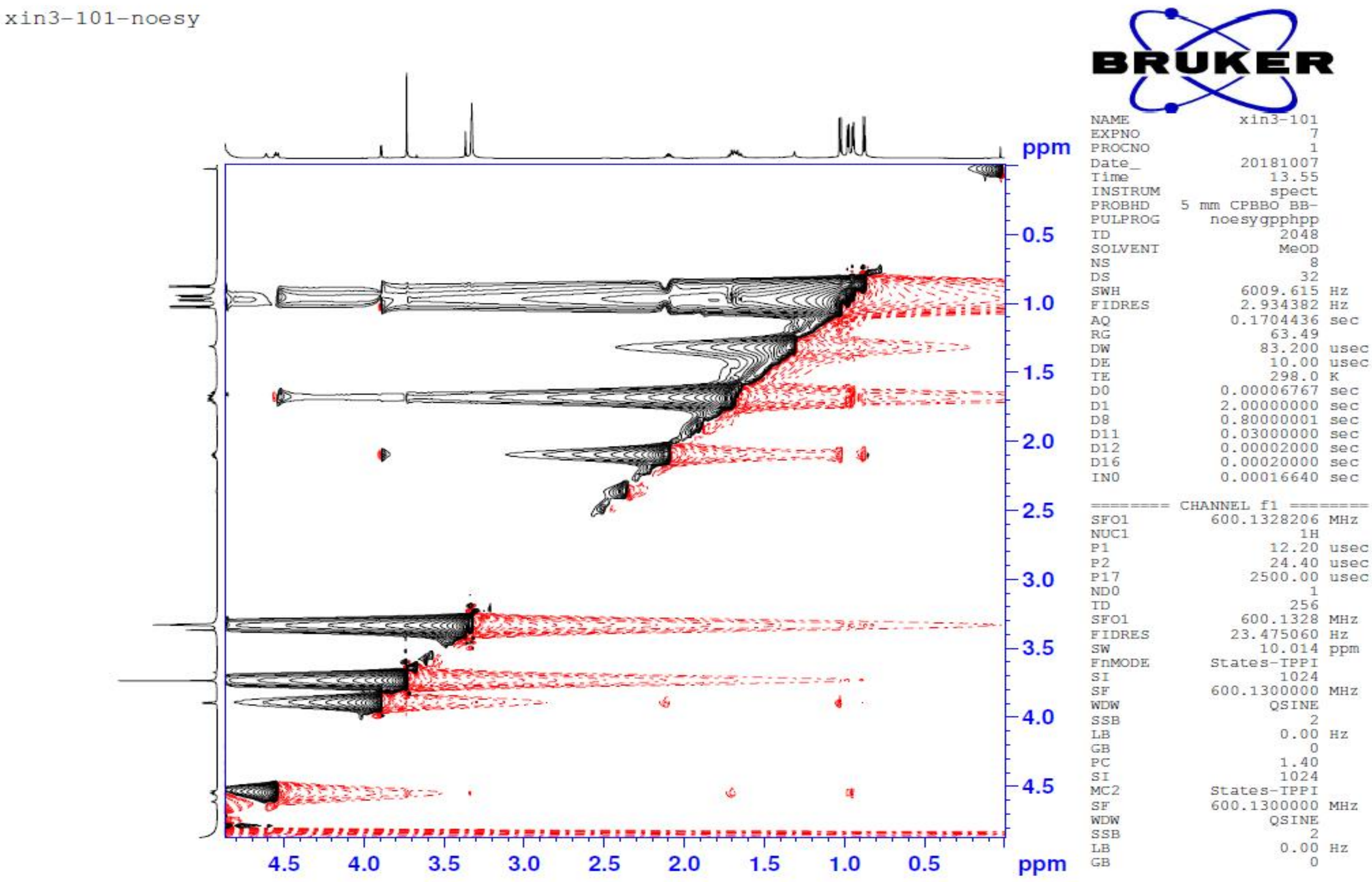

Figure S24. NOESY NMR spectrum of **4** in CD_3OD.

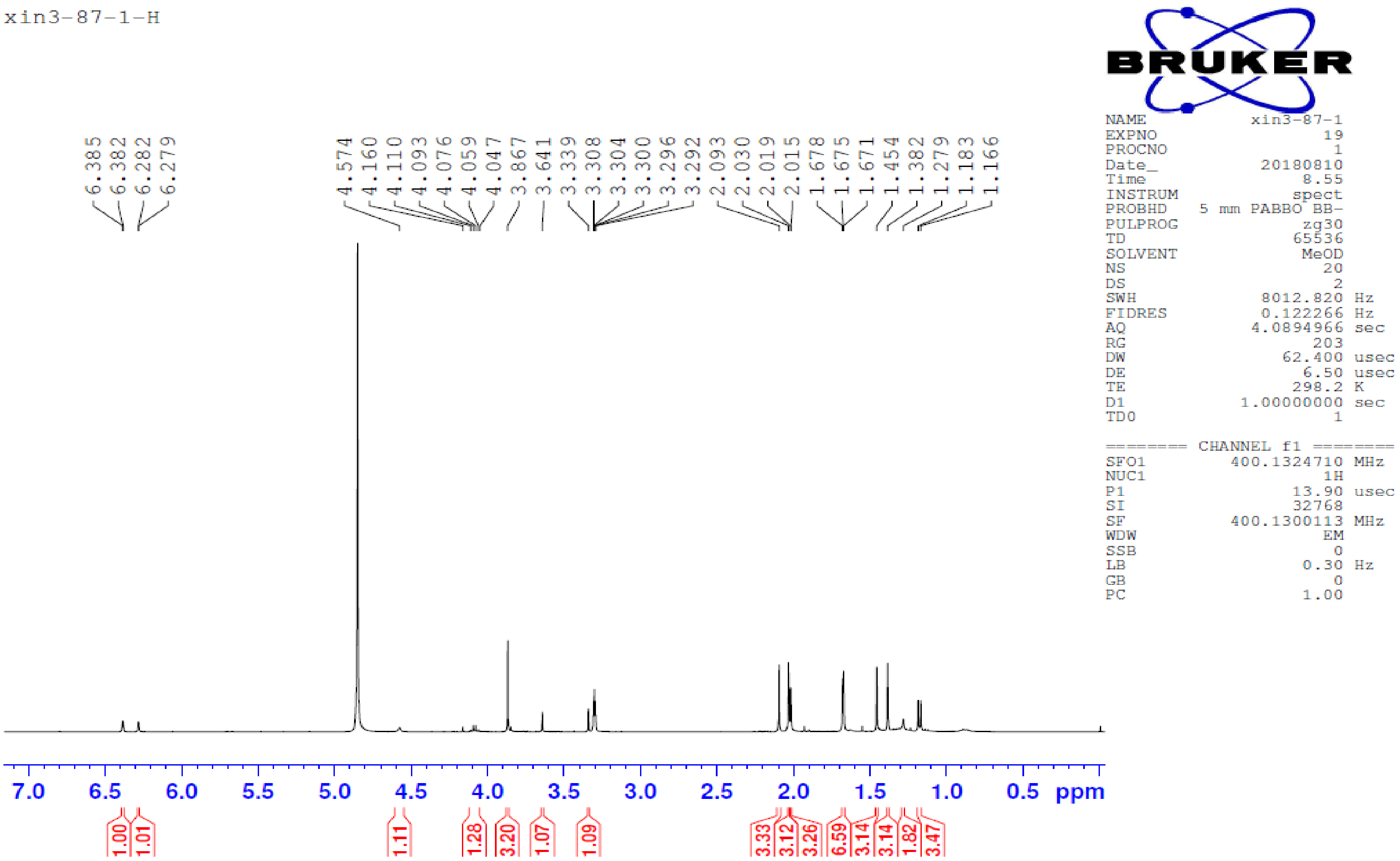

Figure S25. ^{1}H NMR spectrum of **5** in CD_3OD.

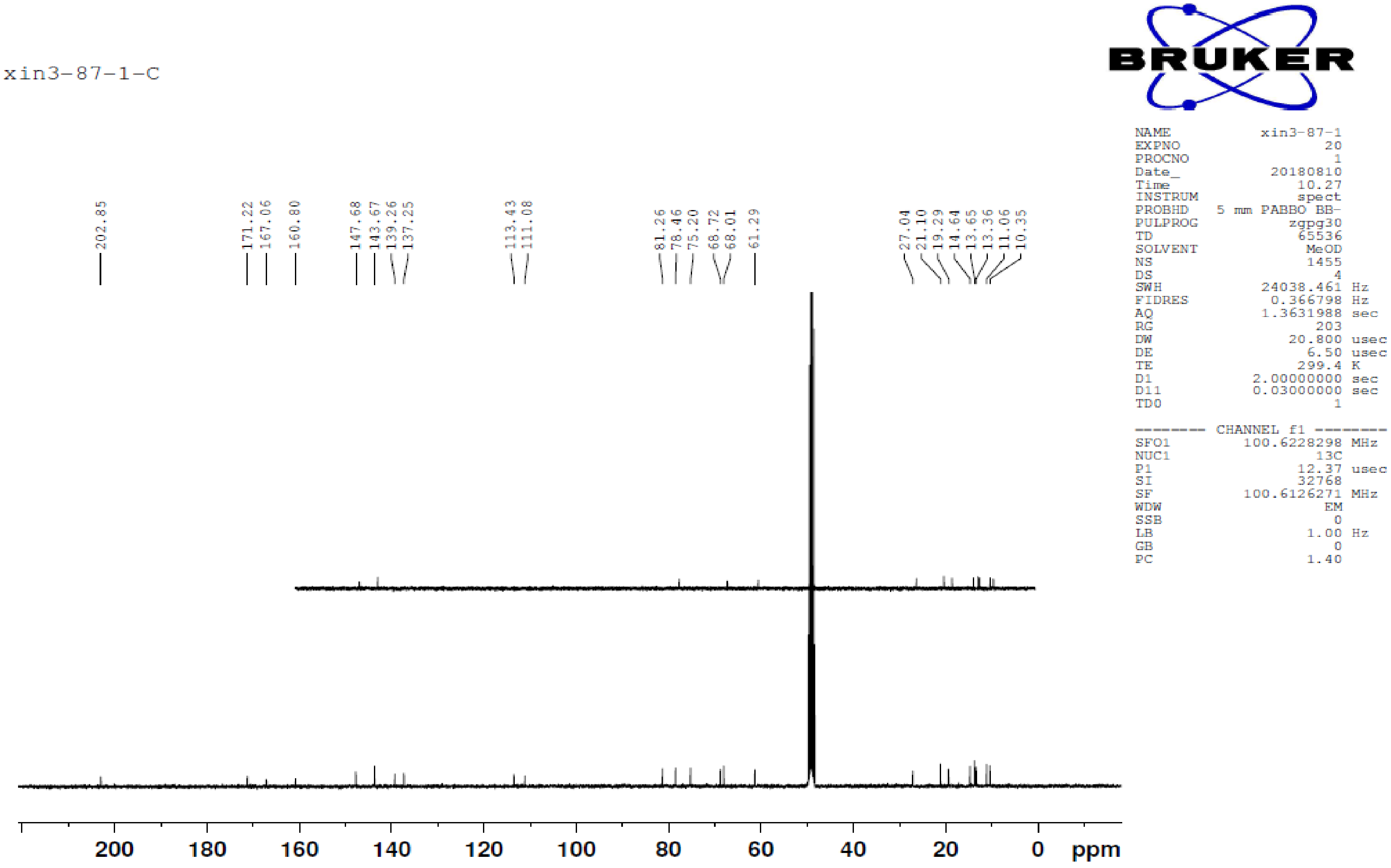

Figure S26. ^{13}C NMR spectrum of **5** in CD_3OD.

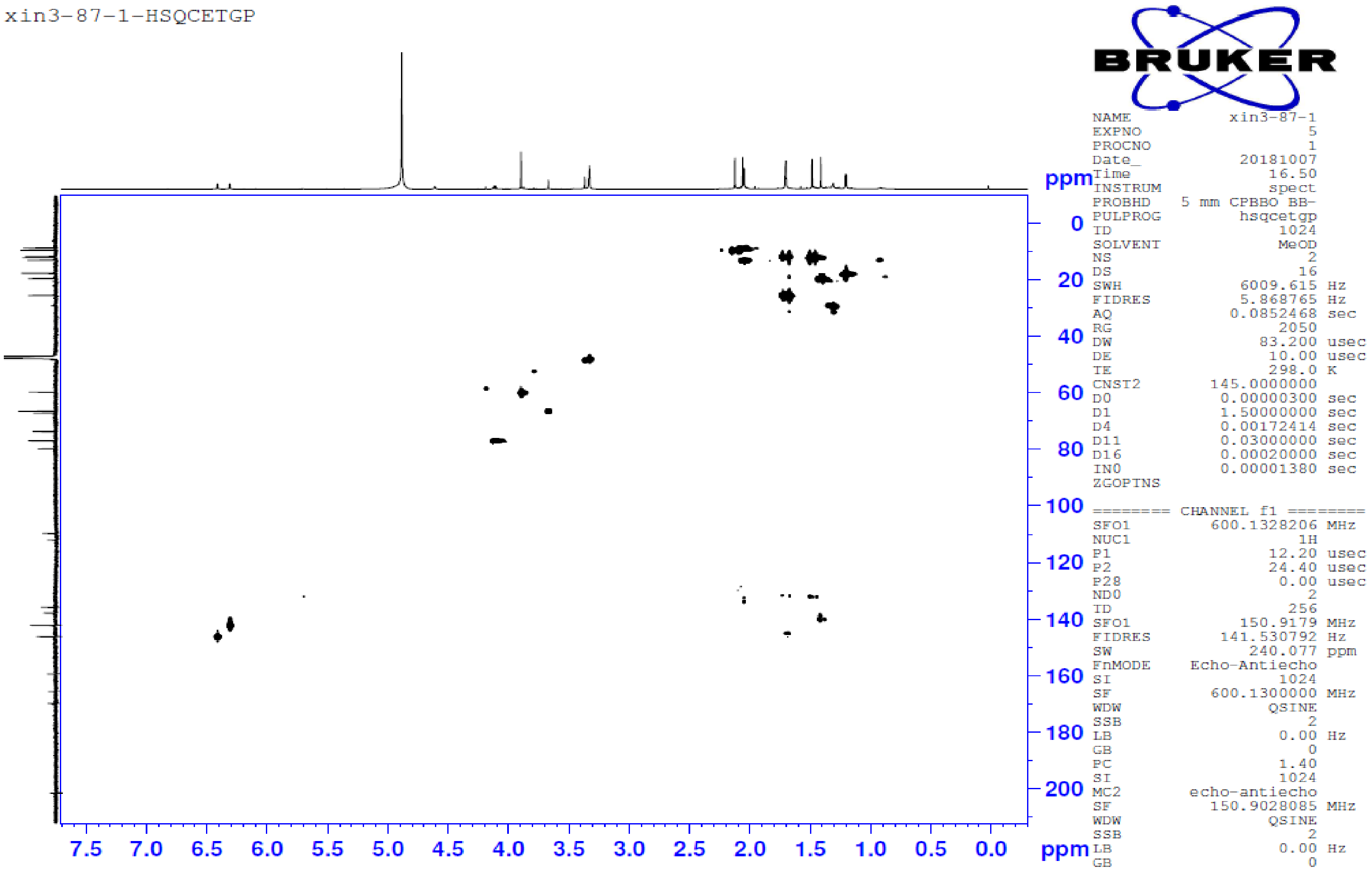

Figure S27. HSQC NMR spectrum of **5** in CD_3OD.

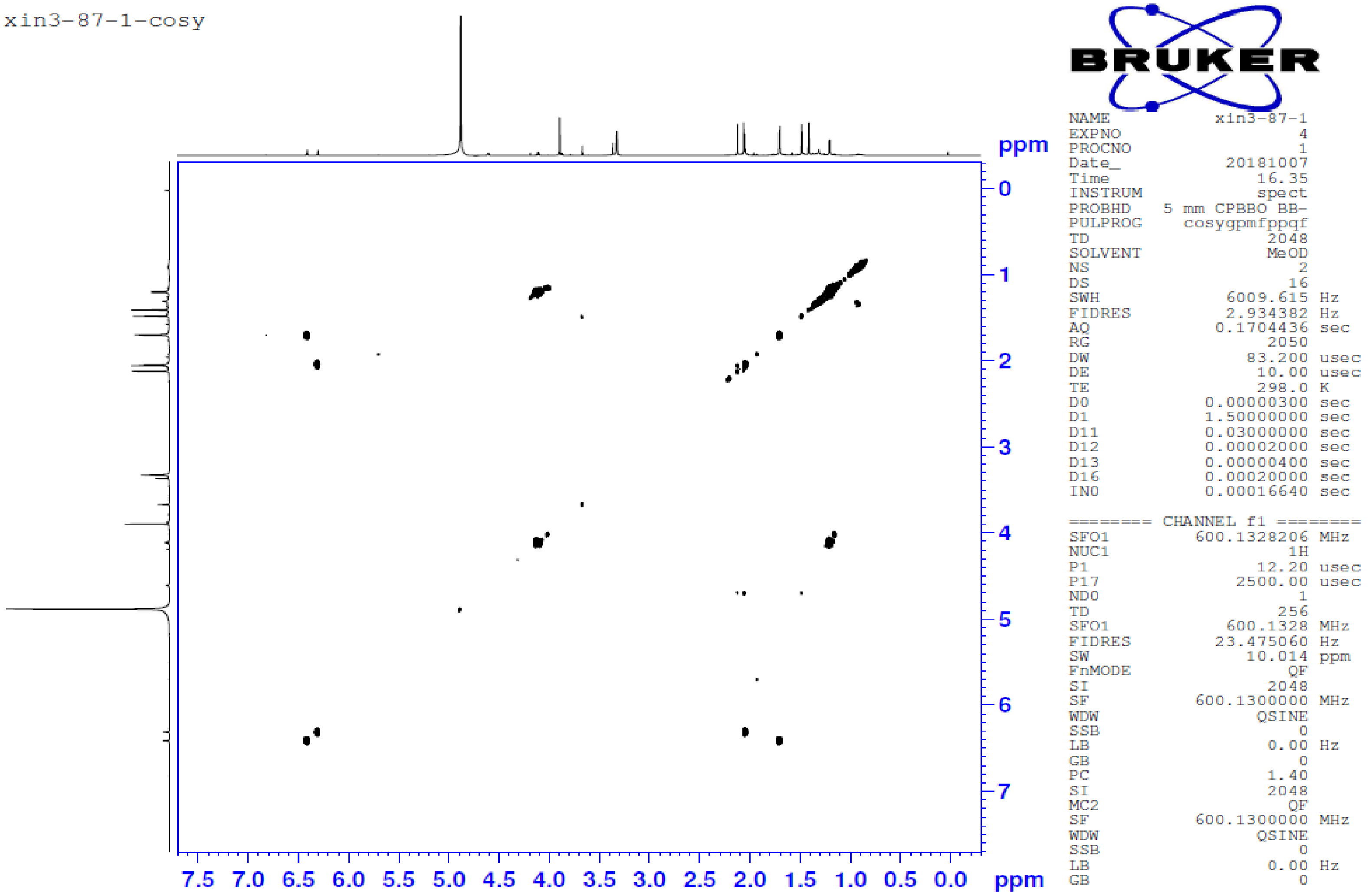

Figure S28. COSY NMR spectrum of **5** in CD_3OD.

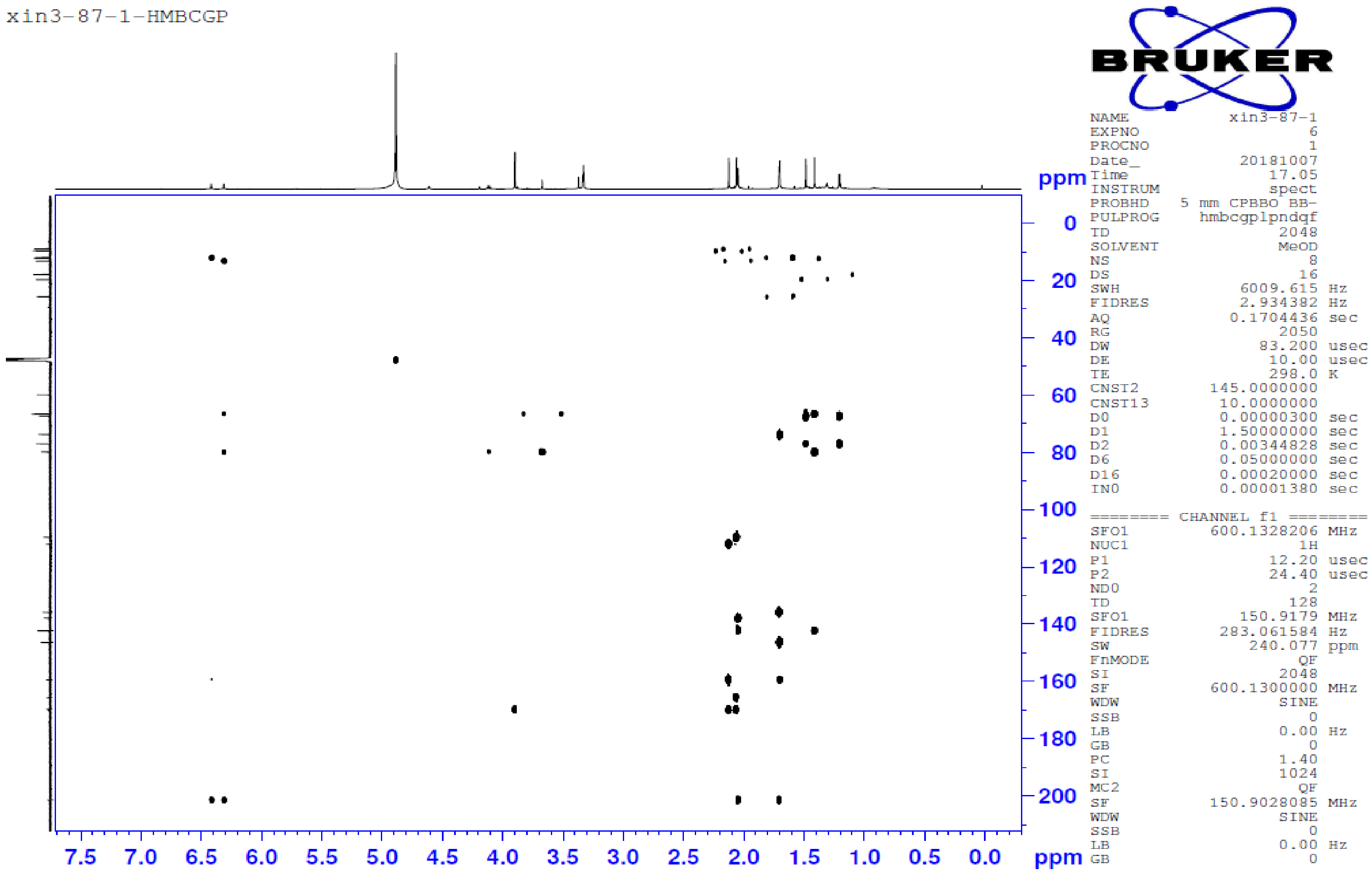

Figure S29. HMBC NMR spectrum of **5** in CD_3OD.

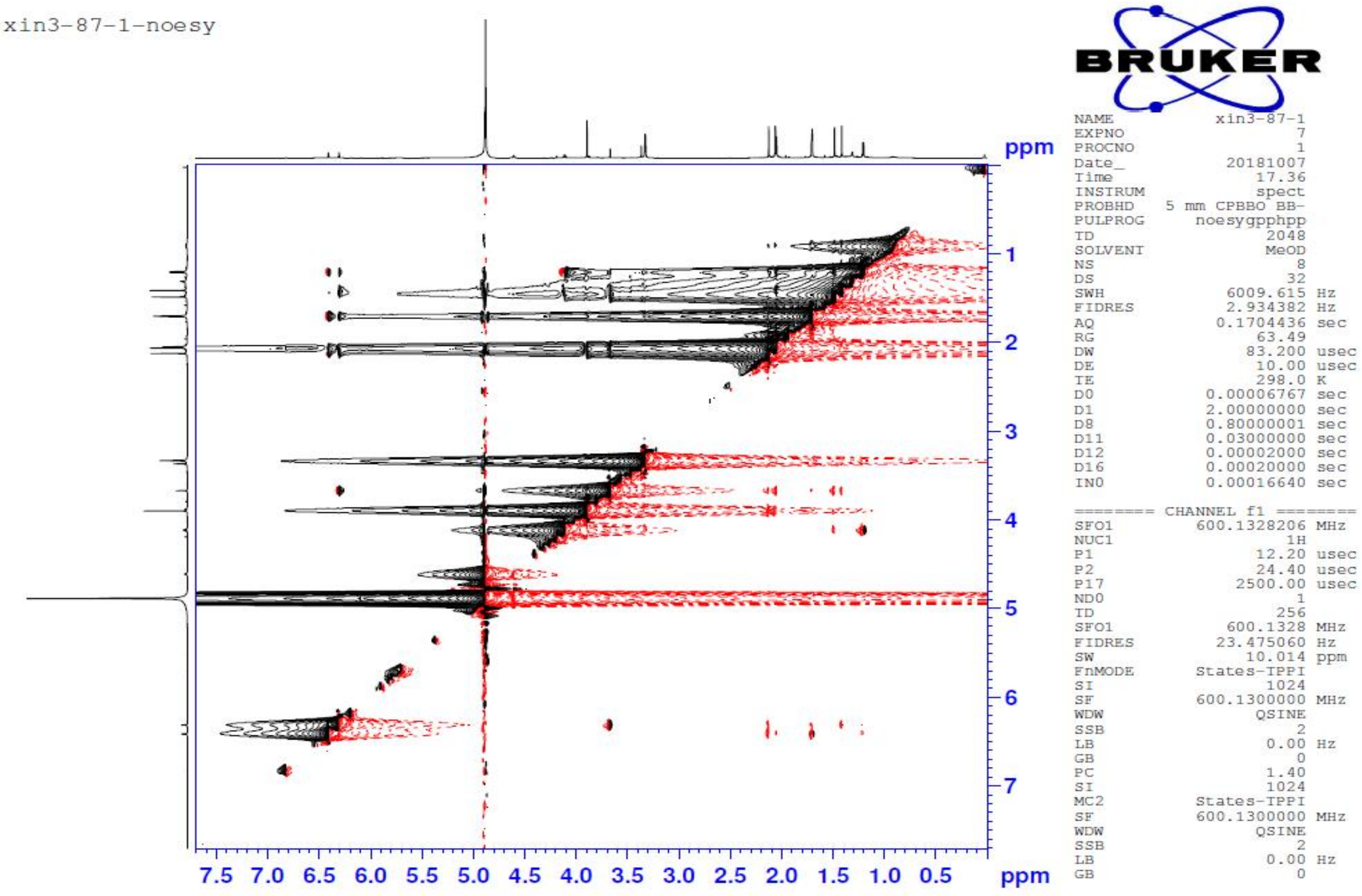

Figure S30. NOESY NMR spectrum of **5** in CD_3OD.

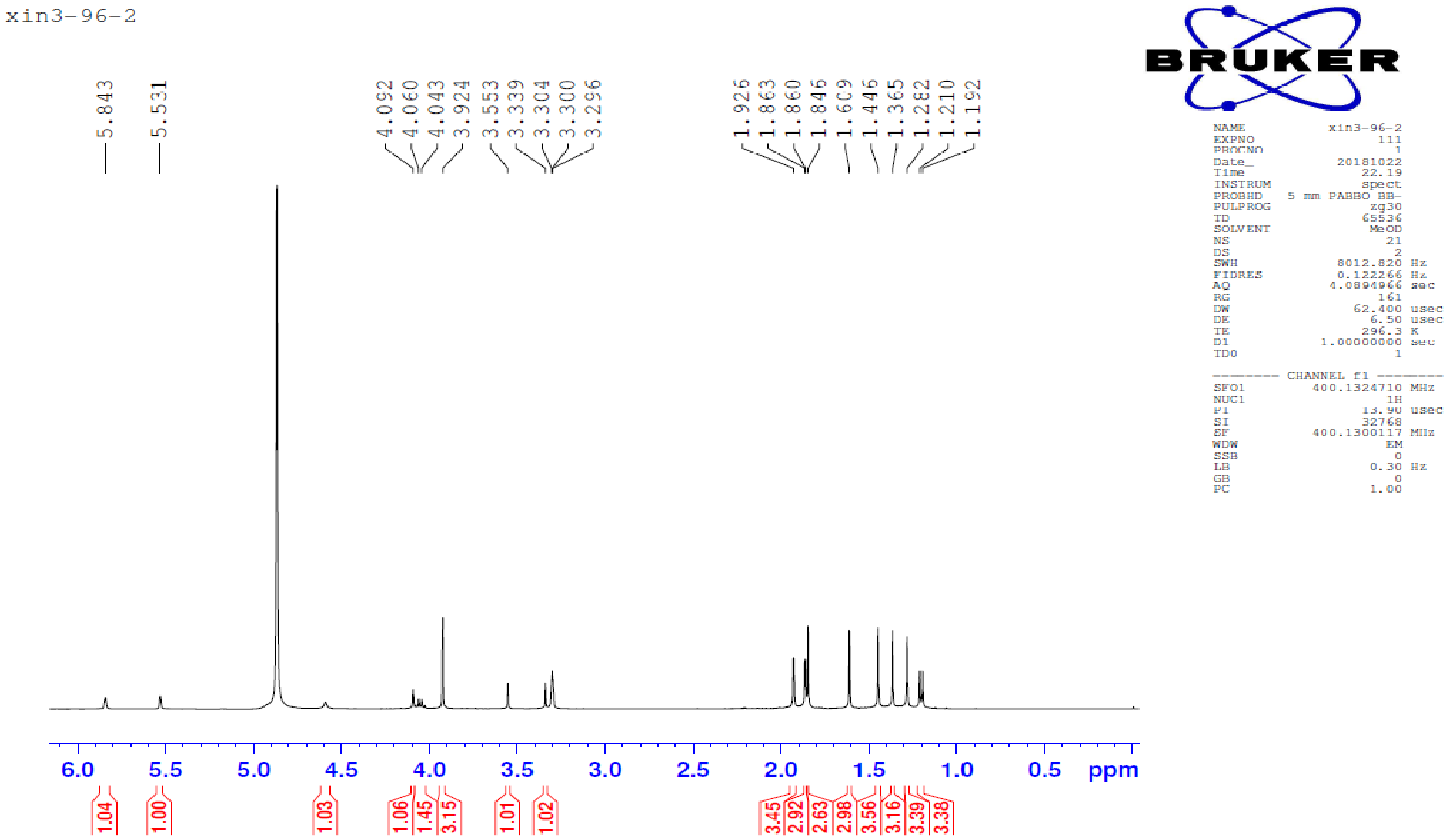

Figure S31. ^{1}H NMR spectrum of **6** in CD_3OD.

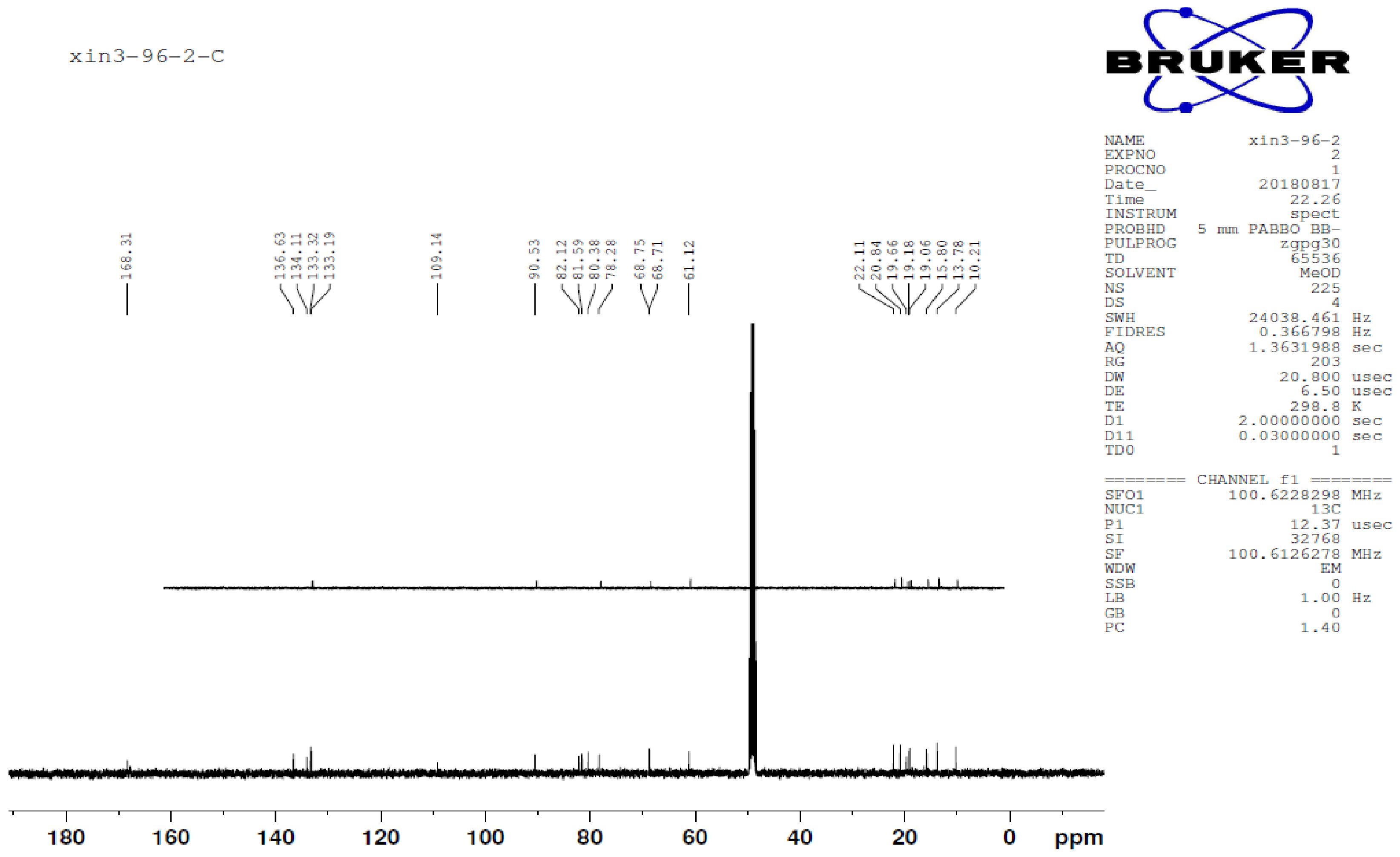

Figure S32. ^{13}C NMR spectrum of **6** in CD_3OD.

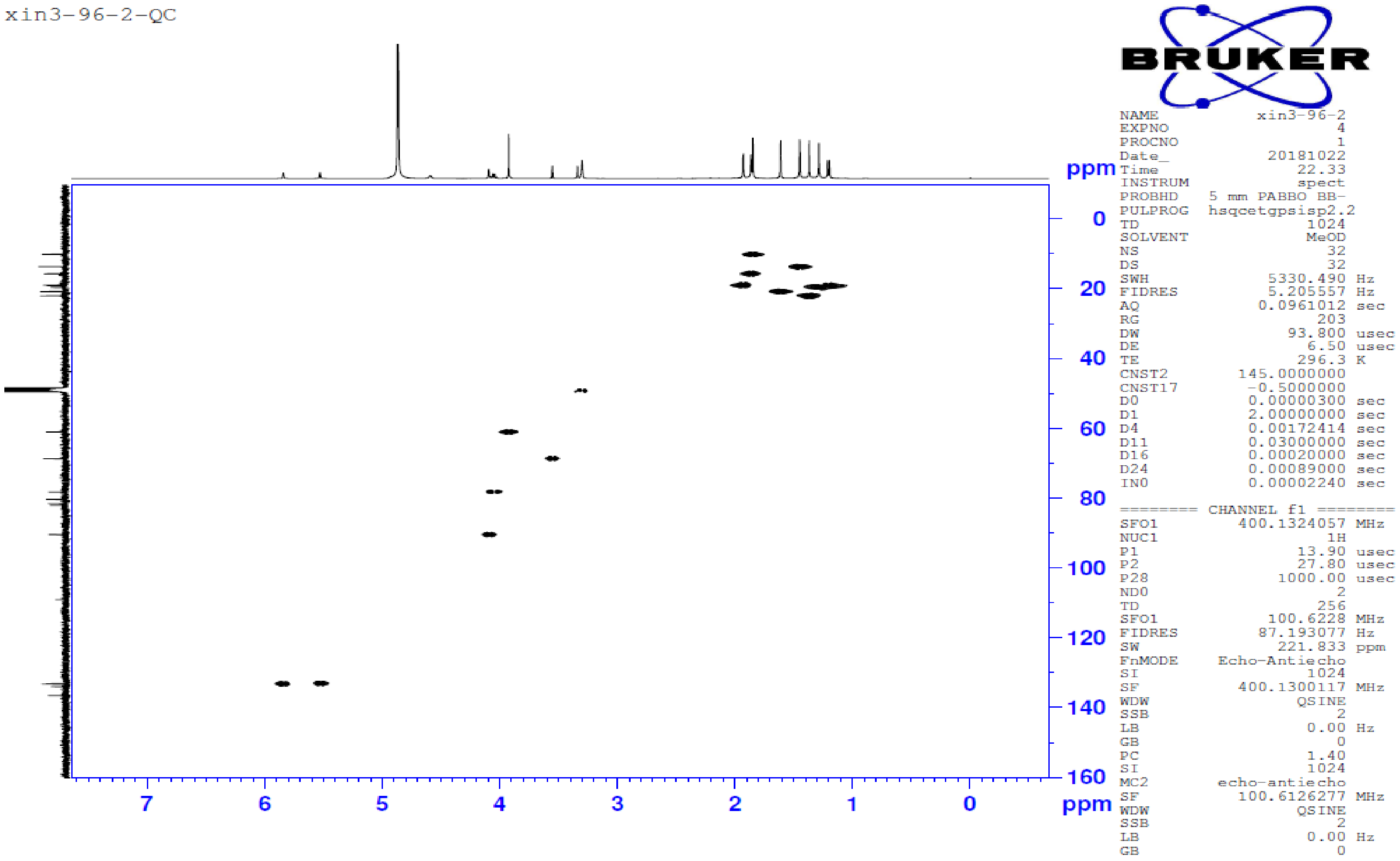

Figure S33. HSQC NMR spectrum of **6** in CD_3OD.

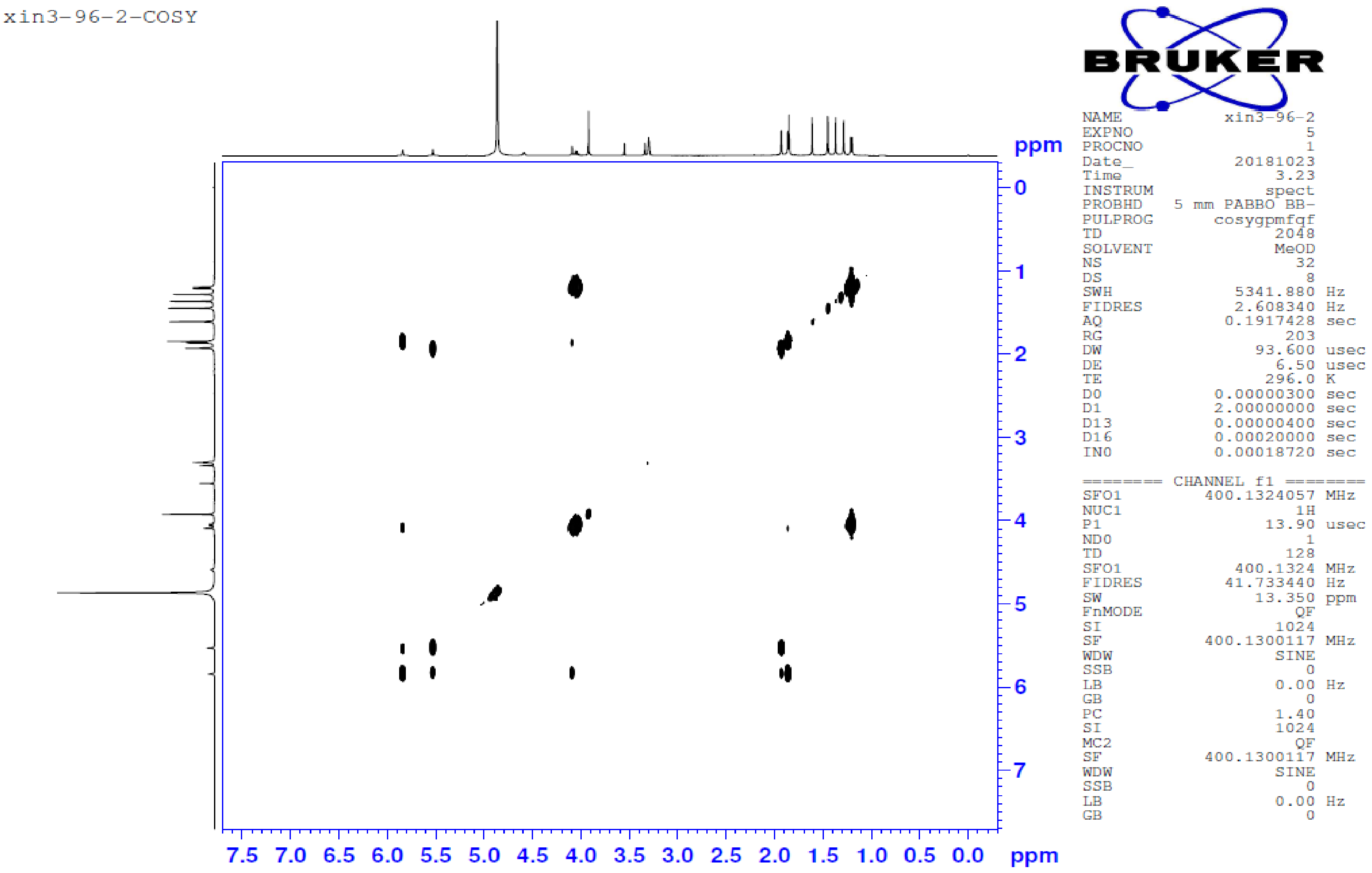

Figure S34. COSY NMR spectrum of **6** in CD_3OD.

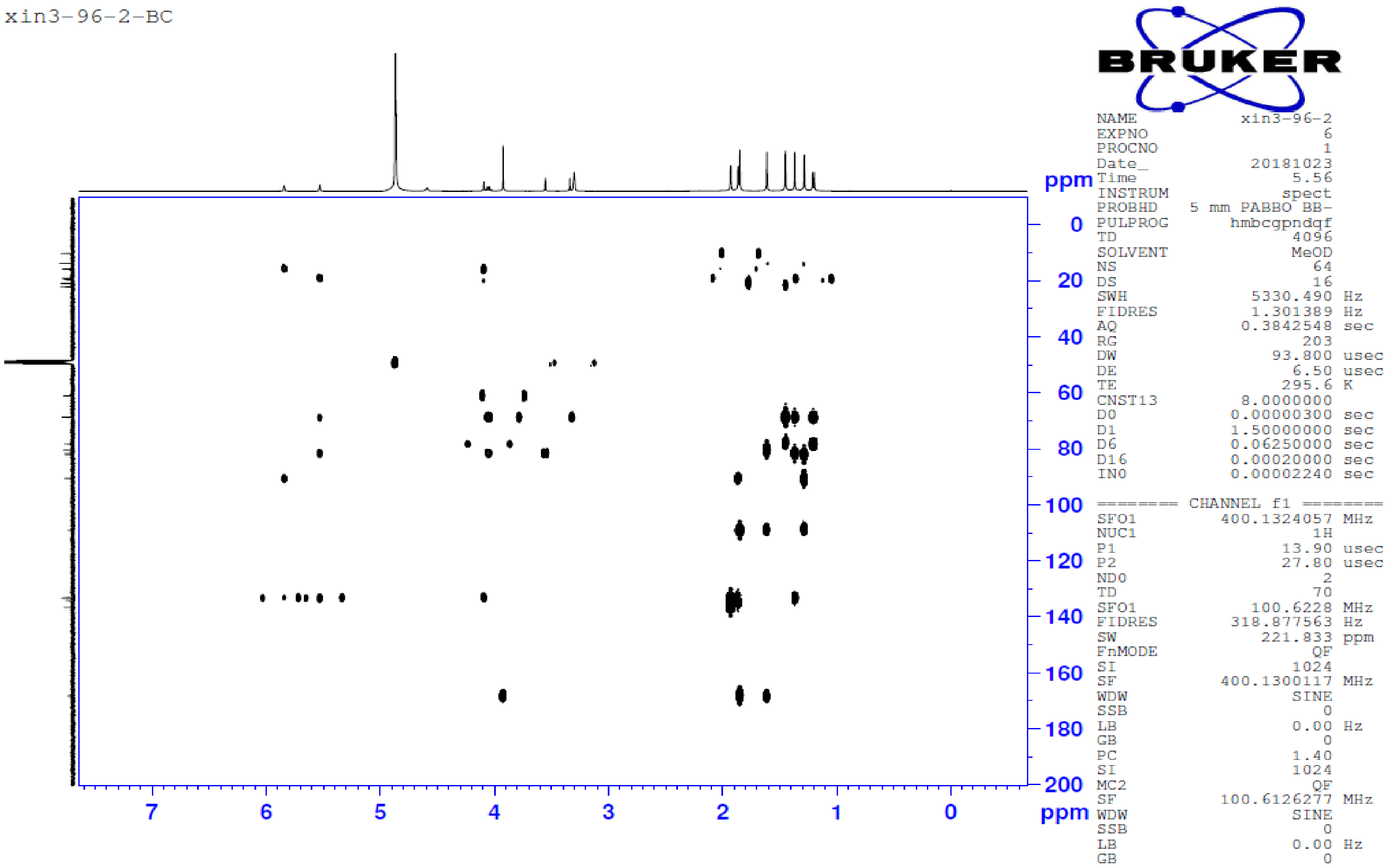

Figure S35. HMBC NMR spectrum of **6** in CD_3OD.

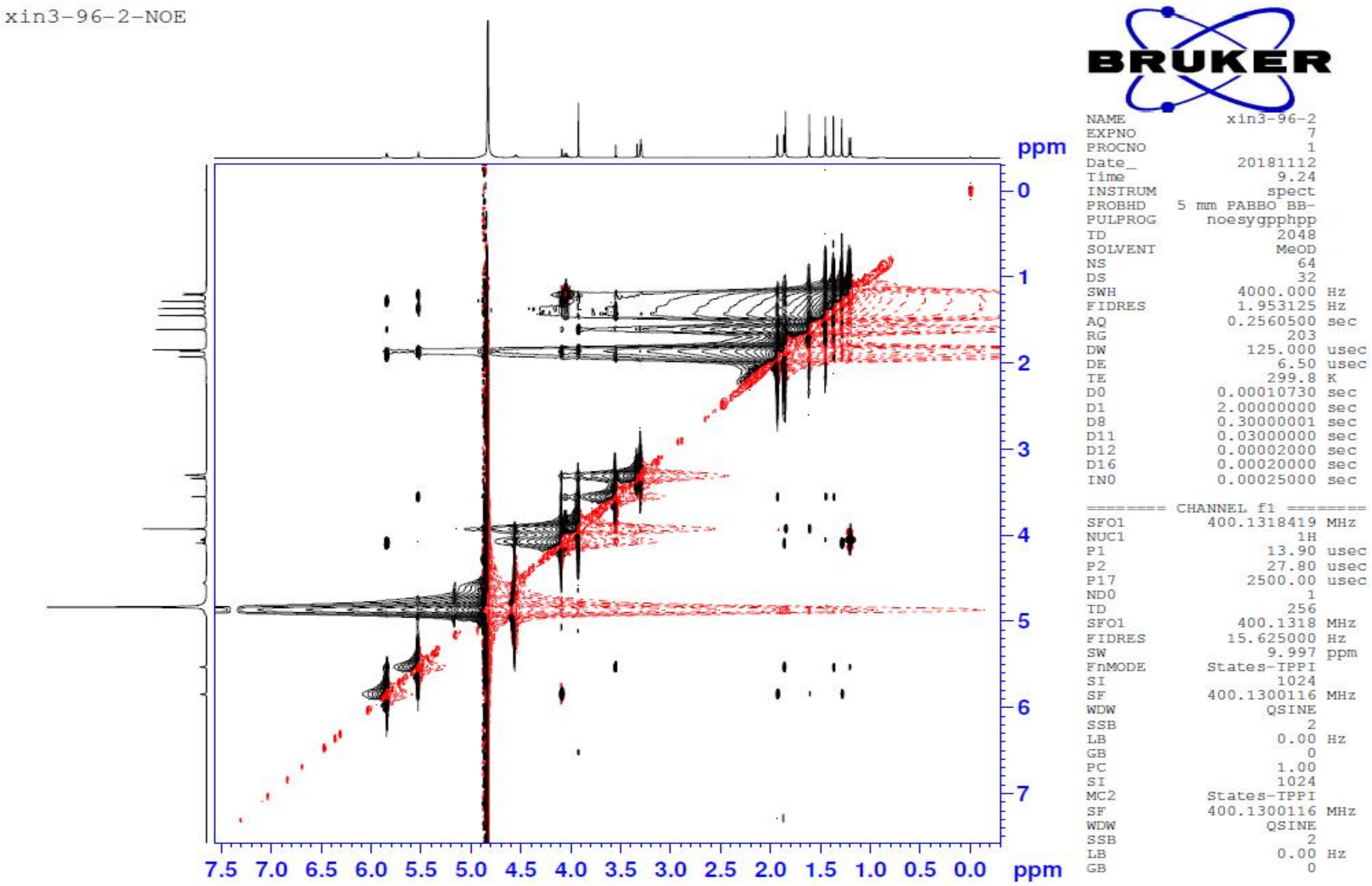

Figure S36. NOESY NMR spectrum of **6** in CD_3OD.

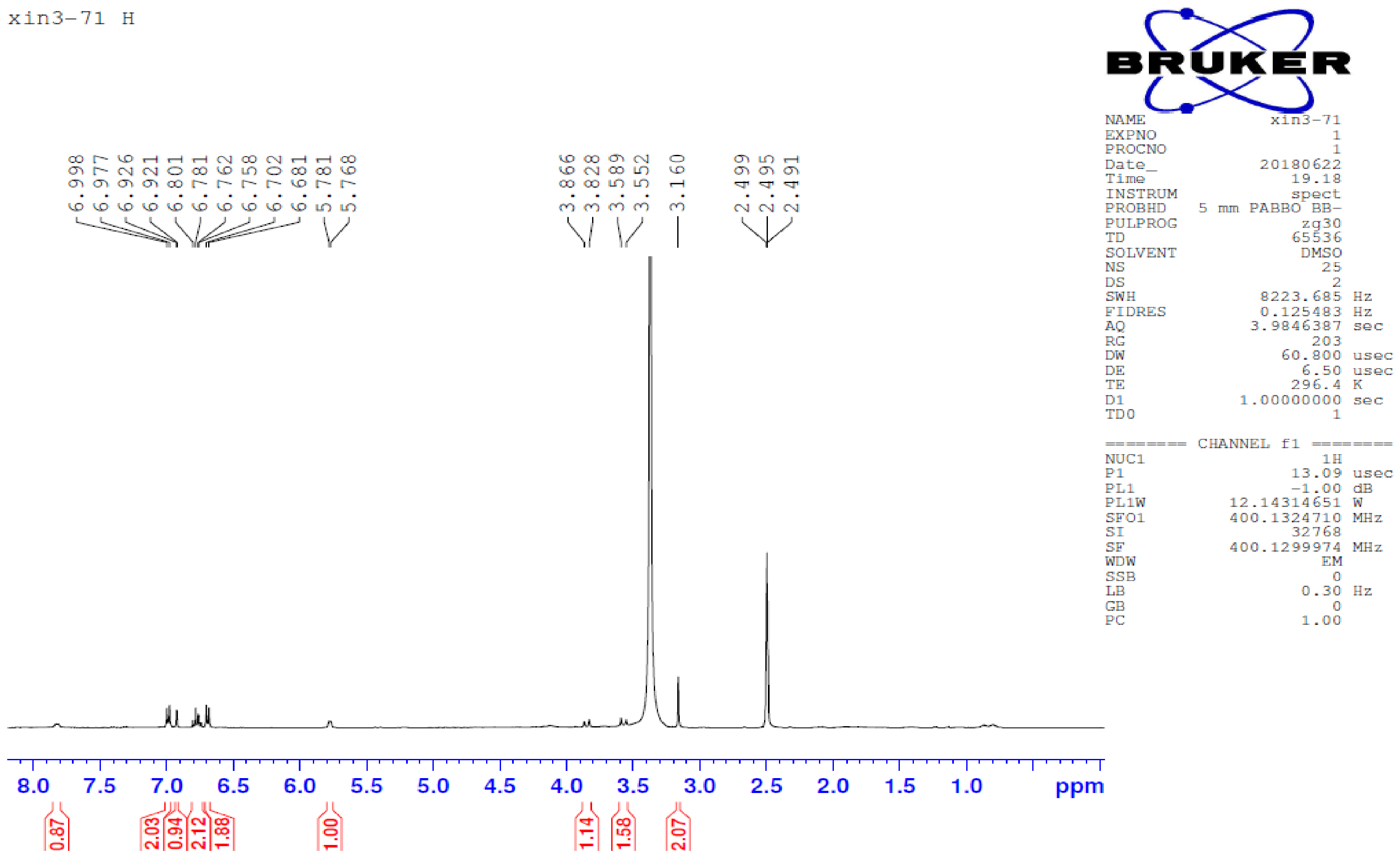

Figure S37. ^{1}H NMR spectrum of **7** in DMSO-d_6.

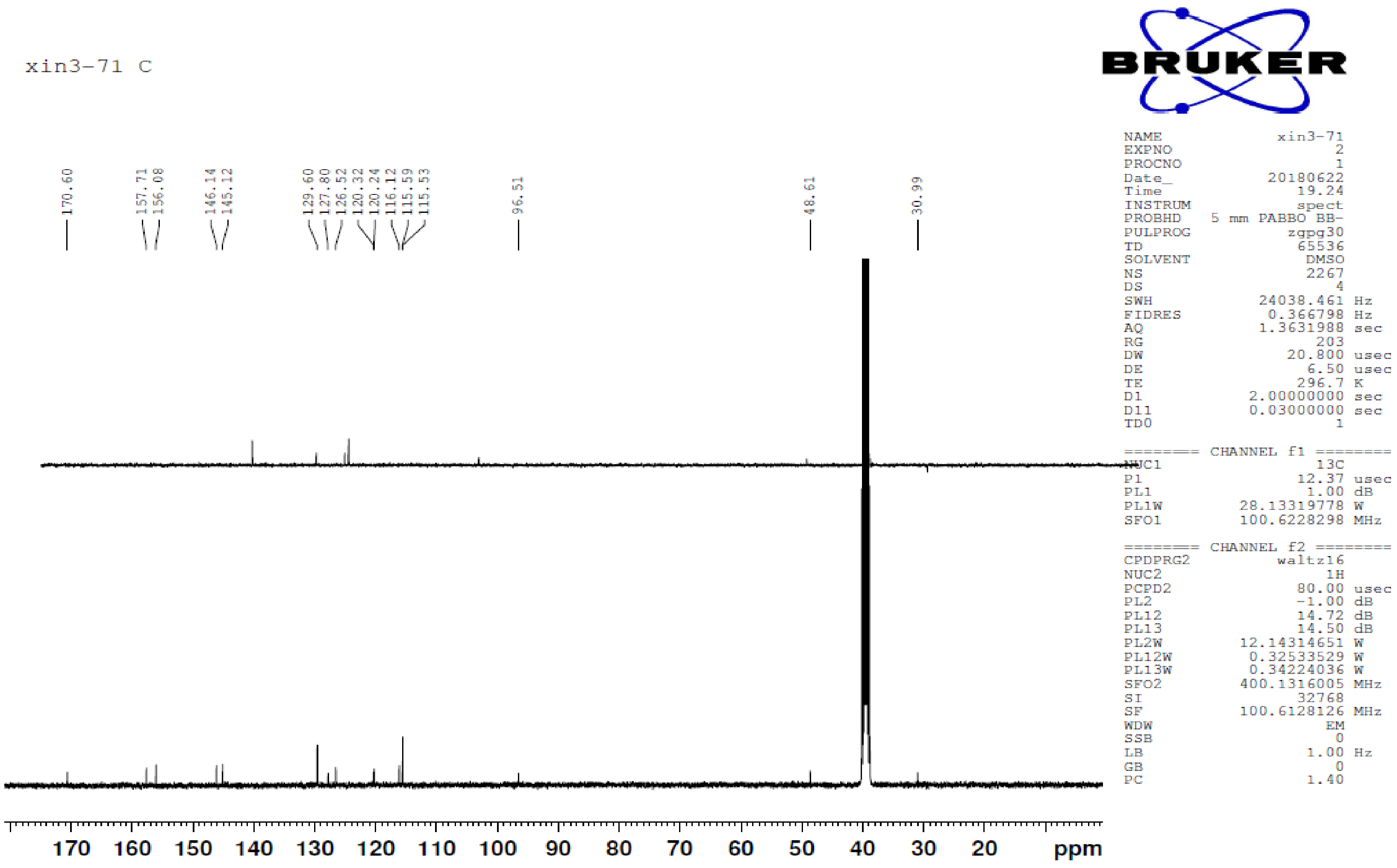

Figure S38. ^{13}C NMR spectrum of **7** in DMSO-d_6.

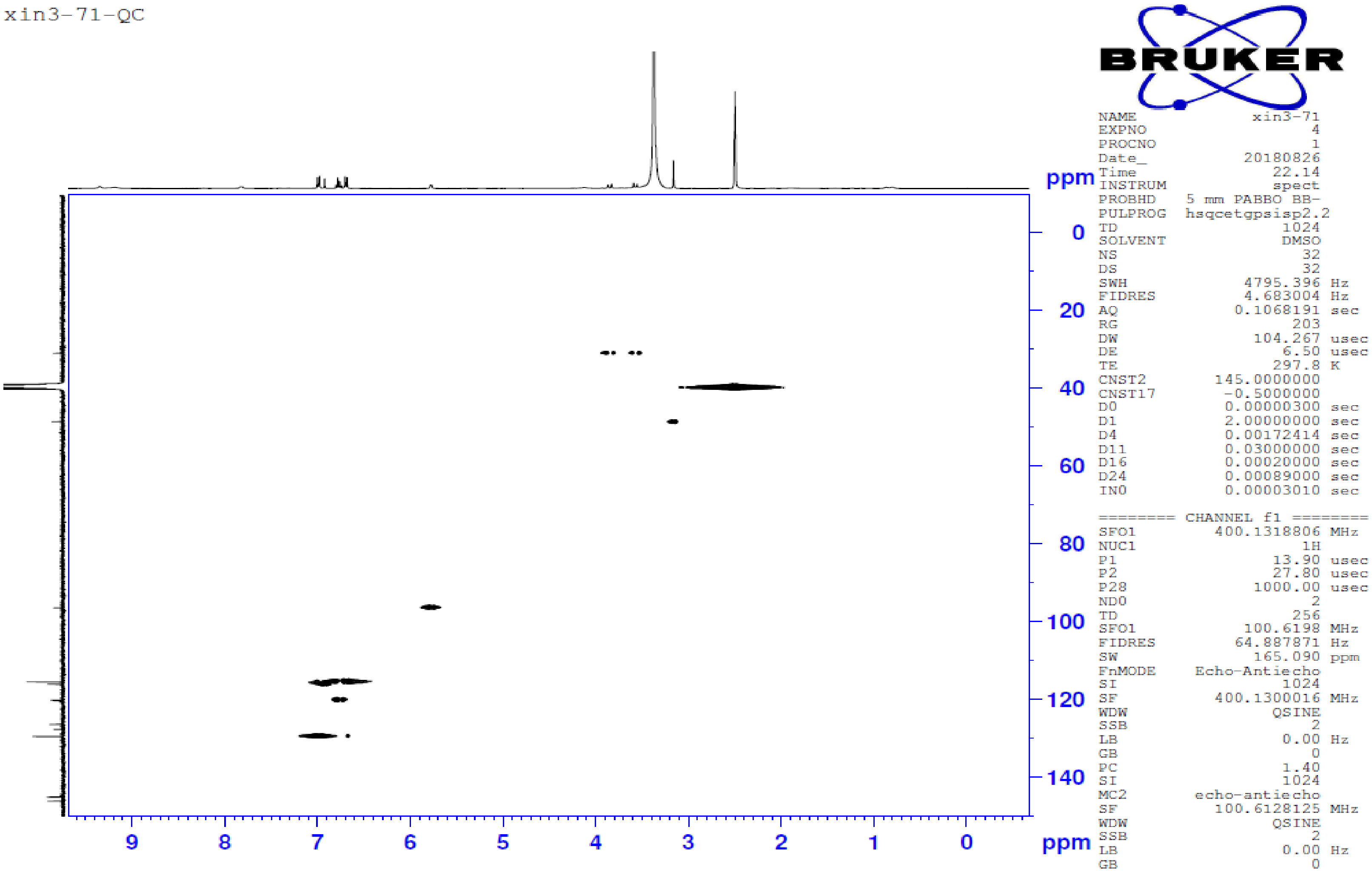

Figure S39. HSQC NMR spectrum of **7** in DMSO-d_6.

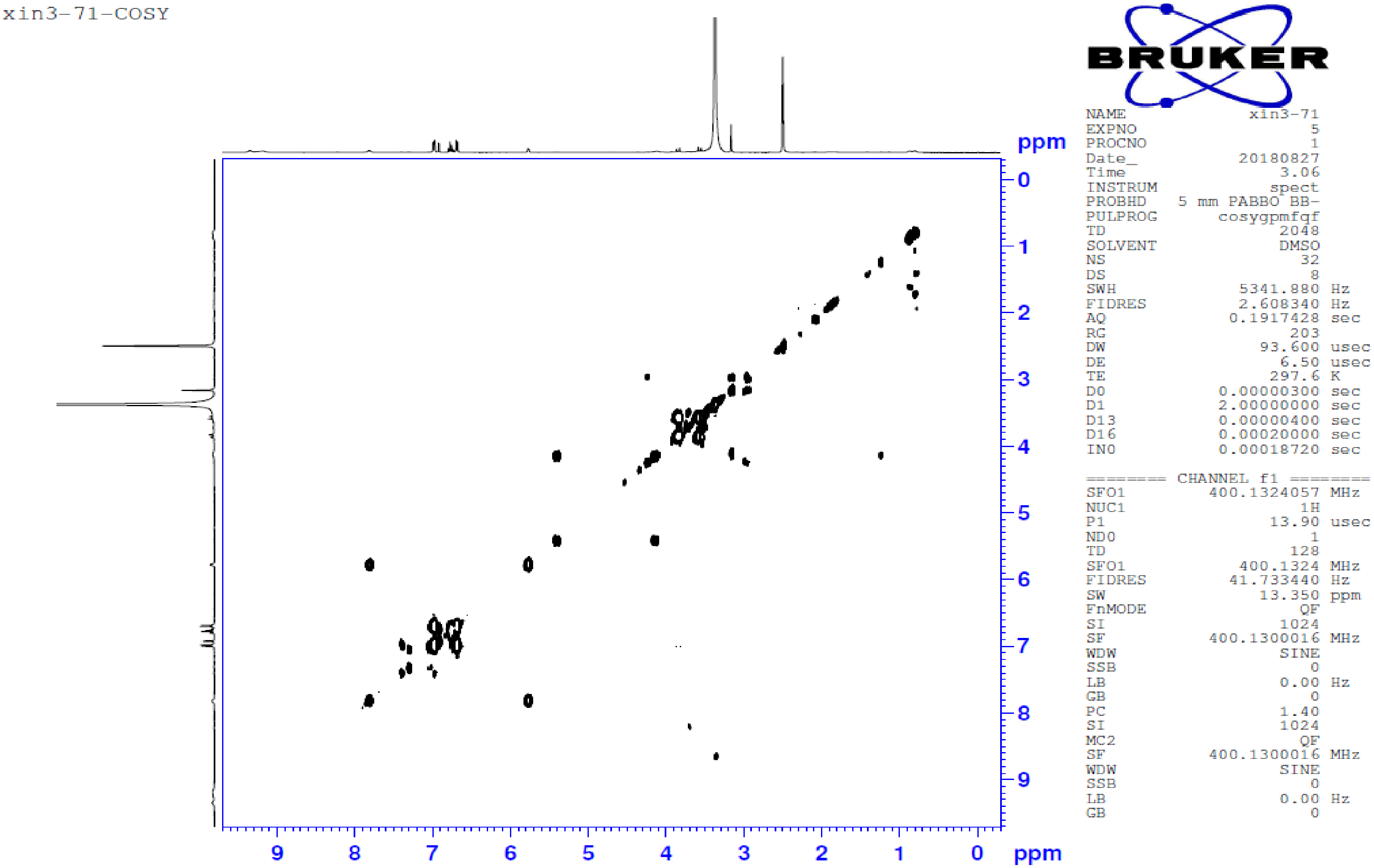

Figure S40. COSY NMR spectrum of **7** in DMSO-d_6.

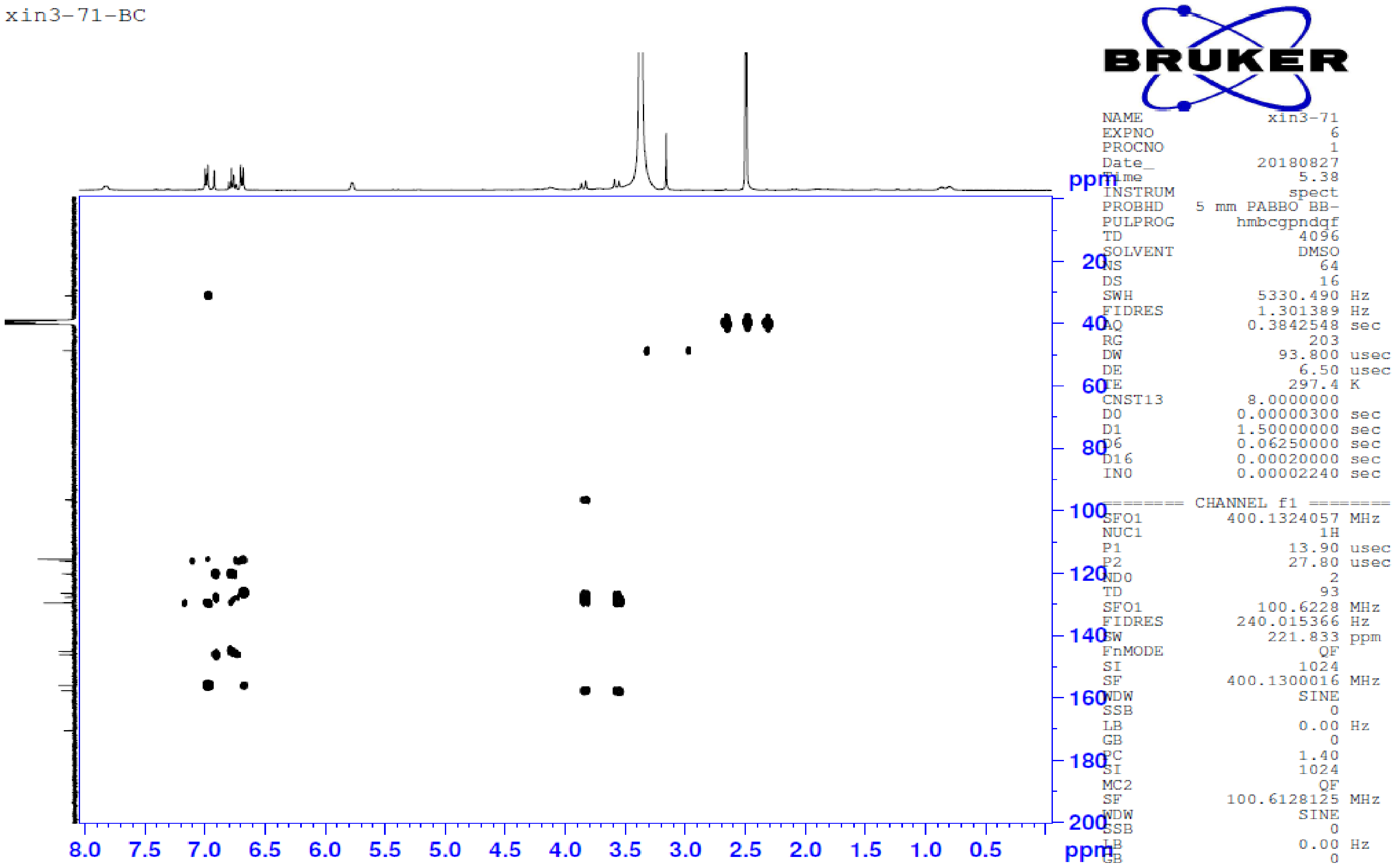

Figure S41. HMBC NMR spectrum of **7** in DMSO-d_6.

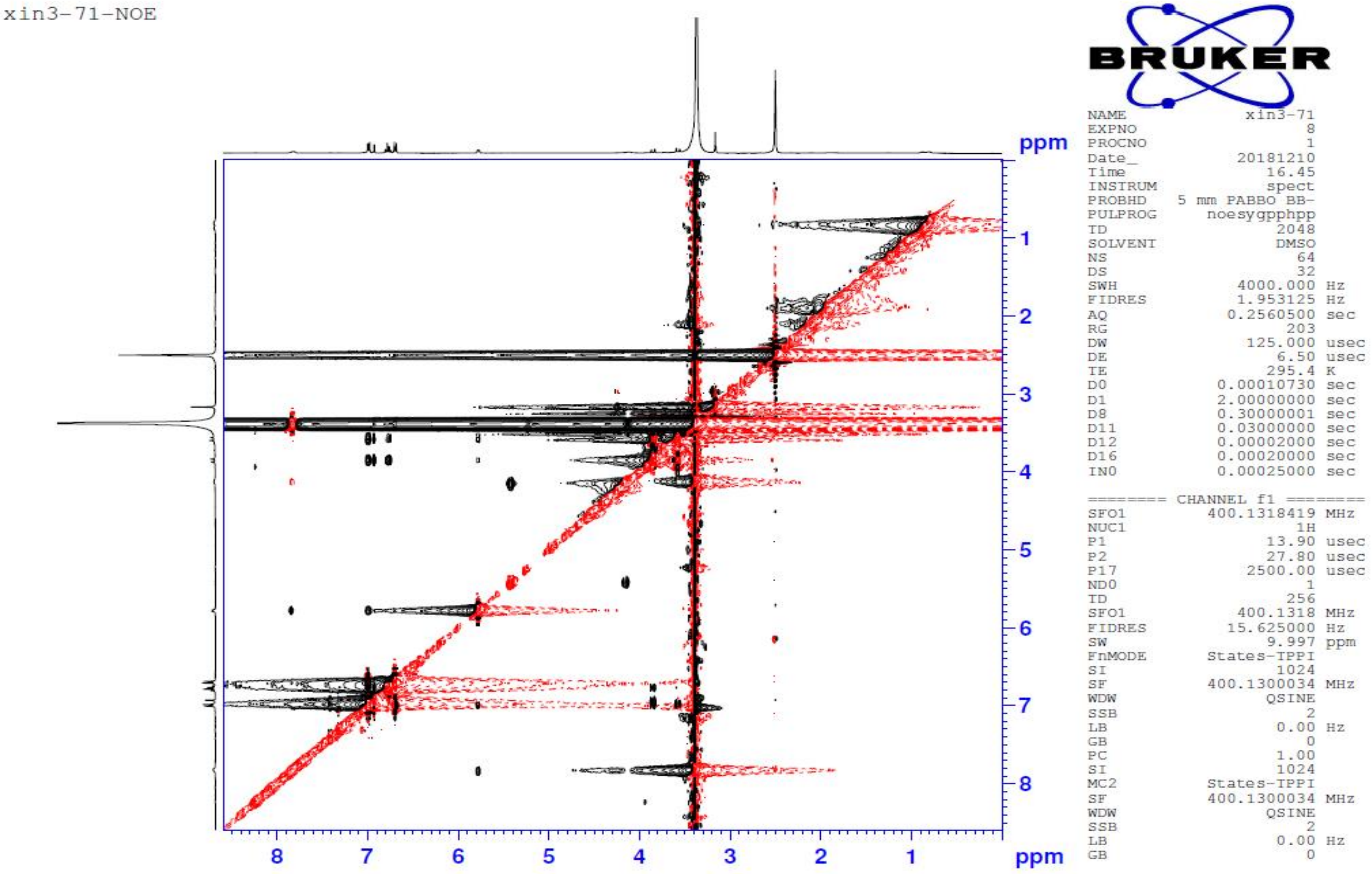

Figure S42. NOESY NMR spectrum of **7** in DMSO-d_6.

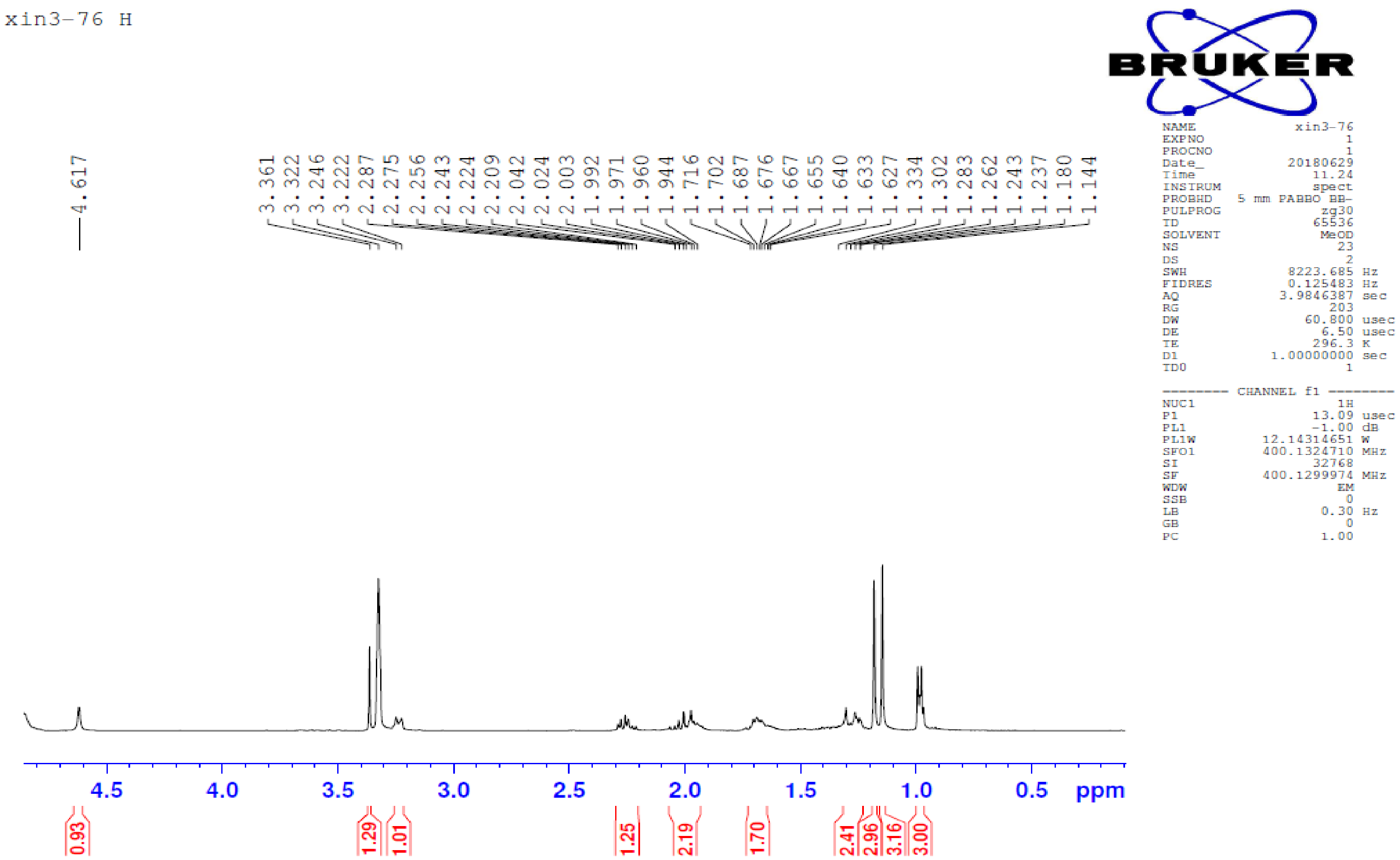

Figure S43. ^{1}H NMR spectrum of **8** in CD_3OD.

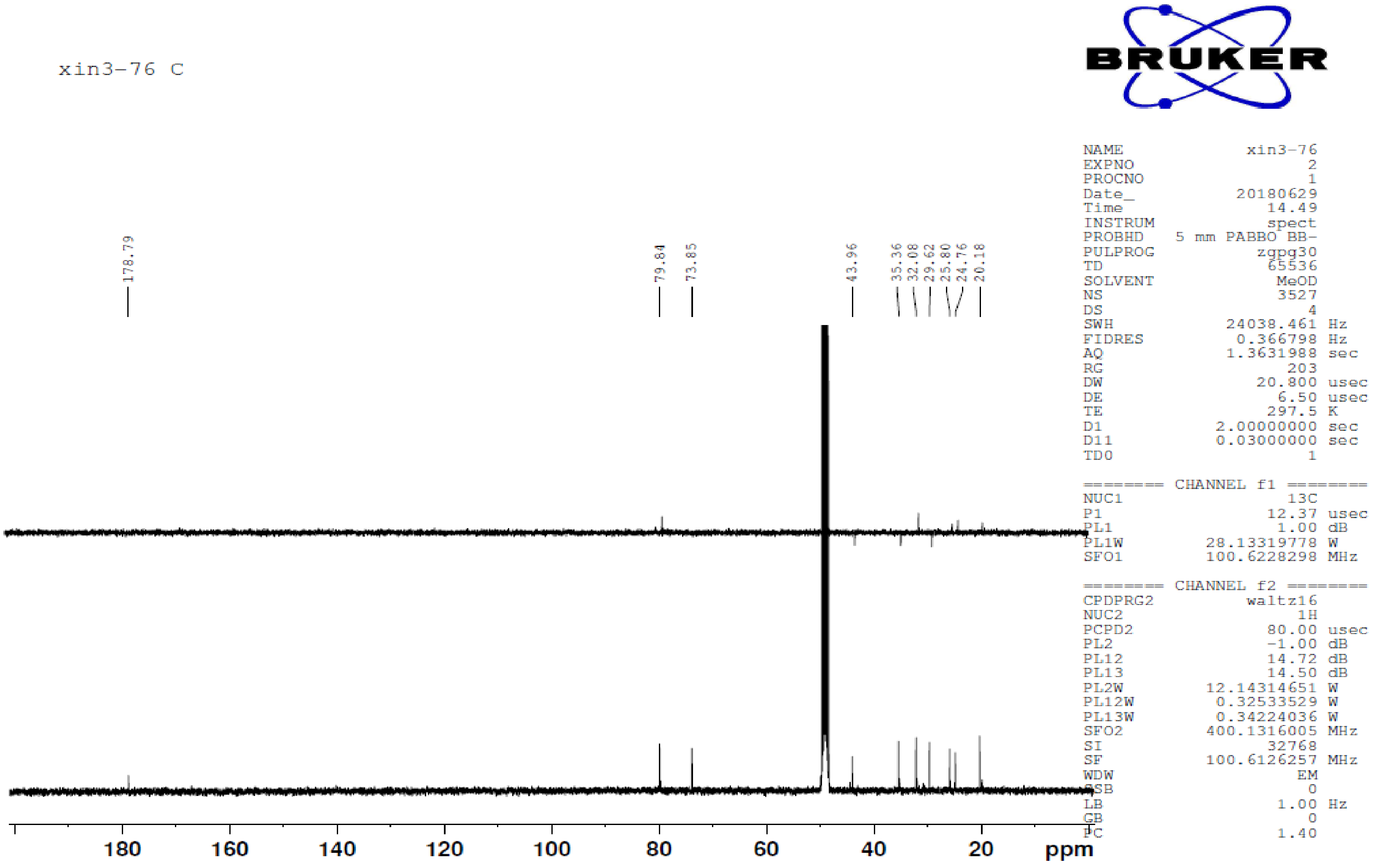

Figure S44. ^{13}C NMR spectrum of **8** in CD_3OD.

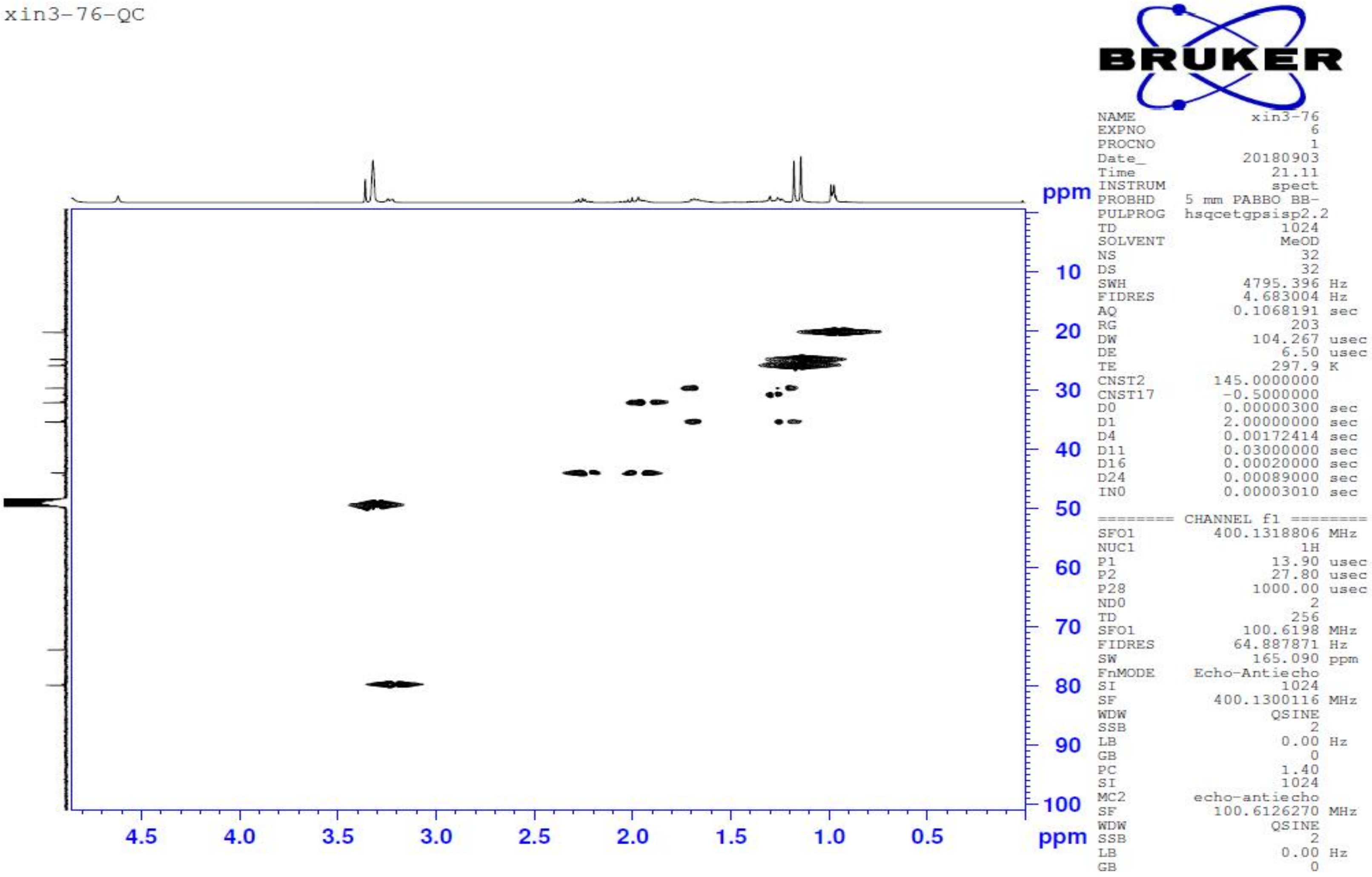

Figure S45. HSQC NMR spectrum of **8** in CD_3OD.

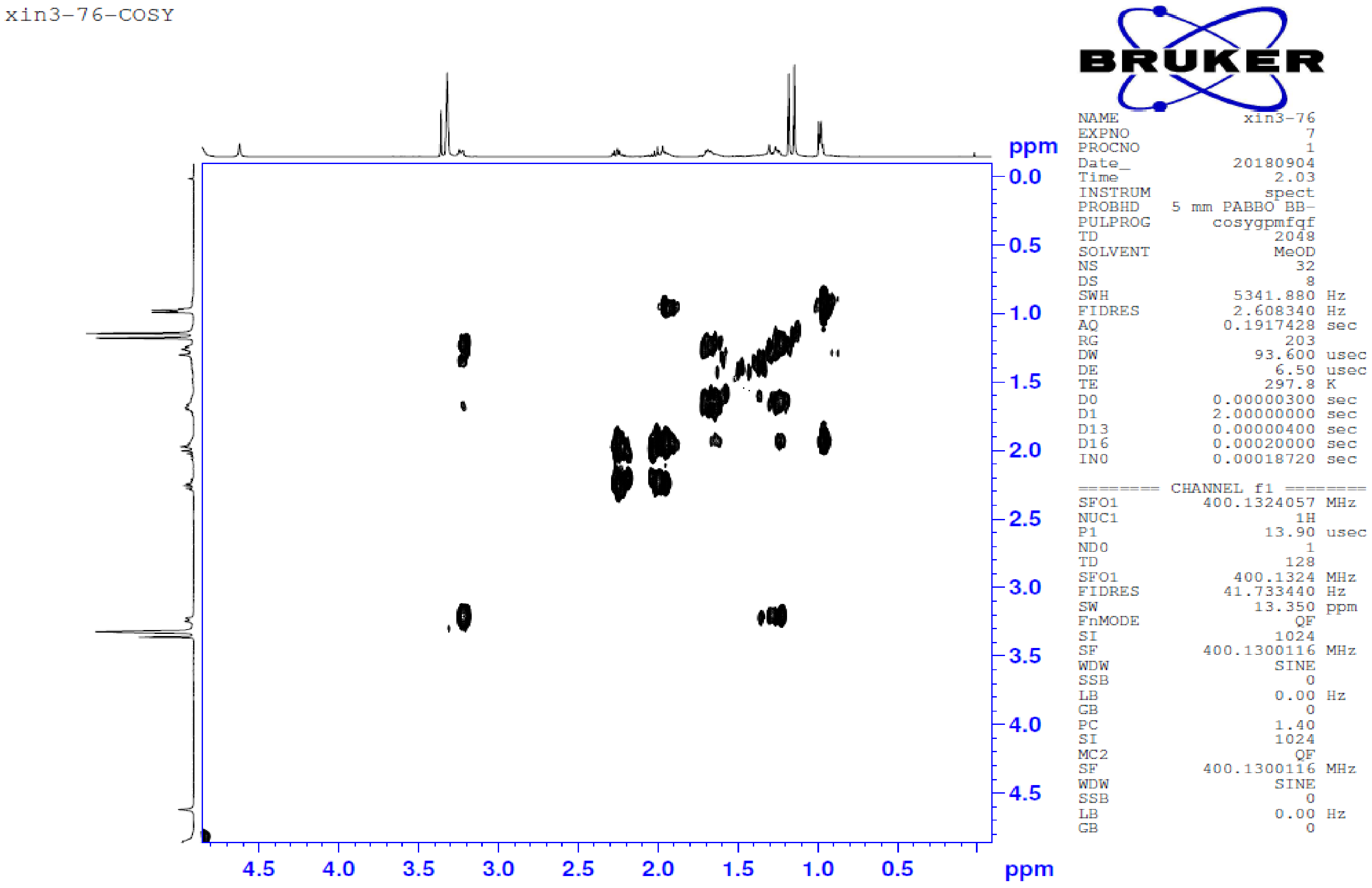

Figure S46. COSY NMR spectrum of **8** in CD_3OD.

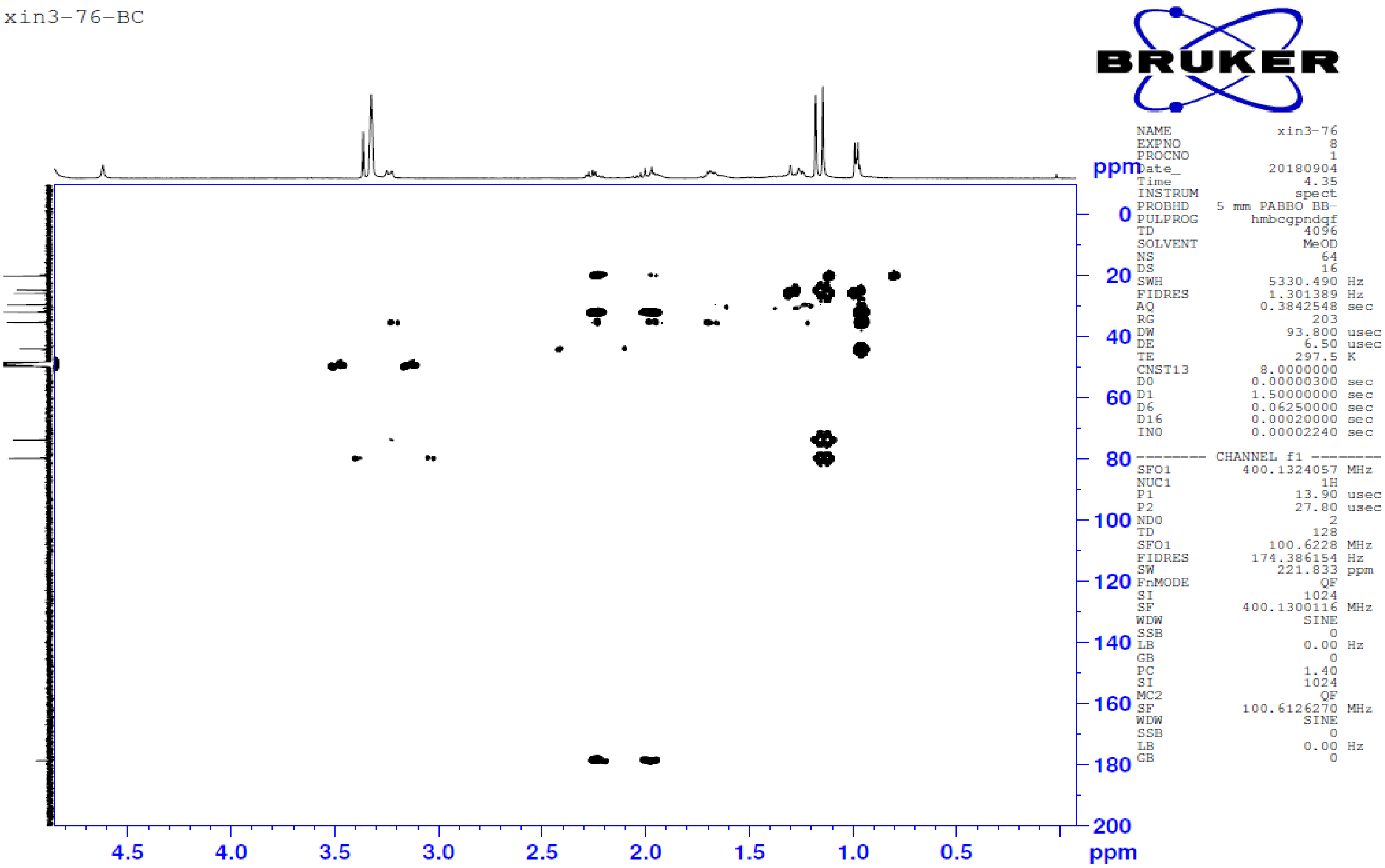

Figure S47. HMBC NMR spectrum of **8** in CD_3OD.

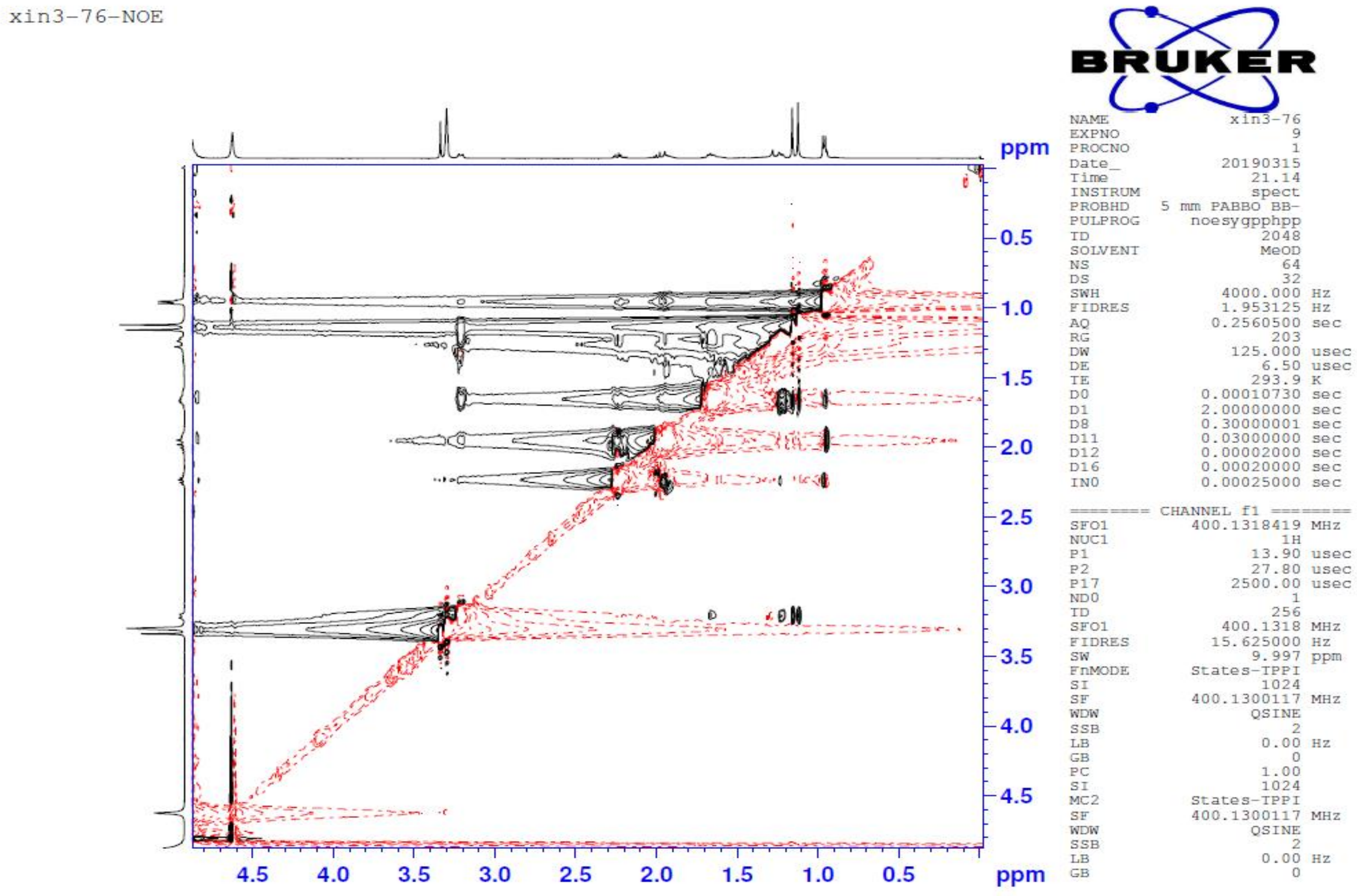

Figure S48. NOESY NMR spectrum of **8** in CD_3OD.

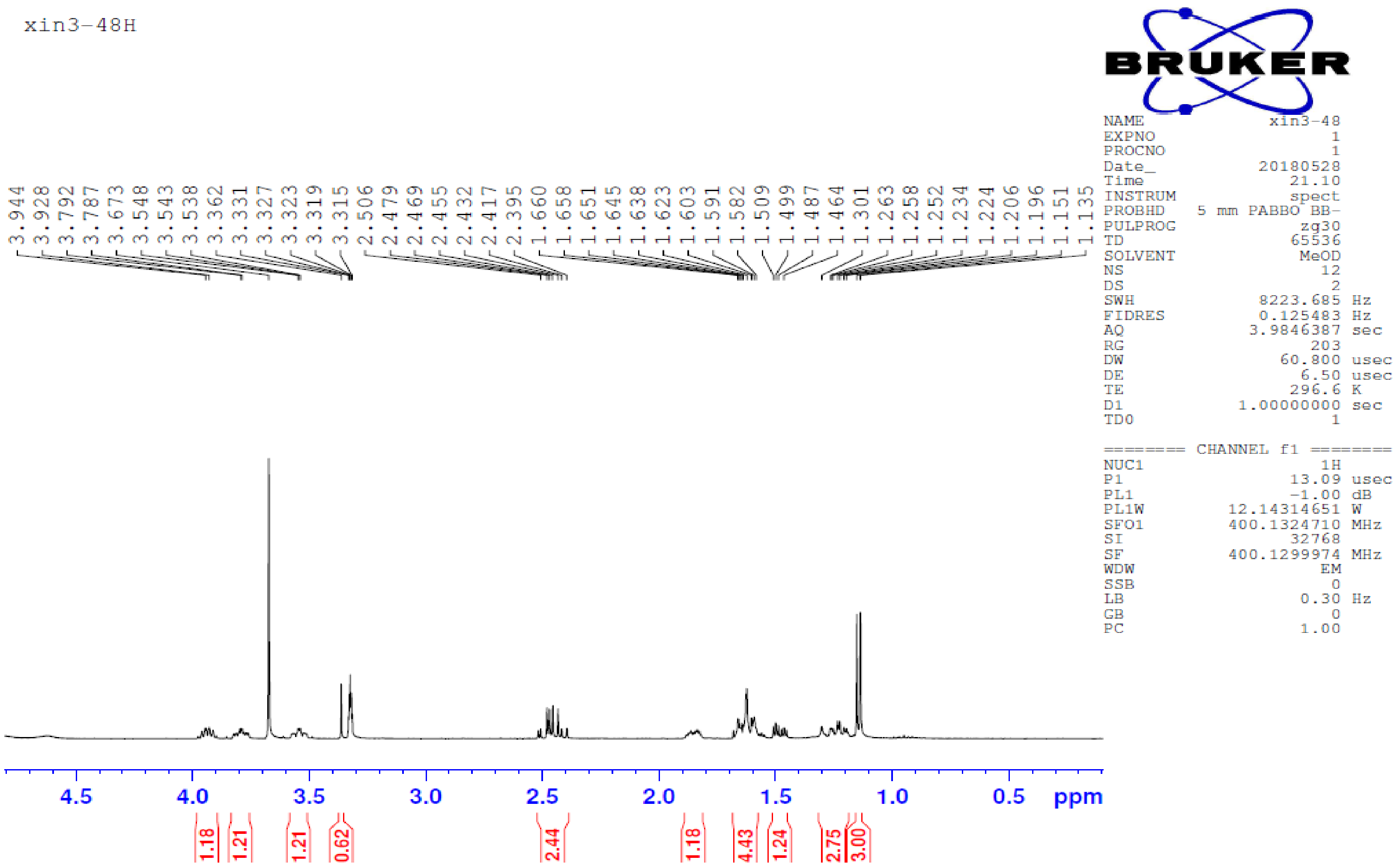

Figure S49. ^{1}H NMR spectrum of **9** in CD_3OD.

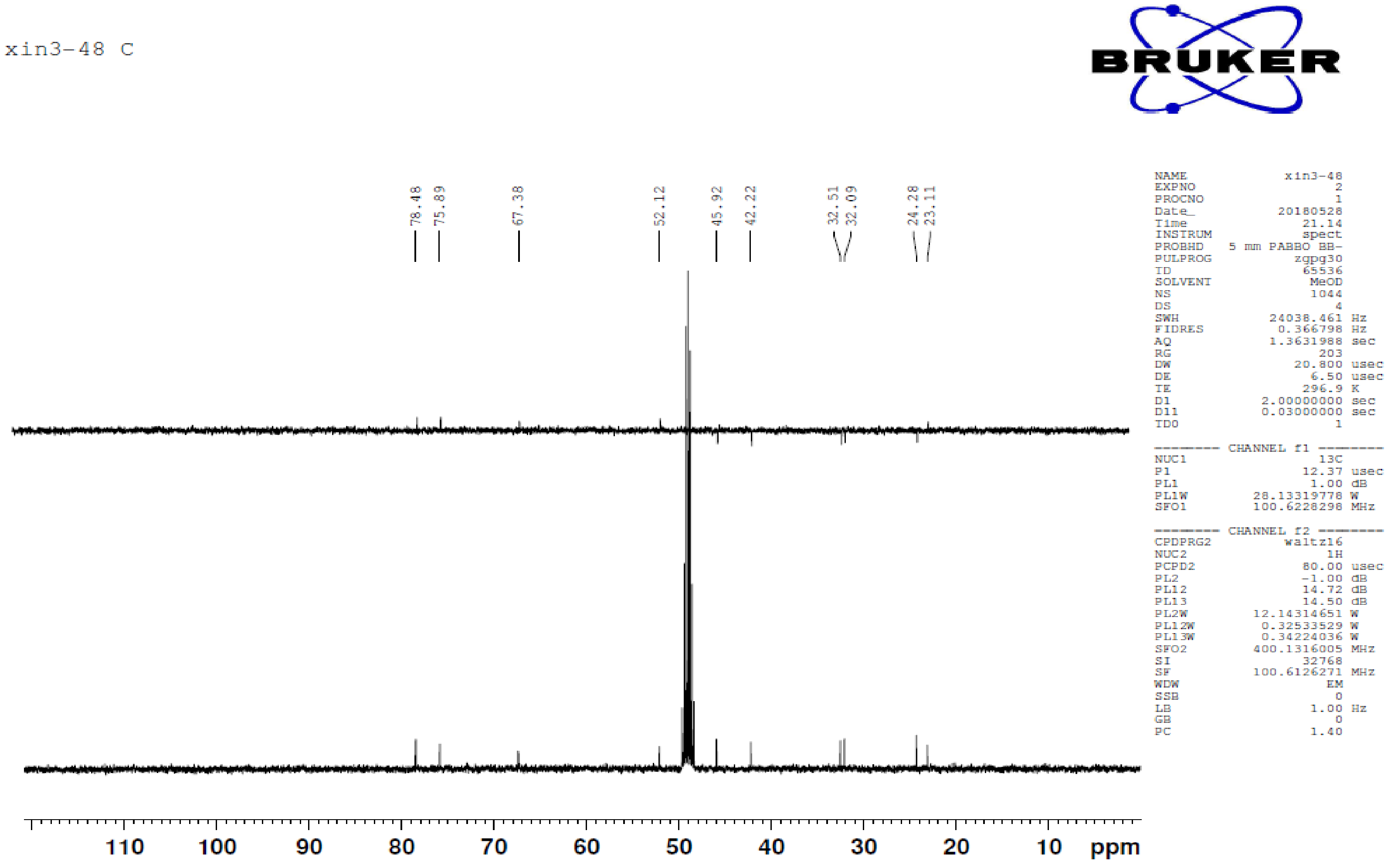

Figure S50. ^{13}C NMR spectrum of **9** in CD_3OD.

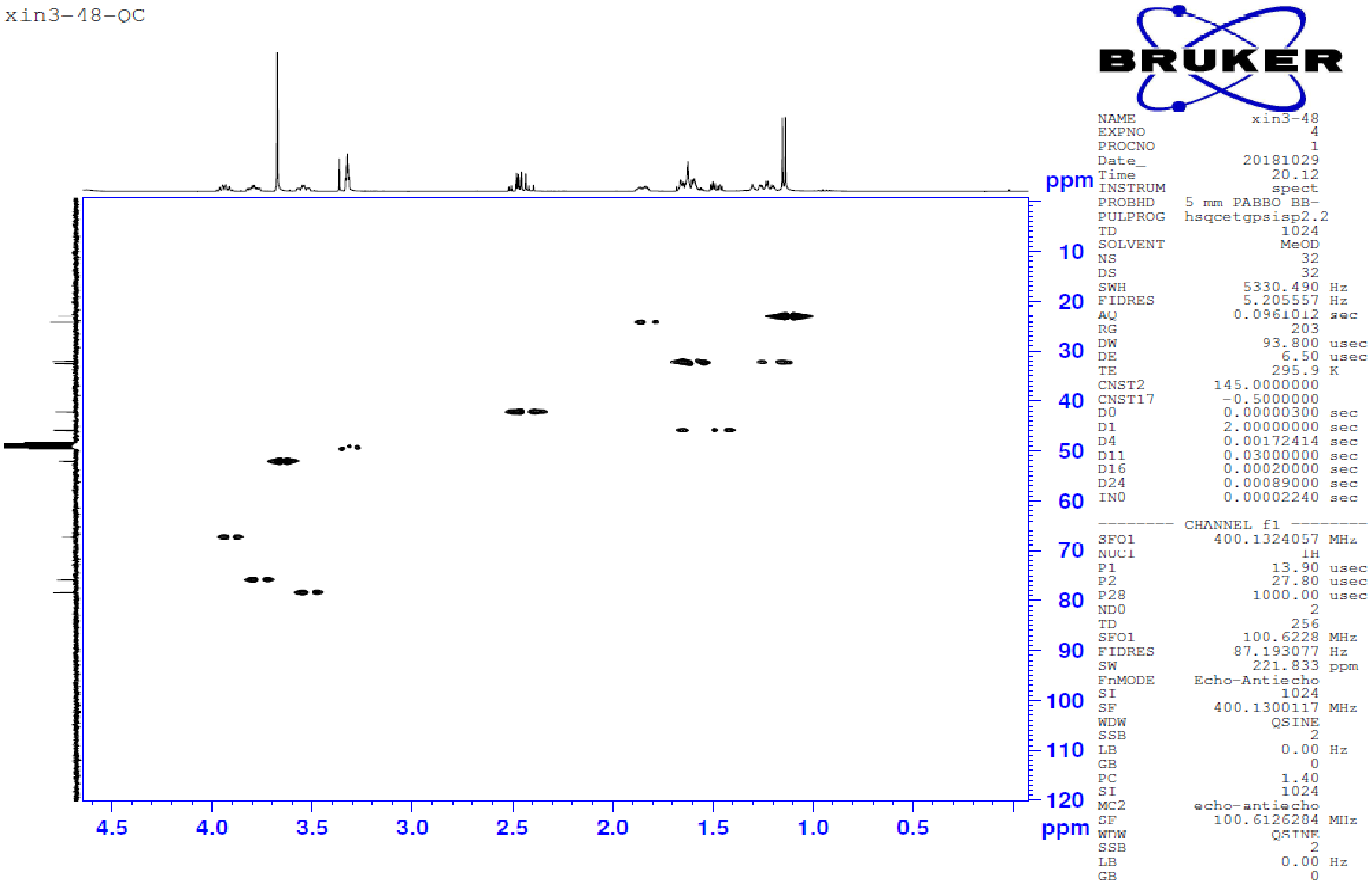

Figure S51. HSQC NMR spectrum of **9** in CD_3OD.

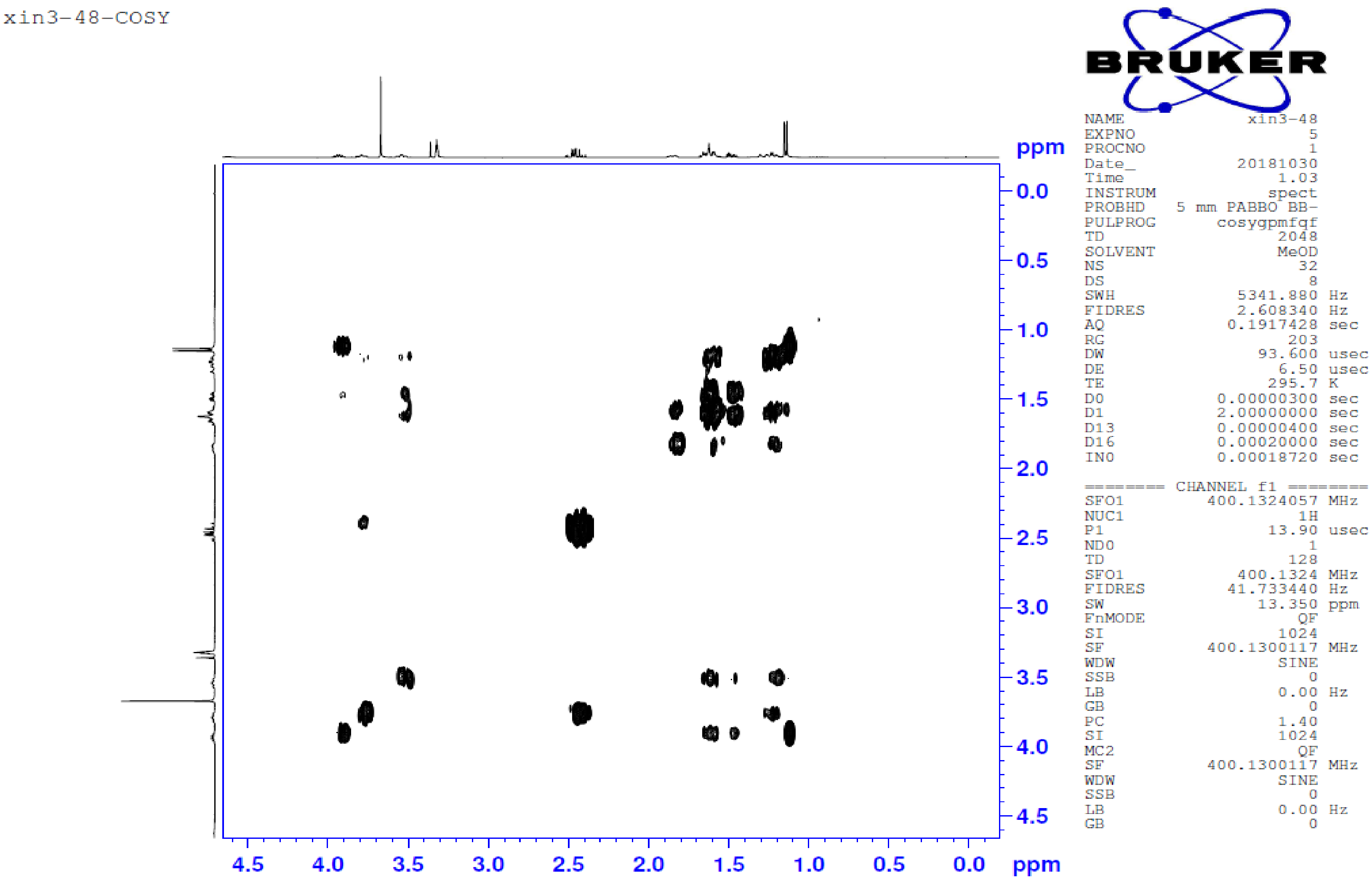

Figure S52. COSY NMR spectrum of **9** in CD_3OD.

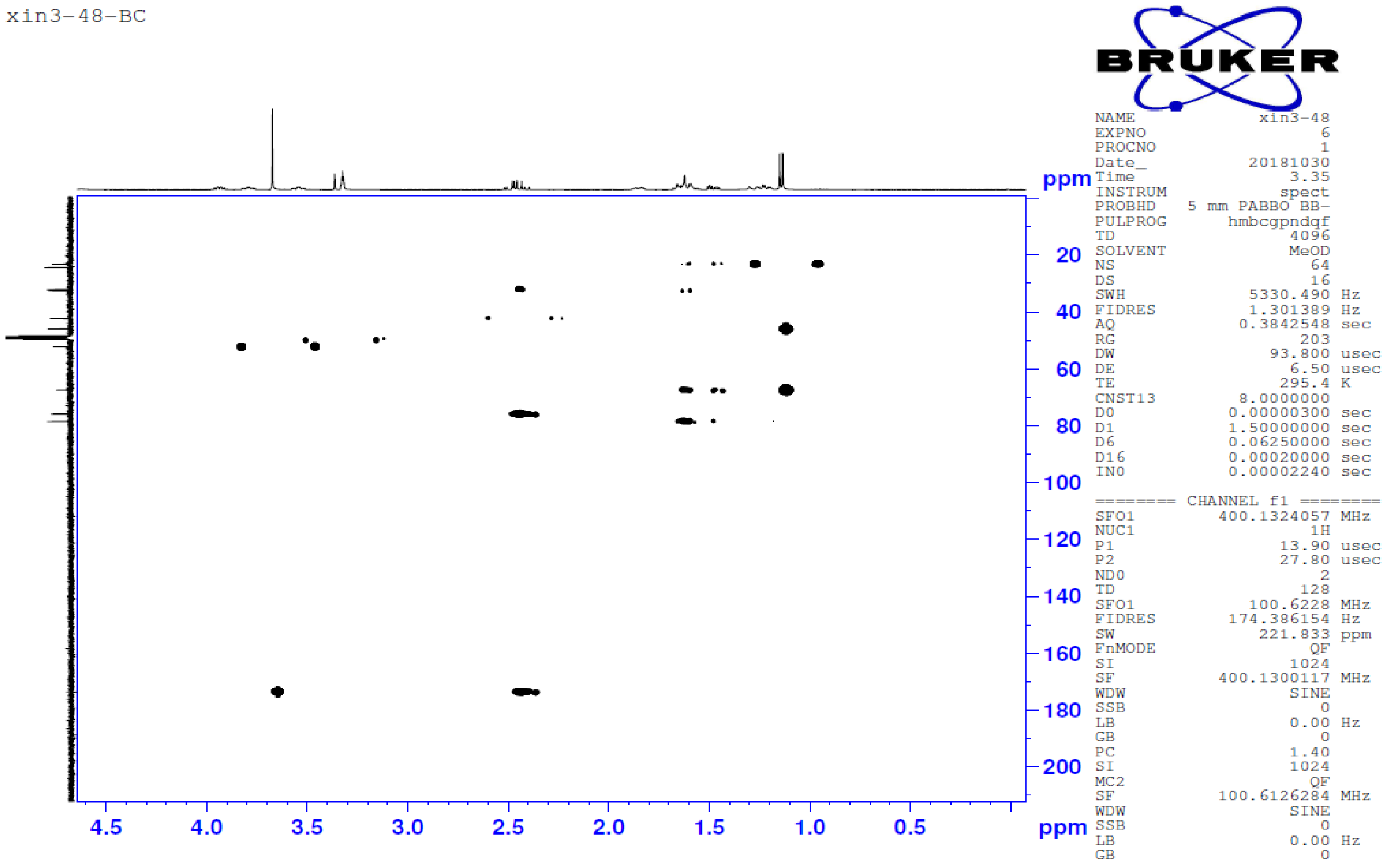

Figure S53. HMBC NMR spectrum of **9** in CD_3OD.

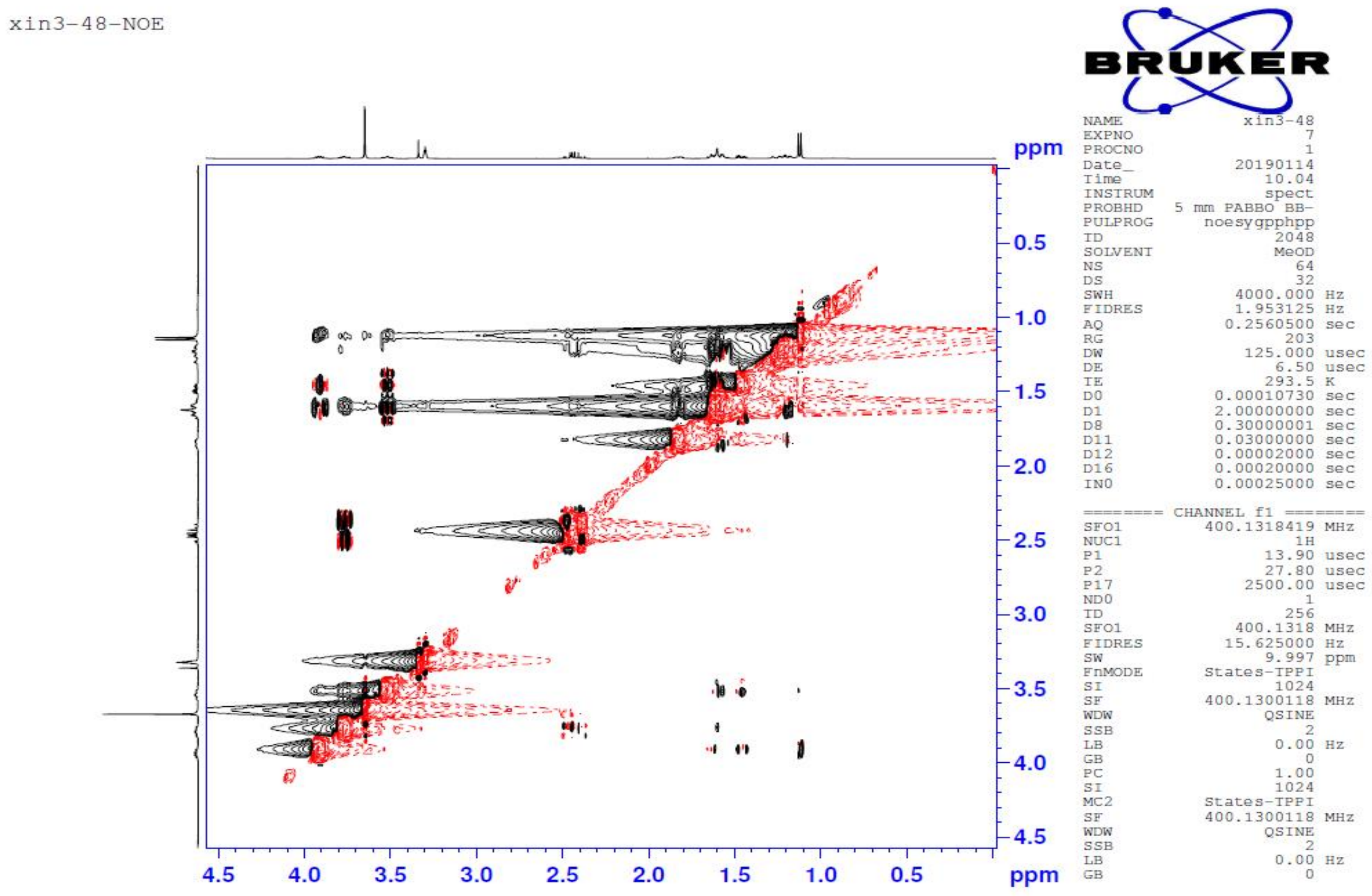

Figure S54. NOESY NMR spectrum of **9** in CD_3OD.

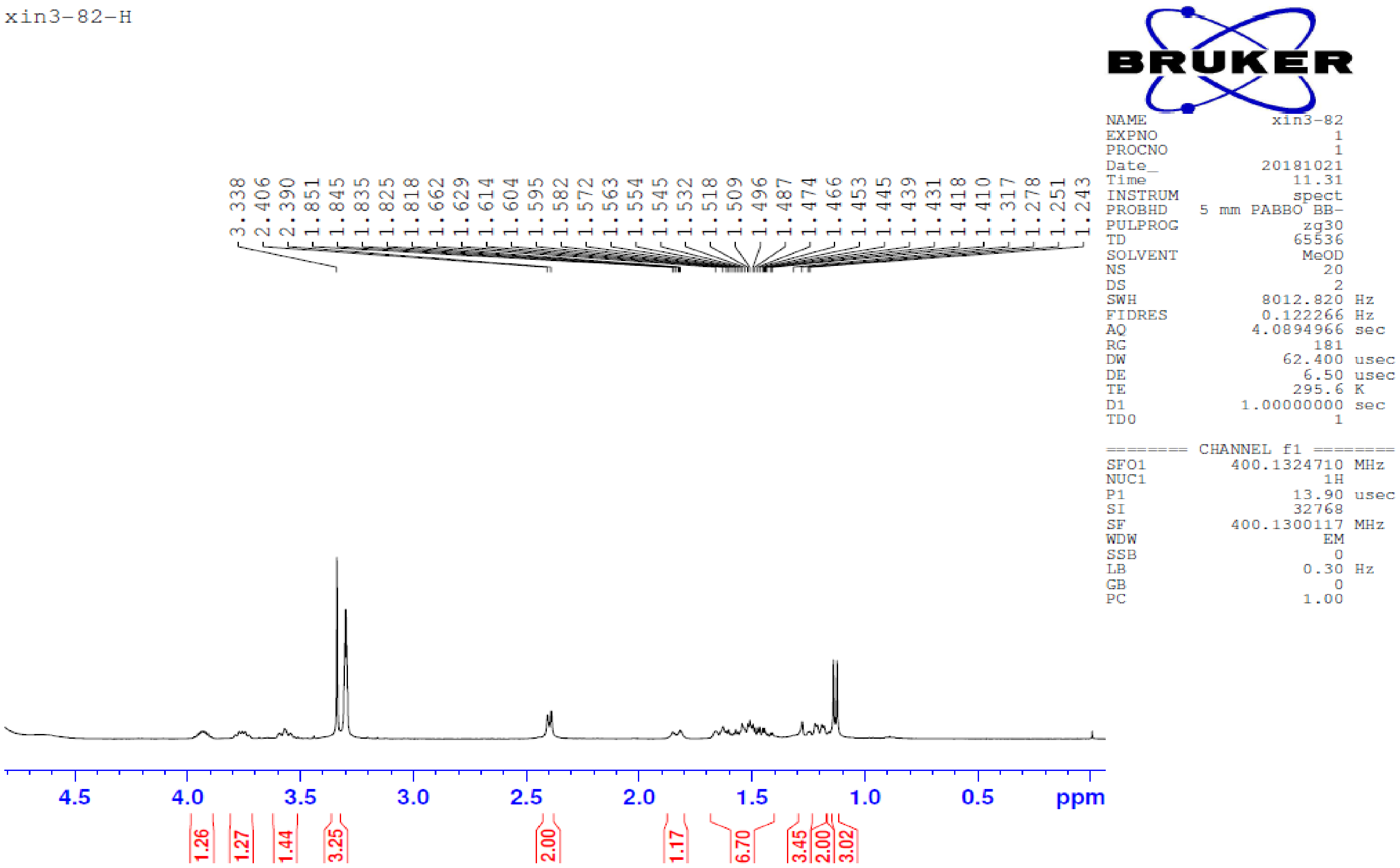

Figure S55. ^{1}H NMR spectrum of **10** in CD_3OD.

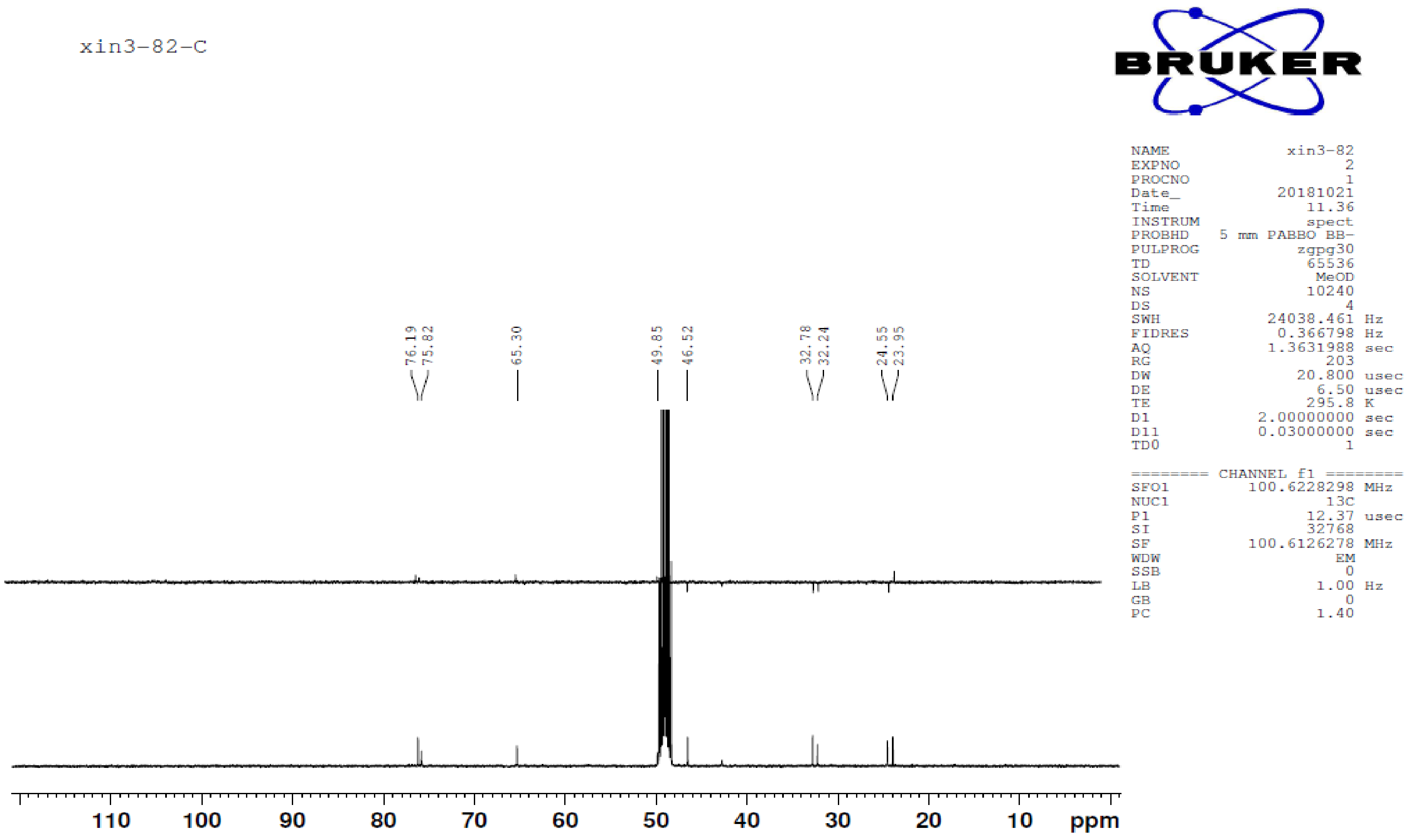

Figure S56. ^{13}C NMR spectrum of **10** in CD_3OD.

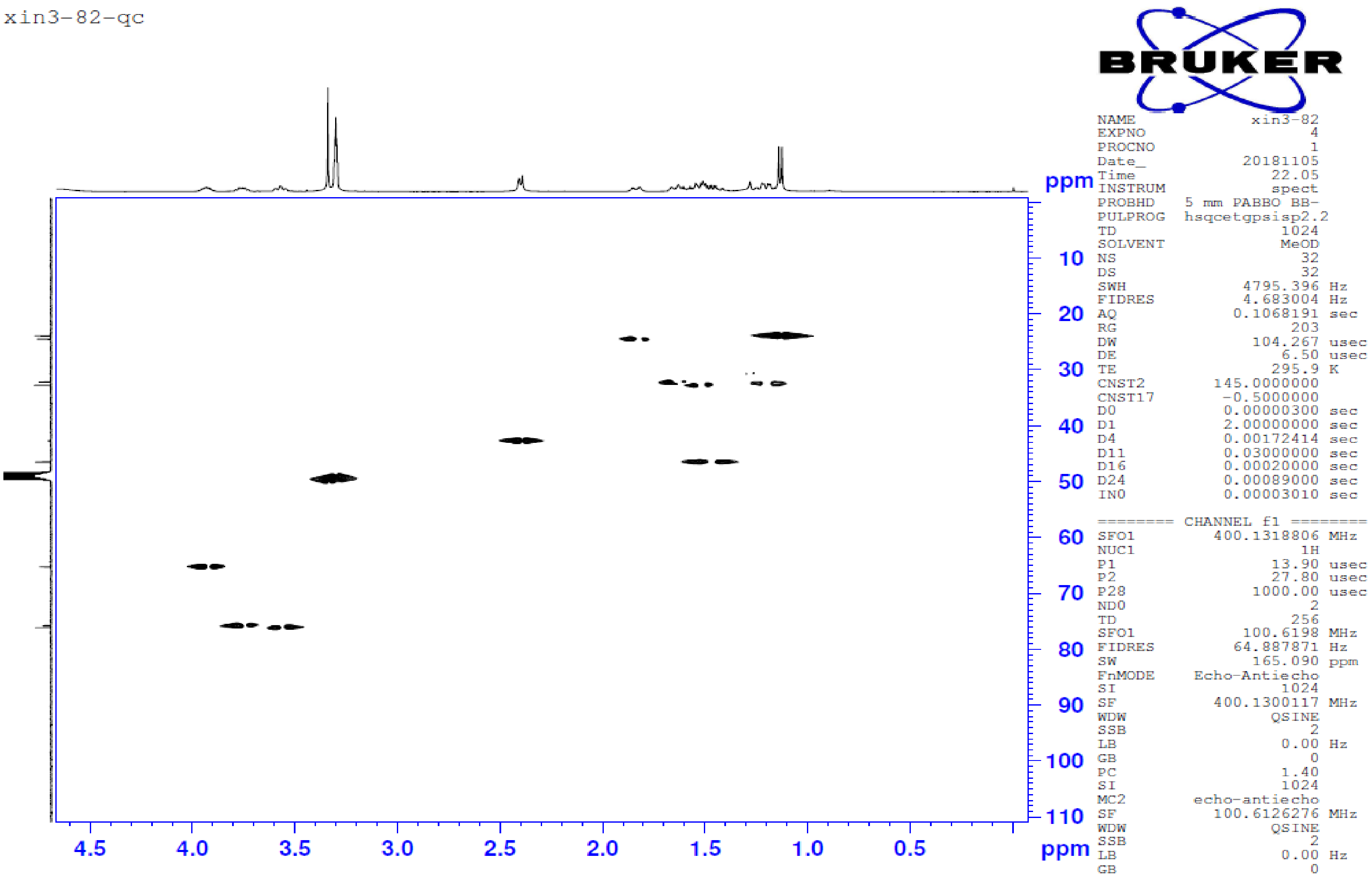

Figure S57. HSQC NMR spectrum of **10** in CD_3OD.

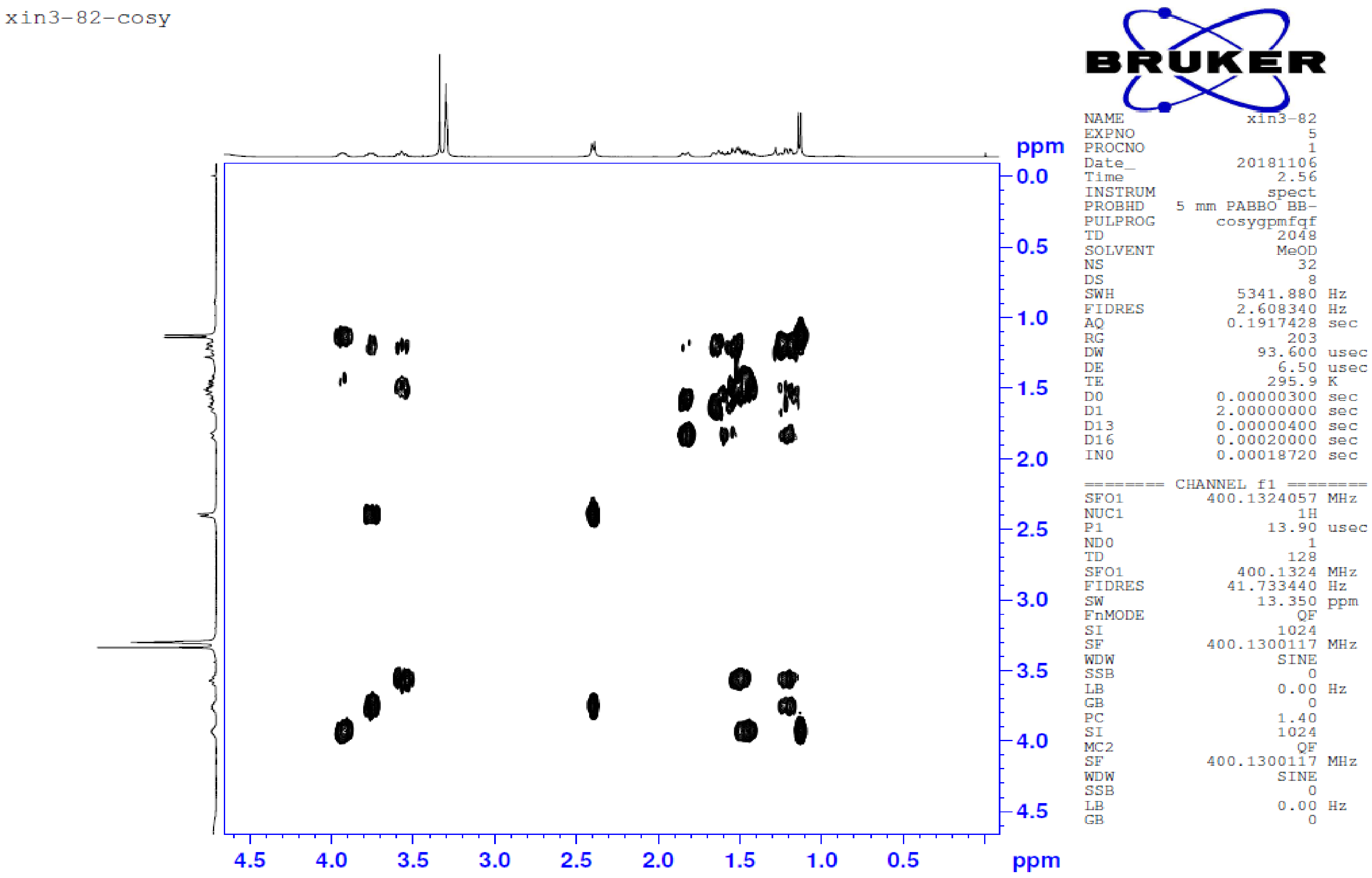

Figure S58. COSY NMR spectrum of **10** in CD_3OD.

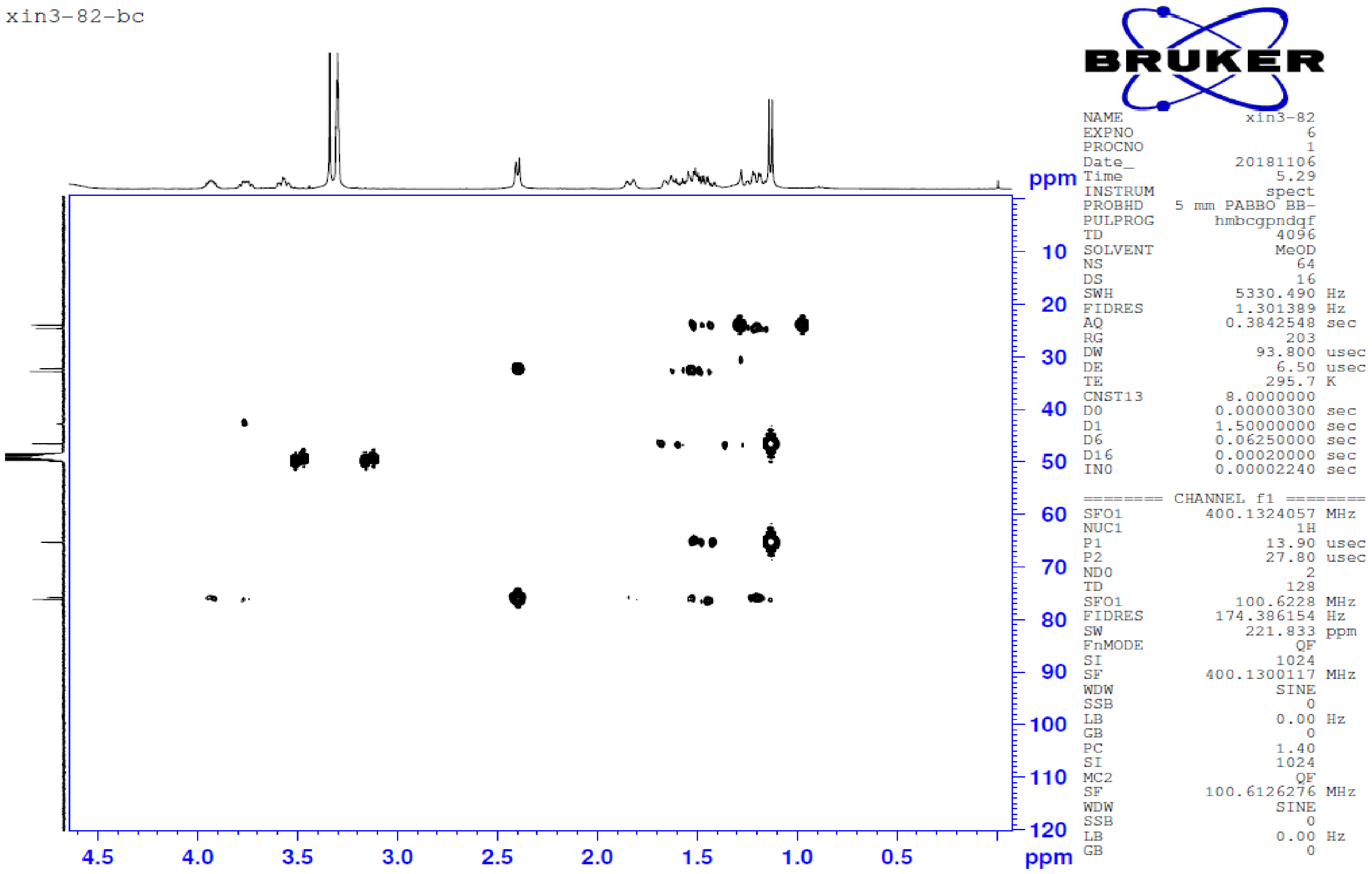

Figure S59. HMBC NMR spectrum of **10** in CD_3OD.

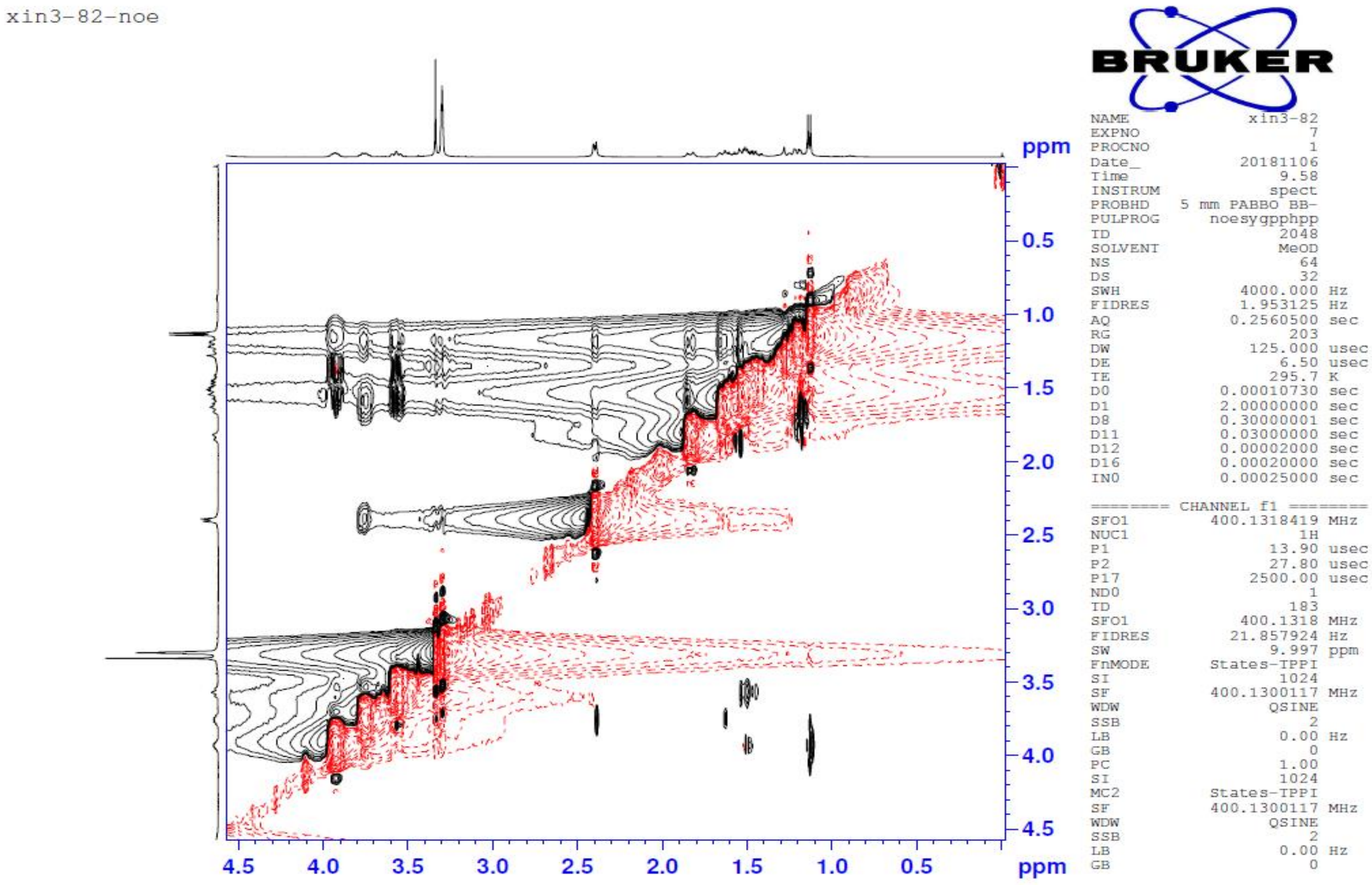

Figure S60. NOESY NMR spectrum of **10** in CD_3OD.
